# Diaryl azo derivatives as anti-diabetic and antimicrobial agents: synthesis, *in vitro*, kinetic and docking studies 

**DOI:** 10.1080/14756366.2021.1929949

**Published:** 2021-07-08

**Authors:** Tehreem Tahir, Mirza Imran Shahzad, Rukhsana Tabassum, Muhammad Rafiq, Muhammad Ashfaq, Mubashir Hassan, Katarzyna Kotwica-Mojzych, Mariusz Mojzych

**Affiliations:** a Institute of Biochemistry, Biotechnology and Bioinformatics, Faculty of Science, The Islamia University of Bahawalpur, Bahawalpur, Pakistan; b Department of Chemistry, Faculty of Science, The Islamia University of Bahawalpur, Bahawalpur, Pakistan; c Department of Physiology and Biochemistry, Faculty of Bio-Sciences, Cholistan University of Veterinary and Animal Sciences, Bahawalpur, Pakistan; d Institute of Molecular Biology & Biotechnology, The University of Lahore (Defense Road Campus), Lahore, Pakistan; e Department of Histology, Embryology and Cytophysiology, Medical University of Lublin, Lublin, Poland; f Department of Chemistry, Siedlce University of Natural Sciences and Humanities, Siedlce, Poland

**Keywords:** Antibacterial, anti-diabetic, azo derivatives, molecular docking, phenolic compounds

## Abstract

In the present study, a series of azo derivatives (**TR-1** to **TR-9**) have been synthesised via the diazo-coupling approach between substituted aromatic amines with phenol or naphthol derivatives. The compounds were evaluated for their therapeutic applications against alpha-glucosidase (anti-diabetic) and pathogenic bacterial strains *E. coli* (gram-negative), *S. aureus* (gram-positive), *S. aureus* (gram-positive) drug-resistant strain, *P. aeruginosa* (gram-negative), *P. aeruginosa* (gram-negative) drug-resistant strain and *P. vulgaris* (gram-negative). The IC_50_ (µg/mL) of **TR-1** was found to be most effective (15.70 ± 1.3 µg/mL) compared to the reference drug acarbose (21.59 ± 1.5 µg/mL), hence, it was further selected for the kinetic studies in order to illustrate the mechanism of inhibition. The enzyme inhibitory kinetics and mode of binding for the most active inhibitor (**TR-1**) was performed which showed that the compound is a non-competitive inhibitor and effectively inhibits the target enzyme by binding to its binuclear active site reversibly.

## 1. Introduction

**Figure 1.**
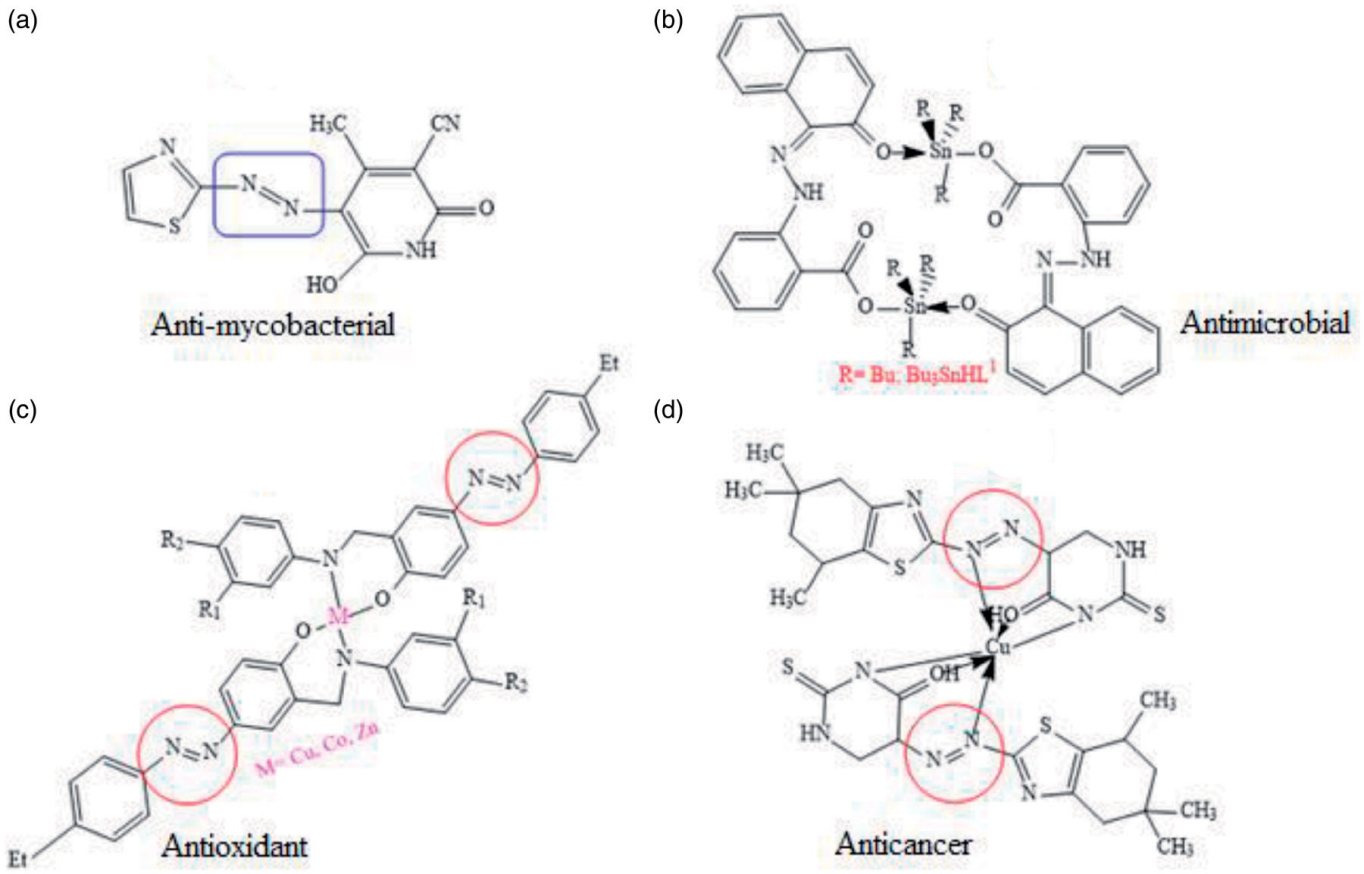
Some reported bioactive azo derivatives^4–7^.

Aromatic azo compounds have been extensively studied in recent years due to their broad-spectrum pharmaceutical applicability mainly because of their cost-effective, simplistic and reproducible synthetic procedure. The combination of aromatic substituted amines with coupling agents could yield a variety of azo compounds having versatile biological properties^1–3^. The presence of heterocyclic ring and transition metals in conjugation to azo pharmacophore enhances the bio-potency of the azo dyes^4^. Ravi and colleagues studied the anti-mycobacterial and DNA cleaving efficiency of 2-aminothiazole incorporated azo derivatives^4^, the most active derivative found to be intervened with the active amino acids of *Mycobacterium tuberculosis*. Debnath et al. evaluated the antimicrobial efficacy of triorganotin(IV) azo-carboxylates^5^ against the bacterium (*S. aureus*) and the fungus (*F. oxysporum*) with significant antimicrobial properties compared to the standard antibiotics. Similarly, Ispir and colleagues examined the anti-oxidant and anti-proliferative potential of azo-azomethine ligands and their metal complexes with Zn(II), Co(II) and Cu(II)^6^. Among the series, the metal complexes bearing azo dyes exhibited the best radical scavenging and inhibitory potencies compared to the standards against the studied cell lines. On the similar interest, Matada et al. prepared a series of heterocyclic azo ligands and their metal complexes with Cu(II), Co(II), Ni(II) and Fe(III)^7^, the synthesised series was therapeutically evaluated for their anti-cancer activity. It was observed that the metal chelates of Cu(II) and Fe(III) were proved to be potential anticancer agents (Figure 1).

Diabetes Mellitus (DM) is a well characterised metabolic disease that results in defects in insulin output i.e. insulin secretion (type-1 diabetes) and insulin action (type-2 diabetes) or both. Type-2 diabetes accounts for 70–80% of all diabetic patients worldwide and it leads to postprandial hyperglycaemia. One of the critical strategies to prevent postprandial hyperglycaemia is to inhibit the enzymes which are responsible for the carbohydrate hydrolysis e.g. α-amylase and α-glucosidase. In this interest, several α-glucosidase inhibitors are being administered in the treatment of type-2 diabetes such as acarbose (valienamine), voglibose (valiolamine) and miglitol (desoxynojirimycin). However, the prolonged use of these agents could result in various side effects such as vomiting, pomposity, diarrhoea and flatulence. Therefore, the discovery and development of new and potent α-glucosidase inhibitors have gained immense attention in pharmaceutical chemistry^8–10^. In this regard, a new derivative from a series of sulphonamide containing diarylpentadienones was found to be a promising inhibitor of α-glucosidase (IC_50_ 5.69 ± 0.5 µM) with the competitive mode of inhibition^11^, some new benzamide derivatives of thiourea (IC_50_ range 20.44 − 333.41 µM) were evaluated as good candidates for targeting α-glucosidase enzyme^12^. The literature also suggested that the derivatives of triazole thiones were found to be effective inhibitors of α-glucosidase (IC_50_ value 36.11 μg/mL)^13^. The recent research has suggested that polyphenols are excellent anti-diabetic agents, recently in this interest; the α-glucosidase inhibitory potential of flavonoids was compared with acarbose with the sequence of inhibitory potential as scutellarein > nepetin > apigenin > hispidulin > acarbose^14^. Dihydropyridine derivatives (IC_50_ range 2.21 ± 0.06 − 9.97 ± 0.08 μM)^15^ have been reported as potential α-glucosidase inhibitors.

Phenolic compounds are simple and naturally occurring organic compounds bearing an aromatic ring with one or more hydroxyl groups (with or without side chain), received considerable attention due to their biological functions such as anti-carcinogen, antimicrobial, anti-ageing, anti-diabetic, anti-mutagen and immunostimulatory roles^16^^,^^17^. Among the most extensively administered phenolic drugs, salbutamol sulphate (SLB), terbutaline sulphate (TBT) and thymol (THY) are highly recognised β_2_-adrenergic receptor agonists, antiseptic and antimicrobial agents respectively^18^. Drug resistance (DR) has become a worldwide threat challenging a wide range of disciplines, including; infection control, drug design and virulence genes. The antimicrobial drug resistance (AMR) develops the manipulative properties of bacteria to counter the standard antibiotics and hence obstructing the treatment of infection. The classes of antibacterial agents which offer resistance to certain bacterial infections constitute the serious therapeutic challenge urging the need for the discovery and development of novel antibiotics^19^^,^^20^. In this regard, a new series of azo-phenol derivatives was synthesised and evaluated *in vitro* against six clinical bacterial strains including the drug-resistant *P. aeruginosa*. It was revealed from the experimental data that some of the azo-phenol compounds significantly inhibited the growth of drug-resistant bacteria compared to the standard drug trimethoprim-sulfamethoxazole^21^. The studies also suggested that phenolic cinnamic acid derivatives are selective inhibitors of cyclooxygenase (COX-1 and COX-2) enzymes^22^, Similarly, phenolic *N*-monosubstituted carbamates have proven to be effective anti-mycobacterial drug targets against broad-spectrum mycobacterial strains (*Mycobacterium tuberculosis* H_37_Ra, H_37_Rv including multidrug and extensively drug-resistant strains, *Mycobacterium avium*, *Mycobacterium kansasii*, *Mycobacterium aurum* and *Mycobacterium smegmatis*)^23^.

**Figure 2.**
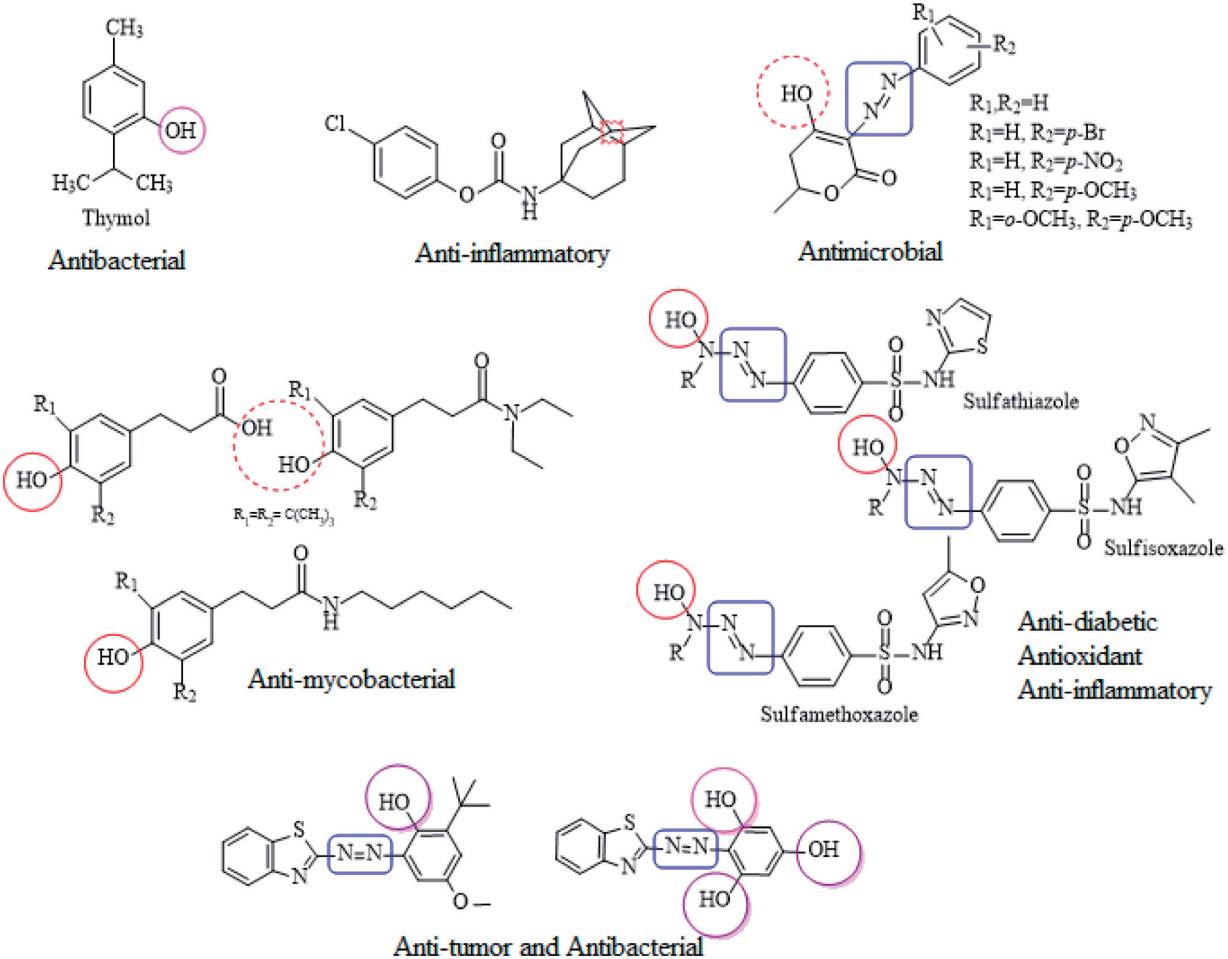
Some of the commercially available and reported phenolic drugs in literature^21–25^.

Hydroxytriazenes are a class of compounds containing alpha hydroxyl group relative to the diazo group, have versatile pharmacological properties including lipid-lowering, antidiabetic, antioxidant, anti-inflammatory, antimicrobial and analgesic agents. In view of this, an attempt has been made to study the anti-diabetic, anti-inflammatory and antioxidant effects of sulpha drugs based hydroxytriazenes^24^. It was suggested that the compounds showed significant α-amylase and α-glucosidase inhibition potential with IC_50_ values ranging from 122–341 µg/mL. The promising anti-inflammatory (89% after 4 h of treatment) and radical scavenging potential (IC_50_ 54.12 µg/mL) highlighted the multifunctional role of hydroxytriazenes. It was also concluded that the polar functionalities like –OH around the heterocyclic rings of triazene compounds enhanced the inhibition potential against glucosidase and amylase^24^. The azo fused with synthetic phenolic molecules such as butylated hydroxyanisole, phlorogucinal, 2,4-di-tert-butylphenol and 2,6-di-tert-butylphenol were proved to be remarkable antibacterial, antioxidant and antitumor agents^25^. Similarly, a series of new phenol ether derivatives were found to be excellent proteasome inhibitors^26^. Some of the reported and commercially available phenolic drugs are given in Figure 2.

Keeping in view the synthetic feasibility of azo derivatives and diverse therapeutic properties of phenolic compounds fused with azo moiety, the present study aims at the synthesis, spectroscopic characterisation, biological evaluation, kinetic and molecular docking studies of diaryl azo-phenol derivatives. The structural identification of the synthesised azo-phenol derivatives was done by spectroscopic techniques (IR, NMR and HRMS) and the detailed screening was carried out by various *in vitro* biological assays with kinetic and *in silico* structure-activity relationship studies.

## 2. Materials and methods

### 2.1. Synthesis

#### 2.1.1. Chemicals and equipment

All solvents and chemicals were of analytical grade and purchased from Sigma-Aldrich and Merck. Melting points were adjusted using a calibrated thermometer by digital melting point apparatus (SMA10 of Stuart Scientific Bibby Sterilin Ltd., UK) and stated in degrees centigrade (°C). Pre-coated silica TLC plates (60F254 Merck Ltd., Japan) for determining purification, magnetic stirrer (VELP Scientific England) for stirring and micropipettes (ZX58143 and ZX57677) for quantitative transfer of liquid were used. The NMR spectra (^1^H and ^13^C) were recorded in DMSO-d_6_ using Varian spectrometer 400 MHz. The high-resolution accurate-mass (HRAM) detection was performed on the Thermo Scientific Q Exactive apparatus. ELISA reader (BioTek) Epoch 2 by Agilent technologies, USA.

#### 2.1.2. General method for the synthesis of azo-compounds

**Scheme 1.**
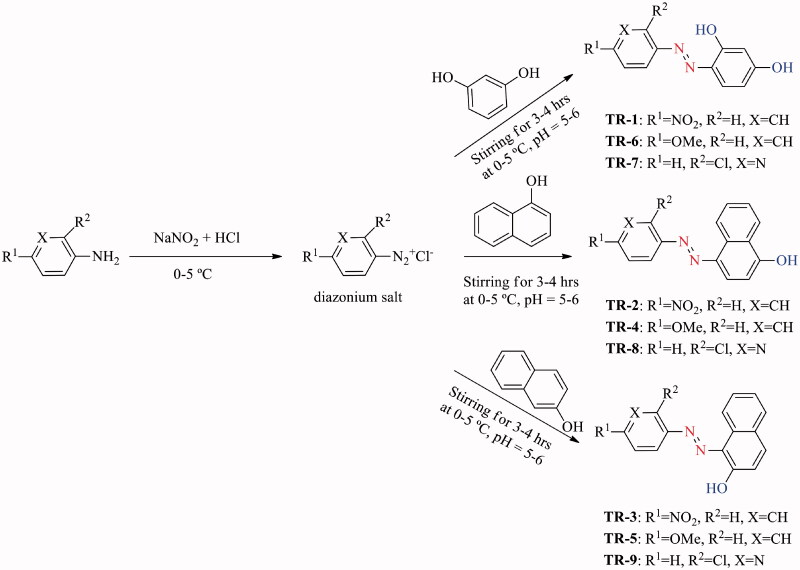
Synthetic scheme employed for the synthesis of azo derivatives (**TR-1** to **TR-9**).

The general procedure for the synthesis of asymmetric aromatic azo compounds followed the traditional approach i.e. the coupling of aryl diazonium salts with electron-rich aromatics such as resorcinol and naphthol derivatives^27^ (Scheme 1). Briefly, equimolar concentration (10 mmol) of substituted amines (4-nitro aniline, *p*-toluidine and 3-amino,2-chloro pyridine) were mixed with the cold aqueous solution of sodium nitrite (10 mmol) in a 100 mL round bottom flask at 0–5 °C. To this reaction mixture, 3 M HCl was added drop-wise with continuous stirring for 20 min until the formation of the diazonium salt at the same temperature. Then immediately, the cold basic solution of respective phenol derivatives (resorcinol, α-naphthol and β-naphthol) (10 mmol) was slowly added to the diazonium salt mixture and the stirring was continued for another 3 h at 0–5 °C. TLC was employed to monitor the progress of the reaction by the combination of various solvent systems. Once the reaction is completed, the resulting crude azo derivatives were filtered off, washed several times with deionised water, air-dried and purified by recrystallization.

##### 2.1.2.1. (*E*)-4-((4-nitrophenyl)diazenyl)benzene-1,3-diol (TR-1)

Orange crystals, yield 83%, melting point: 200–204 °C; *R*_f_*_:_* 0.78 (H_2_O/methanol,7:3); ^1^H-NMR (400 MHz, DMSO-d_6_) *δ*: 6.37 (d, 1H, *J* = 2.4 Hz), 6.51 (dd, 1H, *J*_1_ = 2.4 Hz, *J*_2_ = 8.8 Hz), 7.69 (d, 1H, *J* = 8.8 Hz), 8.05 (d, 2H, *J* = 6.8 Hz), 8.36 (d, 2H, *J* 6.8 Hz), 10.87 (s, 1H, OH exchange with D_2_O), 12.12 (s, 1H, OH exchanged with D_2_O); ^13^C-NMR (100 MHz, DMSO-d_6_) *δ*: 103.10 (Ar-C), 110.17 (Ar-C), 122.48 (Ar-C), 125.04 (Ar-C), 133.35 (Ar-C), 147.12 (C), 154.64 (C), 158.73 (C), 164.93 (C); IR (KBr, cm^−1^): 3441.43 (OH, CH stretch), 1627.41 (C=C), 1514.81 (N=N), 1336.05 (C–N). HR–MS (ESI, *m/z*) Calcd. For C_12_H_9_N_3_O_4_ [M + H] 260.06658. Found [M + H] 260.06653.

##### 2.1.2.2. (*E*)-4-((4-nitrophenyl)diazenyl)naphthalen-1-ol (TR-2)

Red crystals, yield 55%, melting point: 260–265 °C^28^; *R*_f_: 0.82 (H_2_O/methanol, 7:3); ^1^H-NMR (400 MHz, DMSO-d_6_) *δ*: 6.72 (d, 1H, *J* = 9.6 Hz), 7.50 (t, 1H, *J* = 7.2 Hz), 7.61 (t, 1H, *J* = 9.6 Hz), 7.72 (d, 1H, *J* = 9.6 Hz), 7.93–7.98 (m, 3H), 8.32 (d, 2H, *J* = 8.8 Hz), 8.42 (d, 1H, *J* = 8.0 Hz), 15.68 (s, 1H, OH exchanged with D_2_O); ^13^C-NMR (100 MHz, DMSO-d_6_) δ: 117.28 (Ar-C), 122.30 (Ar-C), 125.65 (Ar-C), 126.24 (Ar-C), 127.71 (Ar-C), 128.49 (Ar-C), 129.50 (Ar-C), 129.89 (Ar-C), 131.19 (Ar-C), 132.49 (Ar-C), 143.63 (Ar-C), 143.93 (C), 179.28 (C); IR (KBr, cm^−1^): 3437.49 (OH, CH stretch), 1624.21 (C=C), 1507.92 (N=N), 1402 (C–N). HR–MS (ESI, *m/z*) Calcd. For C_16_H_11_N_3_O_3_ [M + H] 294.08732. Found [M + H] 294.08729.

##### 2.1.2.3. (*E*)-1-((4-nitrophenyl)diazenyl)naphthalen-2-ol (TR-3)

Red crystals, yield 78%, melting point: 245–252 °C^29^; *R*_f_*_:_* 0.86 (H_2_O/methanol, 7:3); ^1^H-NMR (400 MHz, DMSO-d_6_) *δ*: 6.72 (d, 1H, *J* = 9.6 Hz), 7.50 (t, 1H, *J* = 8.0 Hz), 7.61 (t, 1H, *J* = 8.0 Hz), 7.72 (d, 1H, *J* = 8.0 Hz), 7.93–7.98 (m, 3H), 8.32 (d, 2H, *J* = 6.8 Hz), 8.43 (d, 1H, *J* = 7.6 Hz) 15.69 (s, 1H, OH exchanged with D_2_O); ^13^C-NMR (100 MHz, DMSO-d_6_) *δ*: 117.29 (Ar-CH), 122.30 (Ar-C), 125.66 (Ar-C), 126.24 (Ar-C), 127.72 (Ar-C), 128.49 (Ar-C), 129.50 (Ar-C), 129.90 (Ar-C), 131.19 (Ar-C), 132.58 (Ar-C), 143.63 (Ar-C), 143.94 (C), 148.01 (C), 179.28 (C); IR (KBr, cm^−1^): 3432.58 (OH, CH stretch), 1600.21 (C=C), 1507.41 (N=N), 1207.01 (C–N). HRMS (ESI, *m/z*) Calcd. for C_16_H_11_N_3_O_3_ [M + H] 294.08732. Found [M + H] 294.08736.

##### 2.1.2.4. (*E*)-4-(*p*-tolyldiazenyl)naphthalen-1-ol (TR-4)

Red crystals, yield 60%; melting point: 99–100 °C; *R*_f_: 0.77 (acetone/n-hexane, 1:1); ^1^H-NMR (400 MHz, DMSO-d_6_) *δ*: 2.38 (s, 3H), 7.01 (d, 1H, *J* = 9.6 Hz), 7.37 (d, 2H, *J* = 8.4 Hz), 7.46 (t, 1H, *J* = 8.0 Hz), 7.82 (d, 3H, *J* = 8.4 Hz), 7.97 (d, 1H, *J* = 9.2 Hz), 15.60 (s, 1H, OH exchanged with D_2_O); ^13^C-NMR (100 MHz, DMSO-d6) *δ*: 20.89 (–CH), 119.69 (Ar-C), 121.23 (Ar-C), 122.94 (Ar-C), 125.47 (Ar-C), 127.10 (Ar-C), 128.77 (Ar-C), 128.87 (Ar-C), 130.33 (Ar-C), 132.93 (Ar-C), 138.14 (Ar-C), 138.90 (Ar-C), 147.13 (Ar-C), 164.14 (C–OH); IR (KBr, cm^−1^): 3441.80 (OH), 1625.00 (C=C), 1511.02 (N=N), 1280 (C–N). HRMS (ESI, *m/z*) Calcd. For C_17_H_14_N_2_O [M + H] 263.11789. Found [M + H] 263.11796.

##### 2.1.2.5. (*E*)-1-(*p*-tolyldiazenyl)naphthalen-2-ol (TR-5)

Orange-red crystals, yield 46%, melting point: 110 °C^30^; *R*_f_: 0.73 (acetone/n-hexane, 1:1); ^1^H-NMR (400 MHz, DMSO-d_6_) *δ*: 2.38 (s, 3H), 7.01 (d, 1H, *J* = 9.6 Hz), 7.37 (d, 2H, *J* = 8.4 Hz), 7.47 (t, 1H, *J* = 8.0 Hz), 7.62 (t, 1H, *J* = 8.0 Hz), 7.82 (d, 3H, *J* = 8.4 Hz), 7.97 (d, 1H, *J* = 9.2 Hz), 8.60 (d, 1H, *J* = 8.4 Hz), 15.60 (s, 1H, OH exchanged with D_2_O); ^13^C-NMR (100 MHz, DMSO-d_6_) *δ*: 20.89 (–CH), 119.69 (Ar-C), 121.23 (Ar-C), 122.94 (Ar-C), 125.47 (Ar-C), 127.82 (Ar-C), 128.77 (Ar-C), 128.87 (Ar-C), 130.33 (Ar-C), 132.64 (Ar-C), 138.67 (Ar-C), 138.90 (Ar-C), 144.13 (Ar-C), 164.69 (C-OH); IR (KBr, cm^−1^): 3391.78 (OH, CH stretch), 1629.61 (C=C), 1463.84 (N=N), 1281 (C–N). HRMS (ESI, *m/z*) Calcd. For C_17_H_14_N_2_O [M + H] 263.11789. Found [M + H] 263.11780.

##### 2.1.2.6. (*E*)-4-(*p*-tolyldiazenyl)benzene-1,3-diol (TR-6)

Reddish orange crystals, yield 60%, melting point: 98 °C; *R*_f_: 0.8 (acetone/n-hexane, 1:1); ^1^H-NMR (400 MHz, DMSO-d_6_) *δ*: 2.38 (s, 3H), 6.34 (d, 1H, *J* = 2.4 Hz), 6.49 (dd, 1H, *J*1 = 2.4 Hz, *J*2 = 8.8 Hz), 7.34 (d, 1H, *J* = 8.8 Hz), 7.66 (d, 1H, *J* = 8.8 Hz), 7.75 (d, 1H, *J* = 8.0 Hz), 10.50 (s, 1H, OH exchanged with D_2_O), 12.44 (s, 1H, OH exchanged with D_2_O); ^13^C-NMR (100 MHz, DMSO-d_6_) *δ*: 20.96 (–CH), 102.97 (Ar-C), 108.94 (Ar-C), 121.64 (Ar-C), 129.96 (Ar-C), 130.04 (Ar-C), 132.08 (Ar-C), 140.24 (Ar-C), 148.63 (Ar-C), 156.07 (C), 162.64 (C); IR (KBr, cm^−1^): 3476.11 (OH, CH stretch), 1627.19 (C–C=C), 1458.76 (N=N). HRMS (ESI, *m/z*) Calcd. For C_13_H_12_N_2_O_2_ [M + H] 229.09715. Found [M + H] 229.09710.

##### 2.1.2.7. 4-((2-chloropyridin-3-yl)diazenyl)benzene-1,3-diol (TR-7)

Orange-brown crystals, yield 98%; melting point: 187–189 °C; *R*_f_ (methanol/n-hexane, 7:3); 0.80; ^1^H-NMR (400 MHz, DMSO-d_6_) *δ*: 6.38 (d, 1H, *J* = 2.8 Hz), 6.56 (dd, 1H, *J*1 = 2.4 Hz, *J*2 = 9.2 Hz), 7.58 (dd, 1H, *J* = 4.8 Hz, *J*2 = 8.0 Hz), 7.73 (d, 1H, *J* = 8.8 Hz), 8.25 (d, 1H, *J* = 8.0 Hz), 8.47 (d, 1H, *J* = 8.8 Hz), 10.94 (s, 1H, OH exchanged with D_2_O), 12.61 (s, 1H, OH exchanged with D_2_O); ^13^C-NMR (100 MHz, DMSO-d_6_) *δ*: 103.00 (Ar-C), 110.15 (Ar-C), 124.41 (Ar-C), 126.05 (Ar-C), 130.57 (Ar-C), 133.46 (Ar-C), 142.85 (C), 148.07 (C), 150.19 (C), 157.46 (C), 164.76 (C); IR (KBr, cm^−1^): 3403.94 (OH, CH stretch), 1635.34 (C–C), 1575.56 (N=N), 1407 (C–N). HRMS (ESI, *m/z*) Calcd. For C_11_H_8_ClN_3_O_2_ [M + H] 250.03778. Found [M + H] 250.03790.

##### 2.1.2.8. 4-((2-chloropyridin-3-yl)diazenyl)naphthalen-1-ol (TR-8)

Orange crystals, yield 71%, melting point: 170–175 °C; *R*_f_ = 0.81 (methanol/n-hexane, 7:3); ^1^H-NMR (400 MHz, DMSO-d_6_) *δ*: 6.78 (d, 1H, *J* = 9.6 Hz), 7.47 (t, 1H, *J* = 9.6 Hz), 7.56–7.60 (m, 2H), 7.71 (d, 1H, *J* = 9.6 Hz), 7.95 (d, 1H, *J* = 9.6 Hz), 8.30 (d, 1H, *J* = 8.0 Hz), 8.47 (t, 2H, *J* = 8.0 Hz); ^13 ^C-NMR (100 MHz, DMSO-d_6_) *δ*: 122.11 (Ar-C), 124.84 (Ar-C), 125.14 (Ar-C), 125.24 (Ar-C), 127.29 (Ar-C), 128.29 (Ar-C), 129.34 (Ar-C), 129.73 (Ar-C), 131.58 (Ar-C), 132.31 (Ar-C), 137.16 (Ar-C), 139.87 (Ar-C), 142.81 (C), 146.46 (C), 175.47 (C); IR (KBr, cm^−1^): 3301.93 (OH, CH stretch), 1631.42 (C=C), 1513.94 (N=N), 1332 (C–N). HRMS (ESI, *m/z*) Calcd. For C_15_H_10_ClN_3_O [M + H] 284.05852. Found [M + H] 284.05845.

##### 2.1.2.9. 4-((2-chloropyridin-3-yl)diazenyl)naphthalen-2-ol (TR-9)

Reddish orange crystals, yield 70%; melting point: 180–182 °C; *R*_f_: 0.75 (methanol/n-hexane, 7:3); ^1^H-NMR (400 MHz, DMSO-d_6_) *δ*: 6.79 (d, 1H, *J* = 9.6 Hz), 7.47 (t, 1H, *J* = 8.0 Hz), 7.57–7.60 (m, 2H), 7.72 (d, 1H, *J* = 7.2 Hz), 7.95 (d, 1H, *J* = 9.6 Hz), 8.30 (d, 1H, *J* = 8.4 Hz), 8.48 (t, 2H, *J* = 8.0 Hz); ^13^C-NMR (100 MHz, DMSO-d_6_) *δ*: 122.14 (Ar-C), 124.87 (Ar-C), 125.17 (Ar-C), 125.27 (Ar-C), 127.33 (Ar-C), 128.31 (Ar-C), 129.38 (Ar-C), 129.78 (Ar-C), 131.61 (Ar-C), 132.33 (Ar-C), 137.19 (Ar-C), 139.89 (Ar-C), 142.85 (C), 146.50 (C), 175.51 (C); IR (KBr, cm^−1^): 3475 (OH), 1626 (C=C), 1456 (N=N), 1208 (C–N). HRMS (ESI, *m/z*) Calcd. for C_15_H_10_ClN_3_O [M + H] 284.05852. Found [M + H] 284.05862.

### 2.2. *In vitro* α-glucosidase inhibition assay

The α-glucosidase inhibition assay was performed as described previously^31^. In summary, the stock solution of the enzyme from *Saccharomyces Cerevisiae* (0.2 U/mL) was prepared in 0.05 M sodium phosphate buffer pH 6.8. In microtitre plate, 70 µL of phosphate buffer, 10 µL of enzyme and 10 µL of varying concentrations of azo derivatives (25 µg, 12.5 µg, 6.25 µg, 3.12 µg and 1.56 µg/mL) were mixed and incubated at 37 °C for 15 min. After incubation, 10 µL of the substrate, *p*-nitrophenyl α-D-glucopyranoside (pNPG, 5 mM solution in 0.05 M phosphate buffer) was added to the aboveprepared mixture and incubated again under the same experimental conditions. The reaction was stopped with the addition of 0.1 M Na_2_CO_3_ and OD_405_ was taken by a 96-well ELISA reader. The experiment was performed in triplicate. Acarbose was used as positive control while DMSO was a negative control. The extent of inhibition showed by the tested azo derivatives was calculated by % inhibition formula and IC_50_ (µg/mL) was determined by Graph pad Prism 6.0.

### 2.3. Kinetic analysis of α-glucosidase inhibition

A series of kinetic assays was performed to determine the inhibition kinetics of the most active inhibitor from the series of tested compounds by following method^32^^,^^33^. The potential inhibitor with concentrations 0.00, 0.062, 0.125, 0.25, 0.5 mM was selected for kinetic studies. The substrate (pNPG) concentration was optimised from 5 to 40 mM in all kinetic studies. Pre-incubation and measurement time was the same as discussed in α-glucosidase inhibition assay. The assay was continuously monitored at 405 nm for 5 min at 30 s intervals in the microplate reader after the addition of the enzyme. The inhibition type on the enzyme was assayed by Lineweaver–Burk plots of the inverse of velocities (1/*V*) versus the inverse of substrate concentration 1/[*S*] mM^−1^, and the inhibition constant *K*_i_ was determined by two ways (Dixon plot of 1/*V* versus inhibitor concentrations) as well as secondary replot of slope versus inhibitor concentrations from Lineweaver–Burk plot.

### 2.4. Inhibition mechanism of potential inhibitor

The inhibitory mechanism against α-glucosidase by most active inhibitor from the tested series of azo derivatives was investigated. The plot of the remaining enzyme activity versus the concentrations of the enzyme at different inhibitor concentrations (0.00, 0.062, 0.125, 0.25 and 0.5 mM) was calculated.

### 2.5. *In silico* molecular docking

#### 2.5.1. Retrieval of α-glucosidase from protein data bank (PDB)

The crystal structure of α-glucosidase having PDBID: 4J5T was accessed from the Protein Data Bank (PDB) (http://www.rcsb.org/4j5t). The retrieved protein structure was minimised by using the conjugate gradient algorithm and amber force field in UCSF Chimaera 1.10.1^34^. The stereo-chemical properties, Ramachandran graph and values^35^ of α-glucosidase were assessed by Molprobity server^36^, while the Ramachandran graph was generated by Discovery Studio 2.1 Client. The protein architecture and statistical percentage values of helices, beta-sheets, coils and turns were accessed by VADAR 1.8^37^.

#### 2.5.2. *In silico* drugs designing

The designed ligands (**TR1**-**9**) were sketched in drawing ACD/ChemSketch tool and retrieved in PDB format. Furthermore, the UCSF Chimaera 1.10.1 tool was employed to energy minimisation of each drug separately having default parameters such as steepest descent steps 100 with step size 0.02 (Å), conjugate gradient steps 100 with step size 0.02 (Å) and update interval was fixed at 10. Finally, Gasteiger charges were added using Dock Prep in drugs to obtain a good structural conformation for docking analysis. A molecular docking experiment was employed on synthesised ligands against α-glucosidase by using PyRx virtual screening tool with AutoDock VINA Wizard approach ^38^. The grid box centre values were adjusted as for *X*= −19.2552, *Y*= −21.0275 and *Z* = 4.2779, respectively. We have adjusted sufficient grid box size big enough on biding pocket residues to allow the ligand to move freely in the search space. The default exhaustiveness value = 8 was adjusted in both dockings to maximise the binding conformational analysis. In all docked complexes, the drug’s conformational poses were keenly observed to obtain the best docking results. The docked complexes were evaluated on the lowest binding energy (Kcal/mol) values and structure-activity relationship analyses. The graphical depictions of all the docking complexes were carried out using Discovery Studio (2.1.0).

### 2.6. Antibacterial activity

The prepared azo derivatives were screened for their antibacterial activity against six bacterial strains obtained from hospital isolates i.e. gram positive (*S. aureus*, *S. aureus* (DR)) and gram-negative (*E. coli*, *P. aeruginosa*, *P. aeruginosa* (DR), *P. vulgaris*). All the strains were confirmed by biochemical identification and drug susceptibility methods. The antibacterial evaluation was done by agar disc diffusion method^39^^,^^40^ and MIC was determined^41–43^. In summary, a 48-h old inoculum having ∼0.48 OD_600_, of the above-mentioned bacterial strains was spread on the solidified trypticase soy agar media and Whatman no. 1 filter paper discs (5 mm in diameter) soaked with the title compounds (10 µL/disc of 10 mg/mL) were placed on the plates. Ampicillin and moxifloxacin were used as positive controls and DMSO as negative control respectively. The plates were incubated at 37 °C for 48 h, post-inoculation and diameters for the zone of inhibition were calculated in millimetres (mm) and MIC (µg/mL) was determined for the most active compounds.

### 2.7. Antibiofilm assay

The antibiofilm activity of the synthesised azo derivatives was assessed by microtitre plate-based crystal violet method^44^ against respective bacterial strains i.e. gram-positive (*S. aureus*, *S. aureus* (DR)) and gram-negative (*P. aeruginosa*, *P. aeruginosa* (DR)). In brief, 10 µL bacterial suspension, 100 µL trypticase soy broth and 10 µL (10 mg/mL) of test compounds were added in the wells in duplicates and incubated at 37 °C overnight. Ampicillin and moxifloxacin were used as positive controls. After the aspiration of planktonic cells, the biofilm was fixed with 99% methanol. The wells were then washed with 0.1 M sterile PBS (pH = 6.9) thrice and air-dried. Then, 200 µL of crystal violet solution (2%) was added to all wells. After 15 min, the excess of the crystal violet dye was removed and the wells were washed again with PBS. Then, the cell-bound crystal violet was dissolved in 33% acetic acid. The biofilm growth was quantified in terms of OD_575_ nm using an ELISA plate reader and biofilm hydrolysis was calculated by % inhibition formula.

## 3. Results and discussion

### 3.1. Chemistry

The targeted diaryl aromatic azo compounds were synthesised by the facile diazo-coupling approach (Scheme 1) described previously in literature^45–48^. The combination of the substituted amines (4-nitro aniline, *p*-toluidine and 3-amino,2-chloro pyridine) with phenol derivatives (resorcinol, α-naphthol and β-naphthol) produced azo-phenol compounds in moderate to good yields (40–80%). The purification of the synthesised compounds was achieved through re-crystallization and TLC was employed to confirm the purification status. The structures of the purified diaryl azo-phenol derivatives were elucidated by FTIR, ^1^H-NMR, ^13^C-NMR and mass spectrometry (HRMS). The FTIR spectra were recorded in the range of 4000–600 cm^−1^, the characteristic prominent peaks of synthesised azo-phenol derivatives were observed. The disappearance of NH_2_ stretch at ∼3500 cm^−1^, a broad OH stretch overlapped with the aromatic C–H stretching vibrations around 3300–3000 cm^−1^ and a characteristic N=N peak at ∼1500 have been observed in all FTIR spectra indicating the presence of the said functional groups. In the ^1^H-NMR spectra, recorded in deuterated DMSO, the disappearance of NH_2_ signal in the range of 5.0−4.0 ppm characteristic for the used amines was observed, while the amount and type of signals in the range of 6.0−9.0 ppm correspond exactly to the number of aromatic protons. Similarly, the numbers of signals in the ^1 ^C-NMR spectra for the obtained derivatives were consistent with their structure. High-resolution mass spectrometry (HRMS) was also used to identify and confirmed the structure of azo compounds. The obtained spectroscopic data was found consistent with the reported literature in this regard^48–50^. All detailed spectra (FTIR, ^1^H and ^13^C-NMR, HRMS) are given in Supplementary material.

### 3.2. Inhibitory potential against α-glucosidase

All the title compounds (**TR-1** to **TR-9**) were evaluated for their inhibitory potential against α-glucosidase enzyme. At a concentration of 250 µg/mL, almost all the tested azo-phenol derivatives exhibited 60–98% anti-diabetic activity, except **TR-4** (43%) and **TR-5** (56%). It was noticed that the diaryl azo derivatives with two hydroxyl groups at ortho and meta-positions (**TR**-**1**, **TR**-**6**, **TR**-**7**) have enhanced α-glucosidase inhibition potential with IC_50_ values 15.70 ± 1.3, 53.7 ± 3.2 and 29.7 ± 2.8 respectively, compared to the other active derivatives (**TR**-**2**, **TR**-**3**, **TR**-**4** and **TR**-**5**) with one hydroxyl group at any position on the phenyl ring. The present results were found to be in good agreement with the previous studies establishing the fact that the number and position of hydroxyl groups on the phenyl ring could increase or decrease the inhibitory activity of the compounds against α-glucosidase by effecting the magnitude of hydrogen bond interactions with the protein^51^. On the other hand, in the case of diaryl azo derivatives **TR**-**7**, **TR**-**8** and **TR**-**9**, the notable anti-diabetic potential could possibly be due to the introduction of the nitrogen-containing heterocyclic pharmacophore^13^ with 4–Cl group. The promising anti-diabetic potential exerted by the derivatives **TR**-**7**, **TR**-**8** and **TR**-**9** is also justified by the minimum binding energy values −7.50, −8.20 and −8.30 Kcal/mol respectively. The following trend in the decreasing order of IC_50_ (µg/mL) was observed: **TR**-**5**>**TR**-**3**>**TR**-**2**>**TR**-**9**>**TR**-**6**>**TR**-**8**>**TR**-**7**>**TR**-**1**. The IC_50_ of **TR-1** was found to be effective (15.70 ± 1.3 µg/mL) compared to the reference drug acarbose (21.59 ± 1.5 µg/mL) (Table 1), hence, it was further selected for the kinetic and docking studies in order to illustrate the mechanism of inhibition.

**Table 1.** *In vitro* α-glucosidase inhibitory potential of diaryl azo-phenol derivatives

No.	Sample code	% Inhibition					IC_50_ (µg/mL)	Binding energy (Kcal/mol)
		250 (µg/mL)	125 (µg/mL)	62.5 (µg/mL)	31.2 (µg/mL)	15.62 (µg/mL)		
1	**TR-1**	95	88	78	68	49	15.70 ± 1.3	–7.80
2	**TR-2**	60	55	42	30	16	110.2 ± 7.8	–8.20
3	**TR-3**	62	54	38	25	12	116.9 ± 8.6	–8.00
4	**TR-4**	43	35	27	13	7	nt	nt
5	**TR-5**	56	49	35	22	14	153.4 ± 11.5	–8.40
6	**TR-6**	87	63	55	38	22	53.7 ± 3.2	–7.60
7	**TR-7**	87	77	68	52	34	29.7 ± 2.8	–7.50
8	**TR-8**	98	75	63	45	29	36.7 ± 3.1	–8.20
9	**TR-9**	78	63	58	39	22	54.4 ± 3.3	–8.30
10	Acarbose	92	85	73	62	40	21.59 ± 1.5	nt

Nt: mean not determined. IC_50_: 50% inhibitory concentration (means ± SEM of triplicate experimental values).

### 3.3. Kinetic analysis

To understand the inhibitory mechanism of the most potent compound against α-glucosidase, kinetic studies were conducted. Kinetic studies showed a concentration-dependent inhibition of α-glucosidase by the inhibitors. Continuous monitoring of the reaction showed a marked decrease in reaction rate in the presence of the inhibitors, which is ultimate, indicated the decrease in the final absorbance when compared with controls containing no inhibitor. The potency of inhibition exhibited by the compounds varied depending on the presence and position of different substitutions and on the class of compounds Inhibition kinetics was analysed by Lineweaver–Burk plot and Dixon plots to determine the type of inhibition and inhibition constant (*K*_i_).

**Figure 3.**
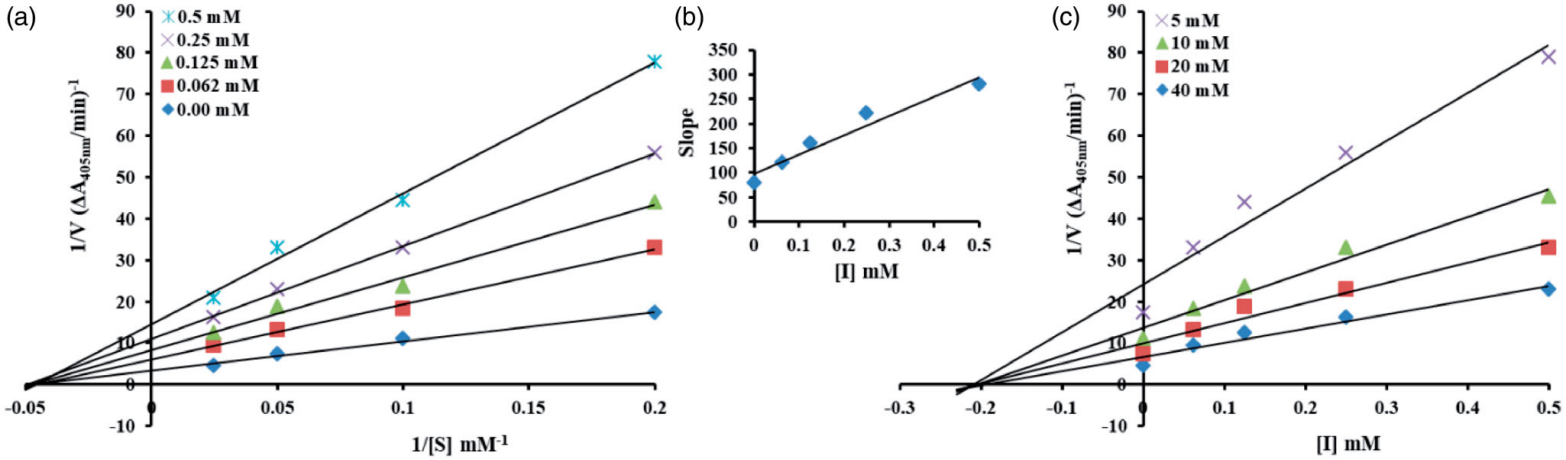
**TR-1 a)** Lineweaver-Burk plots for the inhibition of α-Glucosidase by various concentrations 0.00, 0.062, 0.125, 0.25 and 0.5 mM of compound **TR-1** in the presence of different concentrations 5, 10, 20 and 40 mM of substrate pNPG (p-nitrophenyl-α-D-glucopyranoside). **b)** Secondary replot of the Lineweaver–Burk plot between the slopes of each line on Lineweaver–Burk plot vs. different concentrations of inhibitor. **c)** Dixon plot of reciprocal of rate of reaction (velocities) versus different concentrations of compounds **TR-9** in the presence of various concentrations (5, 10, 20 and 40 mM) of pNPG.

**Figure 4.**
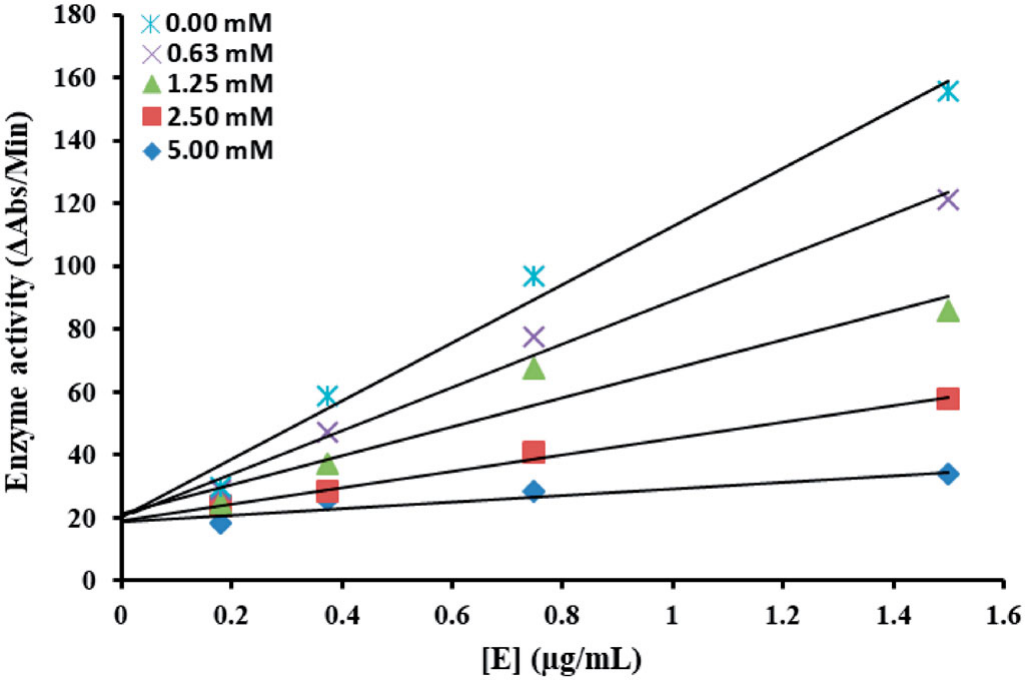
The catalytic activity of α-glucosidase as a function of enzyme concentration at different concentrations of compound **TR-1**.

From the kinetic analyses, the Lineweaver–Burk plot of 1/*V* versus 1/[*S*] in the presence of different concentrations of **TR-1** showed that all the straight line intersects on the X-axis in the second quadrant, the analysis showed that *V_max_* decreased with constant *K_m_* in the presence of increasing concentrations of compounds **TR-1**. This behaviour of the compound indicated that the compound **TR-1** is a non-competitive inhibitor against α-glucosidase (Figure 3(a))^32^. The inhibition constant *K*_i_ for compounds **TR-1** was calculated by two methods, secondary replot of slope from Lineweaver-Burk plot versus inhibitor concentrations (Figure 3(b)) and by Dixon plot as shown in (Figure 3(c)) respectively. All the kinetics parameters are written in Table 2. The inhibitory mechanism of α-glucosidase by varying concentrations of the most potent compound **TR-1** by the plots of the remaining enzyme activity versus the enzyme concentration was also studied. The results suggested that the compound behaves reversibly (Figure 4)^52^.

**Table 2.** Kinetic parameters of α-glucosidase inhibition activity for the compound **TR-1**.

Compounds	Dose (mM)	*V*_max_ (Δ*A*/S)	*K*_m_ (mM)	Inhibition type	Inhibition	*K*_i_ (mM)
TR-1	0.00	0.25	20	Non-competitive	reversible	0.22
	0.062	0.166	20			
	0.125	0.125	20			
	0.25	0.090	20			
	0.50	0.071	20			

*V*_max_ is the reaction velocity.

*K*_m_ is the Michaelis–Menten constant.

*K*_i_ is the EI dissociation constant.

### 3.4. Molecular docking analyses

#### 3.4.1 α-Glucosidase structural assessment

**Figure 5.**
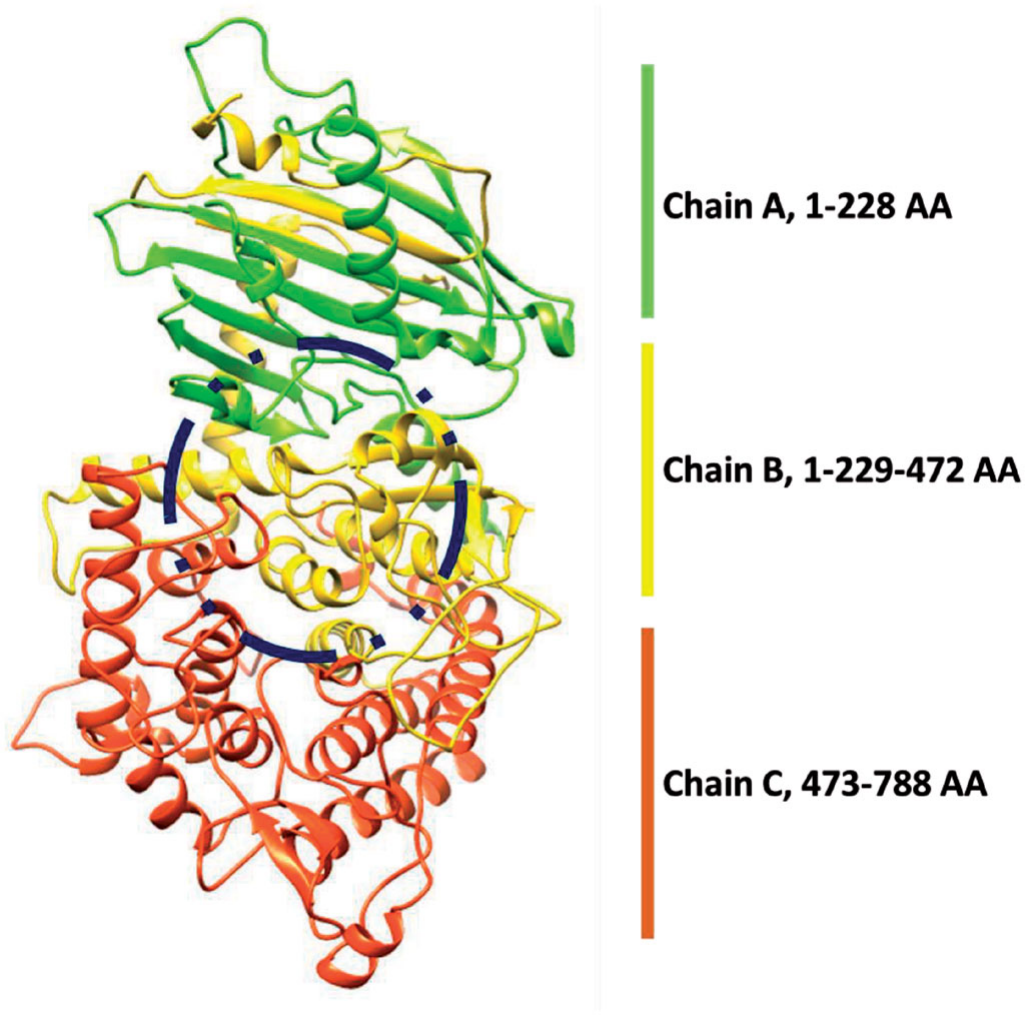
3D structure of α-glucosidase enzyme.

The α-glucosidase is a class of hydrolase enzyme and is actively involved in the digestion of food carbohydrates. The α-glucosidase comprises 811 amino acids with the residual architecture of 35% helices, 25% β sheets and 38% coils. The X-ray diffraction studies of α-glucosidase confirmed its resolutions 2.04 Å. The Ramachandran plots of α-glucosidase indicated that 97.6% of residues were present in favored regions. The Ramachandran graphs values showed the good accuracy of phi (φ) and psi (ψ) angles among the coordinates of target protein (Figure 5)

#### 3.4.2. Binding pocket and deltamethrin and permethrin binding conformations

A molecular docking experiment is the best approach to study the binding conformation of ligands against target protein^53–55^. The binding pocket analysis showed that deltamethrin and permethrin were confined in the active region of α-glucosidase. Results showed that the drugs bound in the active site having different conformational poses. The docked complexes of the drug against tyrosinase were analysed on the basis of the lowest binding energy values (Kcal/mol) and hydrogen/hydrophobic interaction pattern. Results showed that all the drugs exhibited good docking energy values and showed their interaction within the active region of the target protein (Figure 5). The docking energy values of all the docking complexes were calculated by using Equation (1).

(1) ΔGbinding=ΔGgauss+ΔGrepulsion+ΔGhbond+ΔGhydrophobic+ΔGtors

Here, Δ*G*gauss: attractive term for dispersion of two gaussian functions, Δ*G*repulsion: square of the distance if closer than a threshold value, Δ*Gh*bond: ramp function – also used for interactions with metal ions, Δ*G*hydrophobic: ramp function, Δ*G*tors: proportional to the number of rotatable bonds. In docking, energy results compound selected as best having more than 2.5 Kcal/mol energy value different compared to other compounds. The standard error for Autodock is reported as 2.5 Kcal/mol (http://autodock.scripps.edu/)^56^. The present docking results justified that the energy value difference among all docking complexes was comparable with the standard error value. Therefore, based on the basis of both *in vitro* and *in silico* docking energy results, deltamethrin and permethrin were ranked as the best drugs that showed good inhibitory potential against targeted enzyme as compared to all other derivatives. The docking energy values of the synthesised ligands (**TR1**-**9**) against α-glucosidase are presented in Table 1.

#### 3.4.2. Binding pocket and hydrogen binding analysis

**Figure 6.**
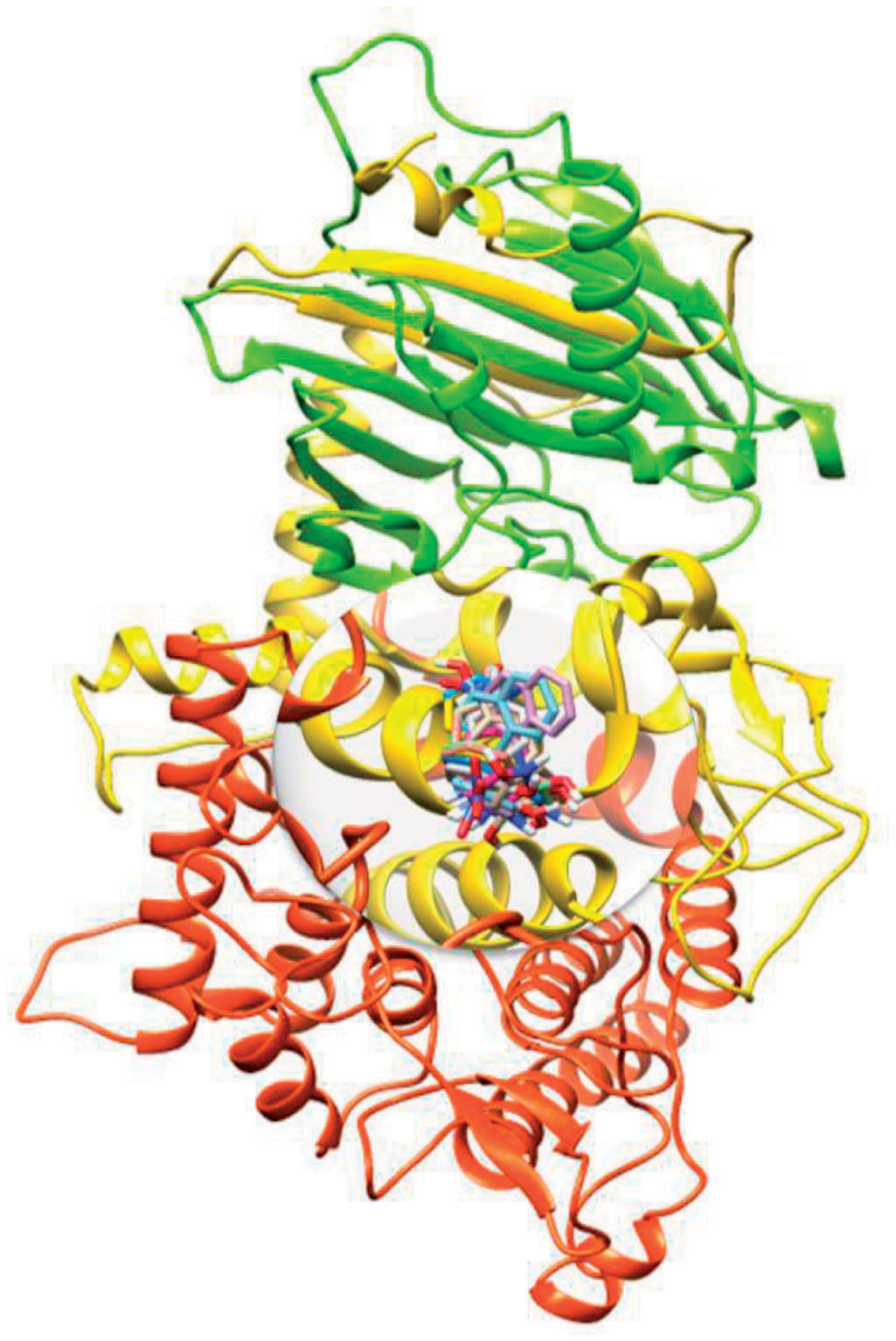
Binding pocket prediction and interactions of all ligands (**TR1-9**) against α-glucosidase.

Figure 6 showed the binding pocket prediction and interactions of all ligands at conformational positions. All the ligands (**TR1-9**) bind within the active region of α glucosidase which ensures the stability and reliability of docking results. In most of the ligands, oxygen atoms were confined at the outer region of the binding pocket whereas, the benzene ring part showed inside the binding pocket.

**Figure 7.**
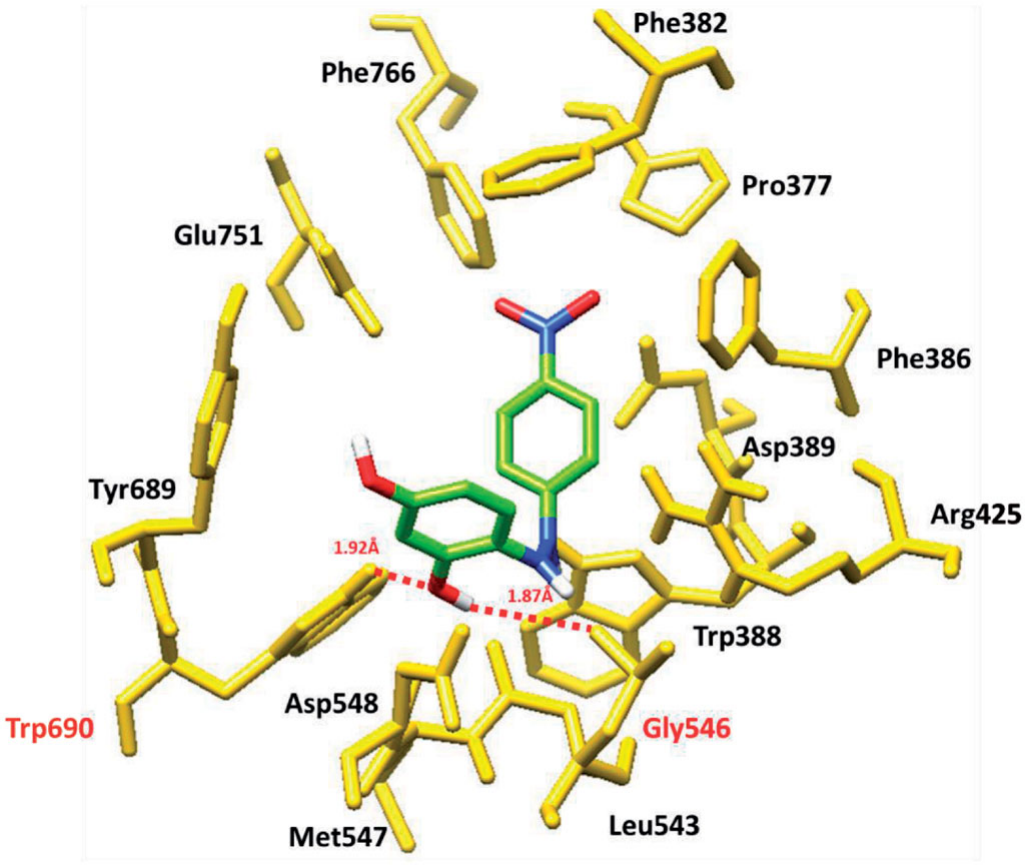
Molecular docking interaction of **TR**-**1** against target protein.

Based on *in vitro* results, **TR-1** has selected for detail binding interactions. The docking results showed that **TR-1** formed two at different residual positions hydrogen bond. The hydroxyl group of **TR-1** formed a hydrogen bond with Gly546 and Trp690 having bond distances of 1.87 and 1.92 Å, respectively. In docking results, **TR-1** binds within the active site and the bond lengths were comparable with the standard value (<3 Å) (Figure 7). Our docking results were well correlated with a previously published article, confirming the accuracy of our docking results^57^.

Supplementary data (S1): The α-glucosidase Ramachandran graph is presented the residual position in the favour region (pink boundary) by evaluating the Psi (φ) and Phi (ψ) angles. Most of the residues (green circle) lie in favour region while only ten poor rotamers are present in the outer region.

### 3.5. Antimicrobial potential

The antibacterial results of the prepared azo-phenol derivatives are displayed in (Table 3) and are expressed quantitatively in MIC (µg/200 µL). The antimicrobial results of synthesised azo derivatives exhibited good to moderate broad-spectrum activity against screened pathogens (*S. aureus*, *P. aeruginosa*, *E. coli* and *P. vulgaris*). Among the studied title compounds, **TR-1, TR-4, TR-6** displayed promising antibacterial potential with an average MIC value of 125 µg/200 µL and was found to be dose-dependent, compared to the standard drugs i.e. ampicillin and moxifloxacin (MIC 62.5 µg/200 µL). It was also observed that the detrimental effect of derivatives on bacterial strains was due to the electron-withdrawing groups on the phenyl rings^4^.

**Table 3.** Minimum inhibitory concentration (µg/200 µL) of azo-phenol derivatives against below mentioned bacterial strains

Compound code	*S. aureus*(DS)			*S. aureus*(DR)			*P. aeruginosa*(DS)			*P. aeruginosa*(DR)			*E. coli*			*P. vulgaris*		
	250	125	62.5	250	125	62.5	250	125	62.5	250	125	62.5	250	125	62.5	250	125	62.5
**TR-1**	S	S	R	R	R	R	R	R	R	S	S	S	S	S	S	S	S	S
**TR-2**	R	R	R	S	S	R	S	R	R	R	R	R	S	R	R	R	R	R
**TR-3**	R	R	R	R	R	R	S	R	R	R	R	R	R	R	R	R	R	R
**TR-4**	S	S	S	S	S	R	S	S	R	S	S	R	R	R	R	S	S	S
**TR-5**	R	R	R	S	S	S	S	R	R	S	S	R	S	S	S	R	R	R
**TR-6**	S	S	S	S	S	S	S	S	R	R	R	R	S	R	R	S	S	S
**TR-7**	R	R	R	R	R	R	R	R	R	S	S	S	R	R	R	R	R	R
**TR-8**	S	S	R	R	R	R	S	R	R	R	R	R	R	R	R	R	R	R
**TR-9**	R	R	R	S	S	S	S	R	R	R	R	R	R	R	R	R	R	R
Ampicillin	S	S	S	--	--	--	S	S	S	--	--	--	S	S	S	S	S	S
Moxifloxacin	--	--	--	S	S	R	--	--	--	S	S	R	--	--	--	--	--	--

R: resistant; S: sensitive; –: not tested.

### 3.6. Biofilm inhibition potency

**Figure 8.**
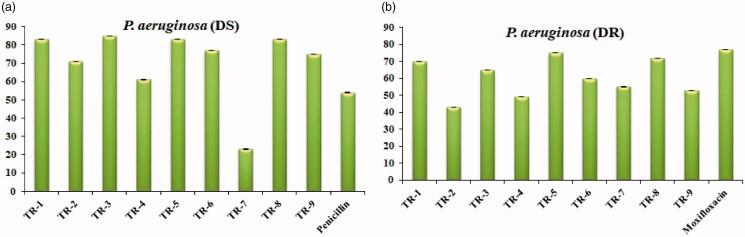
Biofilm hydrolysis potential of azo derivatives against *P. aeruginosa*.

The ability to produce biofilm increases the resistance of microbial cells against harmful environments. Bacterial adhesion to the surface, as well as cell-cell interaction via biological membrane, occurs due to electrostatic interactions. In order to inhibit biofilm formation, one strategy is to break the adhesive forces between bacterial cells and the surface which can be achieved with the use of various antimicrobial agents^58^. In this aspect, the synthesised azo-phenol derivatives were tested for their hydrolysis potential against biofilm formation. Therefore, in the case of *P. aeruginosa* (DR), *P. aeruginosa* (DS); it was found that at the concentration of 0.5 mg/mL, all the derivatives except **TR**-**4** and **TR**-**7** (Figure 8(a)) were highly active against *P. aeruginosa* (DS) in biofilm inhibition (>70%) compared to the standard drug penicillin, which showed 60% inhibition at 0.5 mg/mL, however, against *P. aeruginosa* (DR) most of the azo derivatives were active (∼70%) in degrading biological membrane but **TR-5** and **TR-8** efficacy was found to be highest (80%) compared to the standard i.e. moxifloxacin (**8b**). Interestingly, the compound **TR**-**7** showed 55% anti-biofilm potency against the drug-resistant strain of *P. aeruginosa* which was not found active against *P. aeruginosa* (DS). However, the rest of the tested derivatives were found less efficient in this regard.

**Figure 9.**
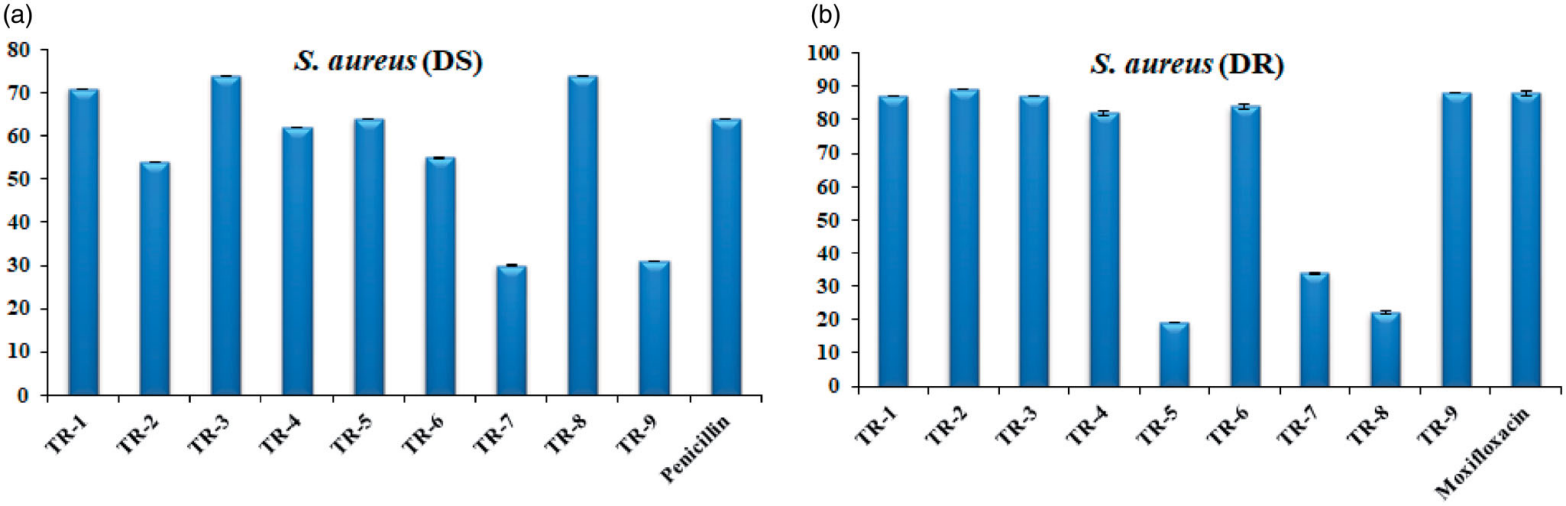
Biofilm hydrolysis potential of azo-phenol compounds against *S. aureus*.

Similarly, between *S. aureus* (DS) and *S. aureus* (DR) biofilm hydrolysis efficiencies, **TR-1, TR-3, TR-8** showed potential greater than 70% compared to a penicillin (65%) and all the derivatives except **TR-5, TR-7** and **TR-8** showed efficacy equal to moxifloxacin (85%) at 0.5 mg/mL concentration respectively. It was also observed that the efficiency of the compound **TR**-**8** was drastically decreased when it was challenged with the drug-resistant strain of *S. aureus* (Figure 9).

## 4. Conclusions

In summary, a series of azo derivatives were synthesised *via* the diazo-coupling approach between substituted aromatic amines with phenol derivatives. All the synthesised compounds were obtained in good to moderate yields and analytically determined by spectroscopic methods (IR, NMR and HRMS). To the best of our knowledge, the anti-diabetic and antimicrobial activities of the reported derivatives were not evaluated earlier. Hence, the title compounds were biologically screened *in vitro* against α-glucosidase and pathogenic bacterial strains i.e. *E. coli* (gram-negative), *S. aureus* (gram-positive), *S. aureus* (gram-positive) drug-resistant strain, *P. aeruginosa* (gram-negative), *P. aeruginosa* (gram-negative) drug-resistant strain and *P. vulgaris* (gram-negative). The experimental data showed that the compounds under consideration have promising pharmacological properties to be used as potential drug leads for the treatment against diabetes mellitus (DM). The title compounds can also serve as the functional template in the antimicrobial resistance (AMR) issue as they have been screened against broad-spectrum bacteria including drug-resistant strains and exhibited good to moderate antimicrobial potential as almost all the compounds exhibited dose-dependent broad-spectrum activity against screened pathogens (MIC 125 µg/200 µL) and biofilm hydrolysis potential (>60 − 80%). The presence of an aromatic hydroxyl group and a heterocyclic pharmacophore fused with the azo moiety confirmed their high inhibitory potency against the α-glucosidase enzyme. The enzyme inhibitory kinetics, mode of binding and docking studies of the most potent inhibitors could help out to design good inhibitors against α-glucosidase to overcome the worldwide health problem of diabetes.

## Supplementary Material

Supplemental Material

## References

[1] Chhetri A, Chettri S, Rai P, et al. Synthesis, characterization and computational study on potential inhibitory action of novel azo imidazole derivatives against COVID-19 main protease (Mpro: 6LU7). J Mol Struct 2021;1225:129230. 32963413 10.1016/j.molstruc.2020.129230 PMC7499073

[2] Niluvanji Matada M, Jathi K, Rangappa MM, et al. A new sulphur containing heterocycles having azo linkage: synthesis, structural characterization and biological evaluation. J King Saud Uni – Science 2020; 32:3313–20.

[3] Maliyappa M, Keshavayya J, Mallikarjuna N, Pushpavathi I. Novel substituted aniline based heterocyclic dispersed azo dyes coupling with 5-methyl-2-(6-methyl-1, 3-benzothiazol-2-yl)-2, 4-dihydro-3H-pyrazol-3-one: synthesis, structural, computational and biological studies. J Mol Struct 2020;1205:127576.

[4] Ravi BN, J K, M MN, et al. Synthesis, characterization and pharmacological evaluation of 2-aminothiazole incorporated azo dyes. J Mol Struct 2020;1204:127493.

[5] Debnath P, Das A, Singh KS, et al. Synthesis, structural characterization and antimicrobial activities of triorganotin (IV) azo-carboxylates derived from ortho/para-amino benzoic acids and β-naphthol. Inorganica Chimica Acta 2019;498:119172.

[6] Ispir E, Ikiz M, Inan A, et al. Synthesis, structural characterization, electrochemical, photoluminescence, antiproliferative and antioxidant properties of Co (II), Cu (II) and Zn (II) complexes bearing the azo-azomethine ligands. J Mol Struct 2019;1182:63–71.

[7] Matada MN, Jathi K. A novel azo metal complexes of 5, 5, 7-trimethyl-4, 5, 6, 7-tetrahydro-1, 3-benzothiazol as potential pharmacological agents: Synthesis and spectroscopic characterization. J Mol Struct 2019;1180:196–208.

[8] Tavaf Z, Dangolani SK, Yousefi R, et al. Synthesis of new curcumin derivatives as influential antidiabetic α-glucosidase and α-amylase inhibitors with anti-oxidant activity. Carbohydrate Res 2020;494:108069. 10.1016/j.carres.2020.108069 32563890

[9] Peytam F, Adib M, Shourgeshty R, et al. Synthesis and biological evaluation of new dihydroindolizino[8,7-b]indole derivatives as novel α-glucosidase inhibitors. J Mol Struct 2021;1224:129290.

[10] Azimi F, Ghasemi JB, Azizian H, et al. Design and synthesis of novel pyrazole-phenyl semicarbazone derivatives as potential α-glucosidase inhibitor: kinetics and molecular dynamics simulation study. Int J Biol Macromolecule 2021;166:1082–95. 10.1016/j.ijbiomac.2020.10.263 33157144

[11] Abdullah MA, Lee Y-R, Mastuki SN, et al. Development of diarylpentadienone analogues as alpha-glucosidase inhibitor: synthesis, *in vitro* biological and in vivo toxicity evaluations, and molecular docking analysis. Bioorg Chem 2020;104:104277. 32971414 10.1016/j.bioorg.2020.104277

[12] Akhter S, Ullah S, Yousuf S, et al. Synthesis, crystal structure and Hirshfeld Surface analysis of benzamide derivatives of thiourea as potent inhibitors of α-glucosidase *in-vitro*. Bioorg Chem 2021;107:104531. 33339666 10.1016/j.bioorg.2020.104531

[13] Aziz H, Saeed A, Rehman AU, et al. Synthesis, characterization, in vitro biological and computational evaluation of 5-benzyl-4-(benzylideneamino)-2*H*-1, 2, 4-triazole-3 (4*H*)-thiones. J Iranian Chem Soc 2021;1–13. DOI:10.1007/s13738-021-02156-5

[14] Yang J, Wang X, Zhang C, et al. Comparative study of inhibition mechanisms of structurally different flavonoid compounds on α-glucosidase and synergistic effect with acarbose. Food Chem 2021;347:129056. 33476922 10.1016/j.foodchem.2021.129056

[15] Yousuf H, Shamim S, Khan KM, et al. Dihydropyridines as potential α-amylase and α-glucosidase inhibitors: synthesis, *in vitro* and *in silico* studies. Bioorg Chem 2020;96:103581. 31978686 10.1016/j.bioorg.2020.103581

[16] Ali G, Cuny GD. An efficient synthesis of an 8-phenoxy aporphine derivative utilizing mono-ligated palladium ortho-phenol arylation. Tetrahedron 2019;75:4318–24.

[17] Rai S, Kureel AK, Dutta PK, Mehrotra GK. Phenolic compounds based conjugates from dextran aldehyde and BSA: preparation, characterization and evaluation of their anti-cancer efficacy for therapeutic applications. Int J Biol Macromolecule 2018;110:425–36. 10.1016/j.ijbiomac.2017.11.049 29129629

[18] Al Majidi MIH, El-Shaheny R, El-Shabrawy Y, El-Maghrabey M. Screening and greenness profiling of oxidative-coupling and electrophilic aromatic substitution reactions for determination of three phenolic drugs. Microchem J 2019;149:104051.

[19] Okasha RM, Alsehli M, Ihmaid S, et al. First example of Azo-Sulfa conjugated chromene moieties: synthesis, characterization, antimicrobial assessment, docking simulation as potent class I histone deacetylase inhibitors and antitumor agents. Bioorg Chem 2019;92:103262. 31518757 10.1016/j.bioorg.2019.103262

[20] Obodoechi LO, Carvalho I, Chenouf NS, et al. Antimicrobial resistance in *Escherichia coli* isolates from frugivorous (*Eidolon helvum*) and insectivorous (*Nycteris hispida*) bats in Southeast Nigeria, with detection of CTX-M-15 producing isolates. Comparative Immunol Microbiol Infect Disease 2021;75:101613. 10.1016/j.cimid.2021.101613 33465673

[21] Ben Mohamed-Smati S, Faraj FL, Becheker I, et al. Synthesis, characterization and antimicrobial activity of some new azo dyes derived from 4-hydroxy-6-methyl-2H-pyran-2-one and its dihydro derivative. Dye Pigment 2021;188:109073.

[22] Ribeiro D, Poença C, Varela C, et al. New phenolic cinnamic acid derivatives as selective COX-2 inhibitors. Design, synthesis, biological activity and structure-activity relationships. Bioorg Chem 2019; 91:103179. 31404794 10.1016/j.bioorg.2019.103179

[23] Krátký M, Janďourek O, Baranyai Z, et al. Phenolic N-monosubstituted carbamates: antitubercular and toxicity evaluation of multi-targeting compounds. Europ J Med Chem 2019; 181:111578. 10.1016/j.ejmech.2019.111578 31401536

[24] Dayma V, Chopra J, Sharma P, et al. Synthesis, antidiabetic, antioxidant and anti-inflammatory activities of novel hydroxytriazenes based on sulpha drugs. Heliyon 2020;6:e04787. 32913908 10.1016/j.heliyon.2020.e04787 PMC7472862

[25] Prakash S, Somiya G, Elavarasan N, et al. Synthesis and characterization of novel bioactive azo compounds fused with benzothiazole and their versatile biological applications. J Mol Struct 2021;1224:129016.

[26] Yu J, Xu L, Hong D, et al. Design, synthesis, and biological evaluation of novel phenol ether derivatives as non-covalent proteasome inhibitors. Europ J Med Chem 2019;161:543–58. 10.1016/j.ejmech.2018.10.056 30391816

[27] Athira L, Balachandran S, Devi RS. Synthesis, crystal structure, solvatochromic properties and DNA cleaving activity of azo derivative of naphthalen-2-ol. J Mol Struct 2019;1180:100–9.

[28] Blümel S, Stolz A. Cloning and characterization of the gene coding for the aerobic azoreductase from Pigmentiphaga kullae K24. Applied Microbiol Biotechnol 2003;62:186–90. 10.1007/s00253-003-1316-5 12719939

[29] Valizadeh H, Amiri M, Shomali A, Hosseinzadeh F. Ionic liquid 1-(3-Trimethoxysilylpropyl)-3-methylimidazolium nitrite as a new reagent for the efficient diazotization of aniline derivatives and *in situ* synthesis of azo dyes. J Iran Chem Soc 2011;8:495–501.

[30] Zellner H. 1-(2-Nitro-4-methylphenylazo)-2-naphthol. Fresenius’ Zeitschrift Für Analytische Chemie 1953;140:317.

[31] Arshad T, Khan KM, Rasool N, et al. Syntheses, *in vitro* evaluation and molecular docking studies of 5-bromo-2-aryl benzimidazoles as α-glucosidase inhibitors. Med Chem Res 2016;25:2058–69.

[32] Ur Rehman N, Halim SA, Al-Azri M, et al. Triterpenic acids as non-competitive α-glucosidase inhibitors from *Boswellia elongata* with structure-activity relationship: *in vitro* and *in silico* studies. Biomolecule 2020;10:751. 10.3390/biom10050751 PMC7278020 32408614

[33] Motoshima K, Noguchi-Yachide T, Sugita K, et al. Separation of α-glucosidase-inhibitory and liver X receptor-antagonistic activities of phenethylphenyl phthalimide analogs and generation of LXRα-selective antagonists. Bioorg Med Chem 2009;17:5001–14. 19539483 10.1016/j.bmc.2009.05.066

[34] Pettersen EF, Goddard TD, Huang CC, Couch GS, et al. UCSF Chimera—a visualization system for exploratory research and analysis. J Computation Chem 2004;25:1605–12. 10.1002/jcc.20084 15264254

[35] Lovell SC, Davis IW, Arendall IIW, et al. Structure validation by Cα geometry: ϕ, ψ and Cβ deviation. Proteins: Struct Function Bioinfo 2003;50:437–50. 10.1002/prot.10286 12557186

[36] Chen VB, Arendall WB, Headd JJ, et al. MolProbity: all-atom structure validation for macromolecular crystallography. Acta Crystallographica Section D: Biol Crystallograph 2010;66:12–21. 10.1107/S0907444909042073 PMC2803126 20057044

[37] Monzavi H, Willard L, Zhang H, et al. VADAR: a web server for quantitative evaluation of protein structure quality. 2003. 10.1093/nar/gkg565 PMC168972 12824316

[38] Dallakyan S, Olson AJ, Small-molecule library screening by docking with PyRx. Chem Biol. Springer; 2015: 243–50. DOI:10.1007/978-1-4939-2269-7_19 25618350

[39] Famuyide IM, Aro AO, Fasina FO, et al. Antibacterial and antibiofilm activity of acetone leaf extracts of nine under-investigated South African Eugenia and Syzygium (Myrtaceae) species and their selectivity indices. BMC Complement Alternative Med 2019;19:141. 10.1186/s12906-019-2547-z PMC6587284 31221162

[40] Brahma U, Kothari R, Sharma P, Bhandari V. Antimicrobial and anti-biofilm activity of hexadentated macrocyclic complex of copper (II) derived from thiosemicarbazide against *Staphylococcus aureus*. Sci Rep 2018;8:1–8. 29795120 10.1038/s41598-018-26483-5 PMC5966380

[41] Kaur H, Lim SM, Ramasamy K, et al. Diazenyl schiff bases: synthesis, spectral analysis, antimicrobial studies and cytotoxic activity on human colorectal carcinoma cell line (HCT-116). Arab J Chem 2020;13:377–92.

[42] Aziz H, Saeed A, Khan MA, et al. Synthesis, characterization, antimicrobial, antioxidant and computational evaluation of N-acyl-morpholine-4-carbothioamides. Mol Diversity 2020;25:1–14. 10.1007/s11030-020-10054-w 32100245

[43] Aziz H, Saeed A, Khan MA, et al. Novel N‐Acyl‐1H‐imidazole‐1‐carbothioamides: design, synthesis, biological and computational studies. Chem Biodiversity 2020;17:e1900509. 10.1002/cbdv.201900509 31943755

[44] Shukla SK, Rao TS. An improved crystal violet assay for biofilm quantification in 96-well microtitre plate. BioRxiv 2017;100214. DOI:10.1101/100214

[45] Xu H, Zeng X. Synthesis of diaryl-azo derivatives as potential antifungal agents. Bioorg Med Chem Lett 2010;20:4193–5. 20570508 10.1016/j.bmcl.2010.05.048

[46] Qiu J, Xiao J, Tang B, et al. Facile synthesis of novel disperse azo dyes with aromatic hydroxyl group. Dye Pigment 2019;160:524–9.

[47] Maliyappa M, Keshavayya J, Mallikarjuna N, et al. Synthesis, characterization, pharmacological and computational studies of 4, 5, 6, 7-tetrahydro-1, 3-benzothiazole incorporated azo dyes. J Mol Struc 2019;1179:630–41.

[48] Ghanavatkar CW, Mishra VR, Mali SN, et al. Synthesis, bioactivities, DFT and in-silico appraisal of azo clubbed benzothiazole derivatives. J Mol Struct 2019;1192:162–71.

[49] Kantar C, Akal H, Kaya B, et al. Novel phthalocyanines containing resorcinol azo dyes; synthesis, determination of pKa values, antioxidant, antibacterial and anticancer activity. J Organometal Chem 2015;783:28–39.

[50] Keshavayya J. Synthesis, structural investigations and in vitro biological evaluation of N, N-dimethyl aniline derivatives based azo dyes as potential pharmacological agents. J MolStruct 2019;1186:404–12.

[51] Delogu GL, Era B, Floris S, et al. A new biological prospective for the 2-phenylbenzofurans as inhibitors of α-glucosidase and of the islet amyloid polypeptide formation. Int J Biol Macromol 2021;169:428–35. 33347933 10.1016/j.ijbiomac.2020.12.117

[52] Jenis J, Baiseitova A, Yoon SH, et al. Competitive α-glucosidase inhibitors, dihydrobenzoxanthones, from the barks of *Artocarpus elasticus*. J Enzyme Inhibition Med Chem 2019;34:1623–32. 10.1080/14756366.2019.1660653 PMC6735331 31480857

[53] Hassan M, Abbas Q, Ashraf Z, et al. Pharmacoinformatics exploration of polyphenol oxidases leading to novel inhibitors by virtual screening and molecular dynamic simulation study. Computation Biol Chem 2017;68:131–42. 10.1016/j.compbiolchem.2017.02.012 28340400

[54] Hassan M, Ashraf Z, Abbas Q, et al. Exploration of novel human tyrosinase inhibitors by molecular modeling, docking and simulation studies. Interdisciplinary Sci: Computation Life Sci 2018;10:68–80. 10.1007/s12539-016-0171-x 27098808

[55] Hassan M, Shahzadi S, Seo SY, et al. Molecular docking and dynamic simulation of AZD3293 and solanezumab effects against BACE1 to treat Alzheimer’s disease. Frontier Computation Neurosci 2018;12:34. 10.3389/fncom.2018.00034 PMC5992503 29910719

[56] Channar PA, Saeed A, Albericio F, et al. Sulfonamide-linked ciprofloxacin, sulfadiazine and amantadine derivatives as a novel class of inhibitors of jack bean urease; synthesis, kinetic mechanism and molecular docking. Molecule 2017;22:1352. 10.3390/molecules22081352 PMC6152116 28813027

[57] Abbas Q, Hassan M, Raza H, et al. *In vitro*, *in vivo* and *in silico* anti-hyperglycemic inhibition by sinigrin. Asian Pac J Trop Med 2017;10:372–9. 28552107 10.1016/j.apjtm.2017.03.019

[58] Sai Saraswathi V, Kamarudheen N, Bhaskara Rao KV, Santhakumar K. Biofilm inhibition formation of clinical strains of *Pseudomonas aeruginosa* mutans, photocatalytic activity of azo dye and GC–MS analysis of leaves of *Lagerstroemia speciosa*. J Photochem Photobiol B: Biol 2017;169:148–60. 10.1016/j.jphotobiol.2017.03.007 28319869

