# Supplementary material for: Diaryl azo derivatives as anti-diabetic and antimicrobial agents: synthesis, in vitro, kinetic and docking studies
Source: J Enzyme Inhib Med Chem. 2021 Jul 8;36(1):1509–20. doi: 10.1080/14756366.2021.1929949 (PMC8274517; doi:10.1080/14756366.2021.1929949)
Supplement: Supplemental Material [file IENZ_A_1929949_SM1797.pdf]

Diaryl azo derivatives as anti-diabetic and antimicrobial agents: Synthesis, *in vitro*, kinetic and docking Studies

Tehreem Tahir [1], Mirza Imran Shahzad [1,*], Rukhsana Tabassum [2], Muhammad Rafiq [3], Muhammad Ashfaq [2], Mubashir Hassan [4], Katarzyna Kotwica-Mojzych[4], Mariusz Mojzych [5,*]

[1] Institute of Biochemistry, Biotechnology and Bioinformatics, Faculty of Science, The Islamia University of Bahawalpur, Bahawalpur 63100, Pakistan;

[2] Department of Chemistry, Faculty of Science, The Islamia University of Bahawalpur, Bahawalpur 63100, Pakistan;

[3] Department of Physiology and Biochemistry, Faculty of Bio-Sciences, Cholistan University of Veterinary and Animal Sciences, Bahawalpur 63100, Pakistan;

[4] Institute of Molecular Biology & Biotechnology, The University of Lahore (Defense Road Campus), Lahore, Pakistan;

[5] Department of Histology, Embryology and Cytophysiology, Medical University of Lublin, Radziwiłłowska 11, 20-080 Lublin, Poland;

[6] Department of Chemistry, Siedlce University of Natural Sciences and Humanities, 3-Maja 54, 08-110 Siedlce, Poland;

*Correspondence: mmojzych@yahoo.com (MM), mirza.imran@iub.edu.pk (TT)

Compound: **TR-1**

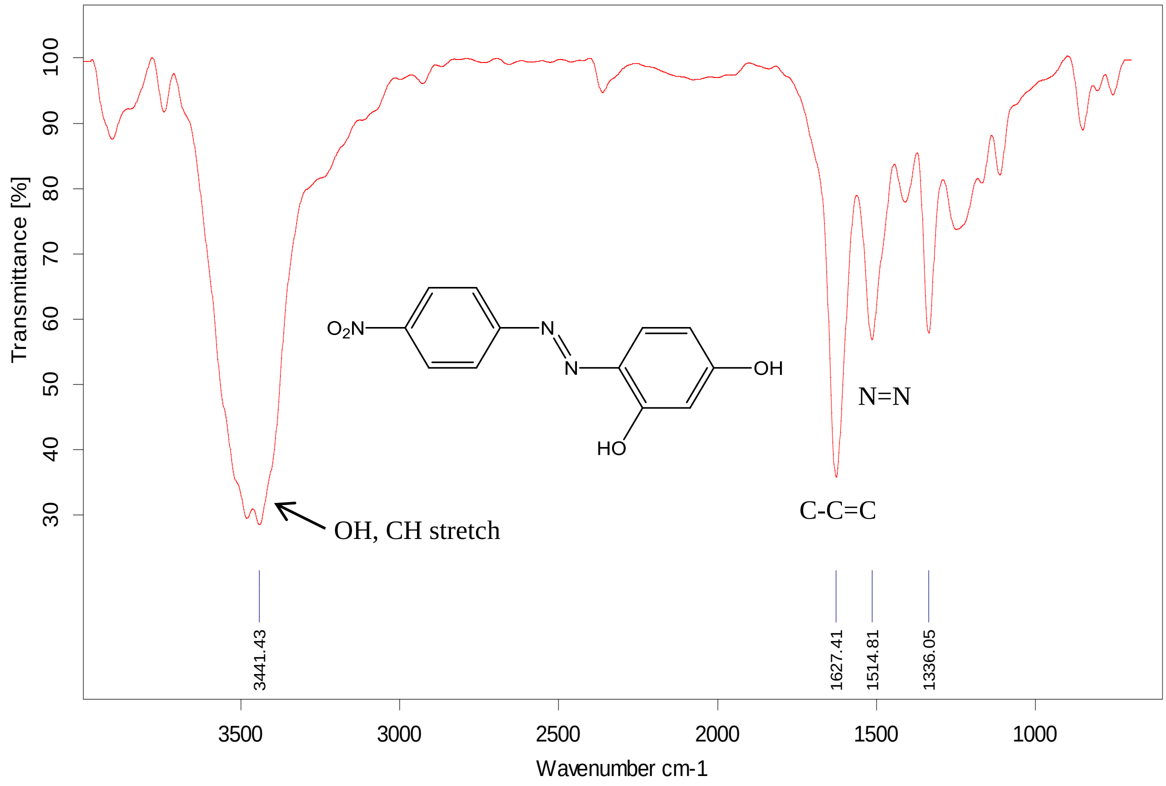

TC: 9	$C_{12}H_9N_3O_4$	259.2	IS	PS	IS	IS	IS	S	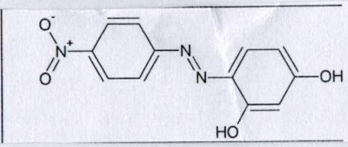

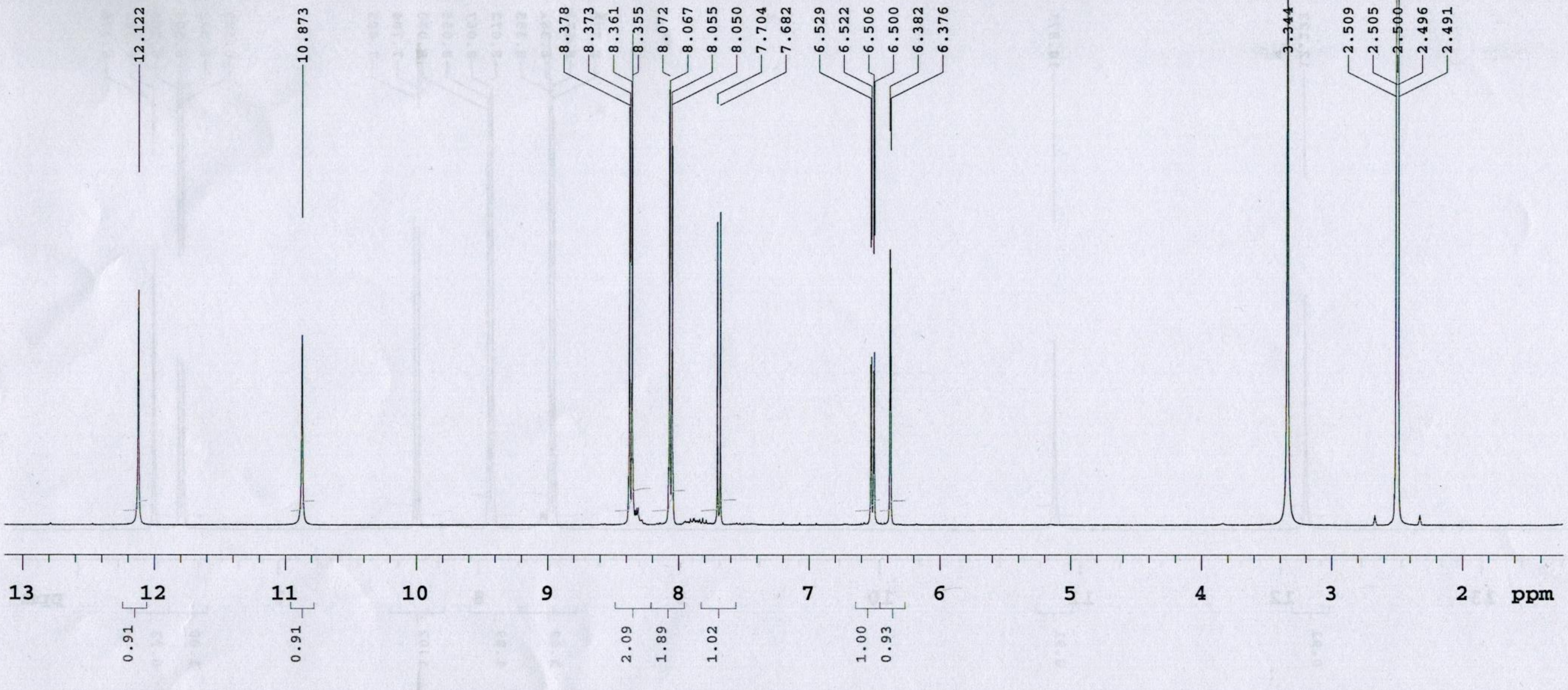

PULSE SEQUENCE
Relax. delay 0.500 sec
Pulse 48.6 degrees
Acq. time 4.797 sec
Width 7225.4 Hz
24 repetitions

OBSERVE H1, 399.5130588

DATA PROCESSING
FT size 65536
Total time 2 minutes

MMTC9a
in DMSO

Sample Name:
MMTC9a
Data Collected on:
400MR-vnmrs400
Archive directory: [illegible]

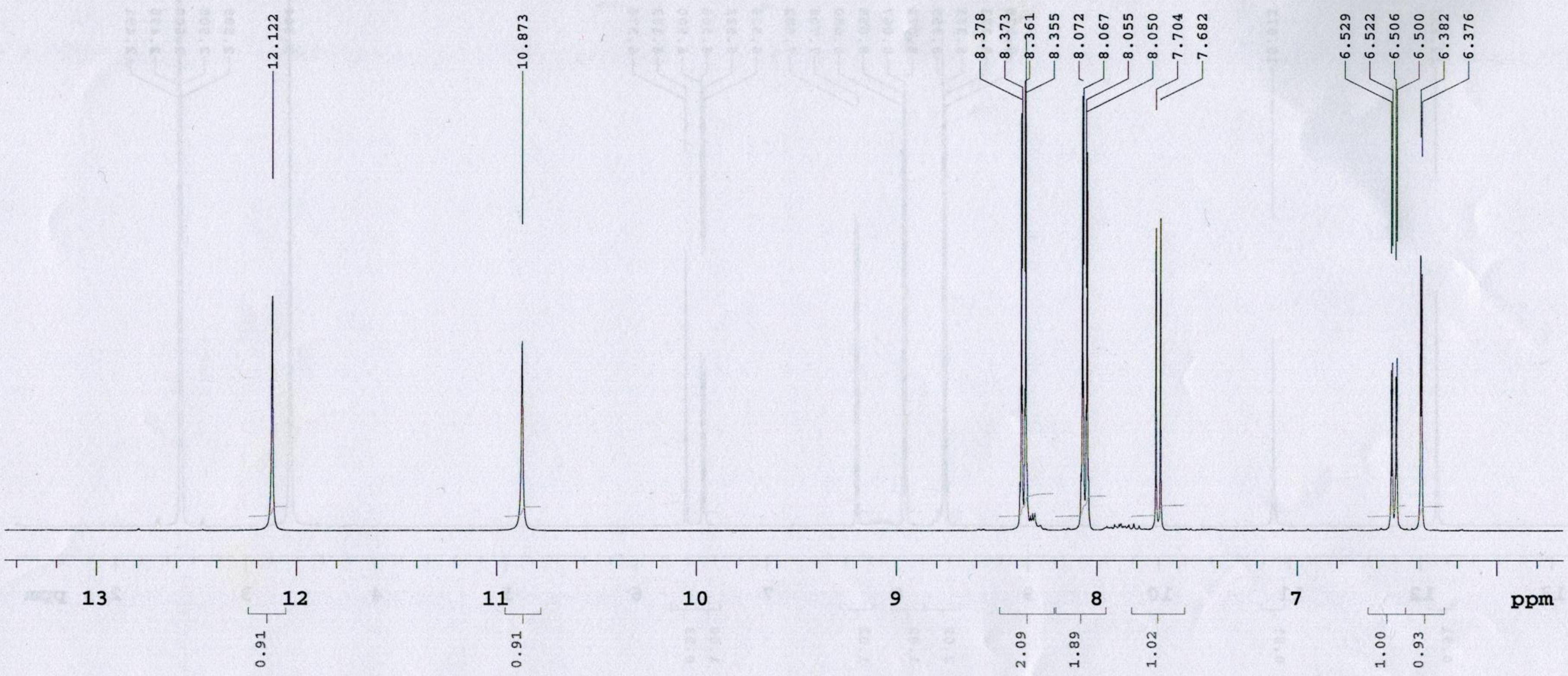

PULSE SEQUENCE
Relax. delay 0.500 sec
Pulse 48.6 degrees
Acq. time 4.797 sec
Width 7225.4 Hz
16 repetitions

OBSERVE H1, 399.5130588

DATA PROCESSING
FT size 65536
Total time 1 minute

MMTC9a
in DMSO

Sample Name:
MMTC9a
Data Collected on:
400MR-vnmrs400
Archive directory: [illegible]

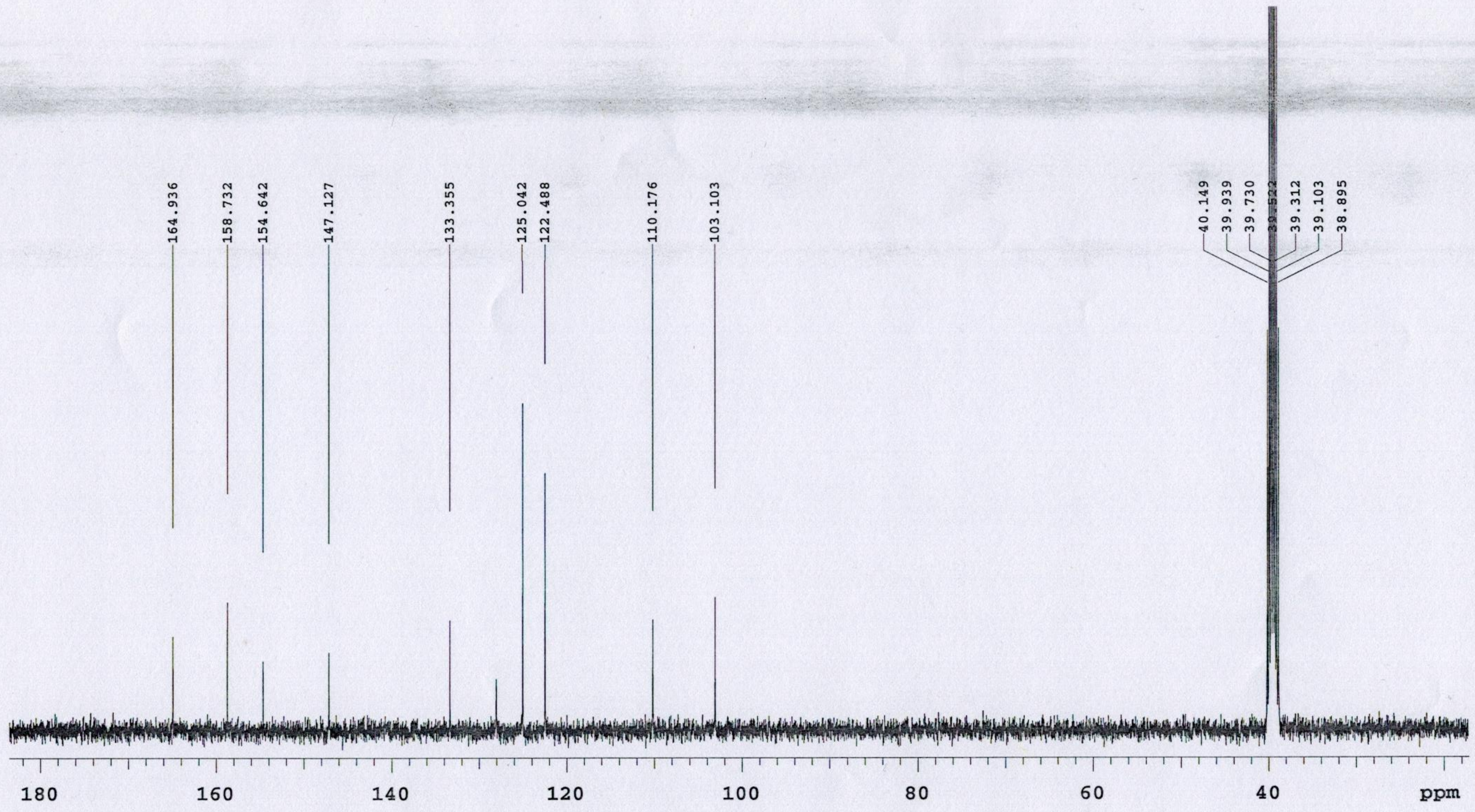

PULSE SEQUENCE
Relax. delay 1.500 sec
Pulse 38.5 degrees
Acq. time 2.674 sec
Width 24509.8 Hz
1456 repetitions

OBSERVE C13, 100.4576830
DECOUPLE H1, 399.5150667
Power 37 dB
continuously on
WALTZ-16 modulated

DATA PROCESSING
Line broadening 1.0 Hz
FT size 262144
Total time 101 minutes

MMTC9a
in DMSO

Sample Name:
MMTC9a
Data Collected on:
400MR-vnmrs400
[illegible]

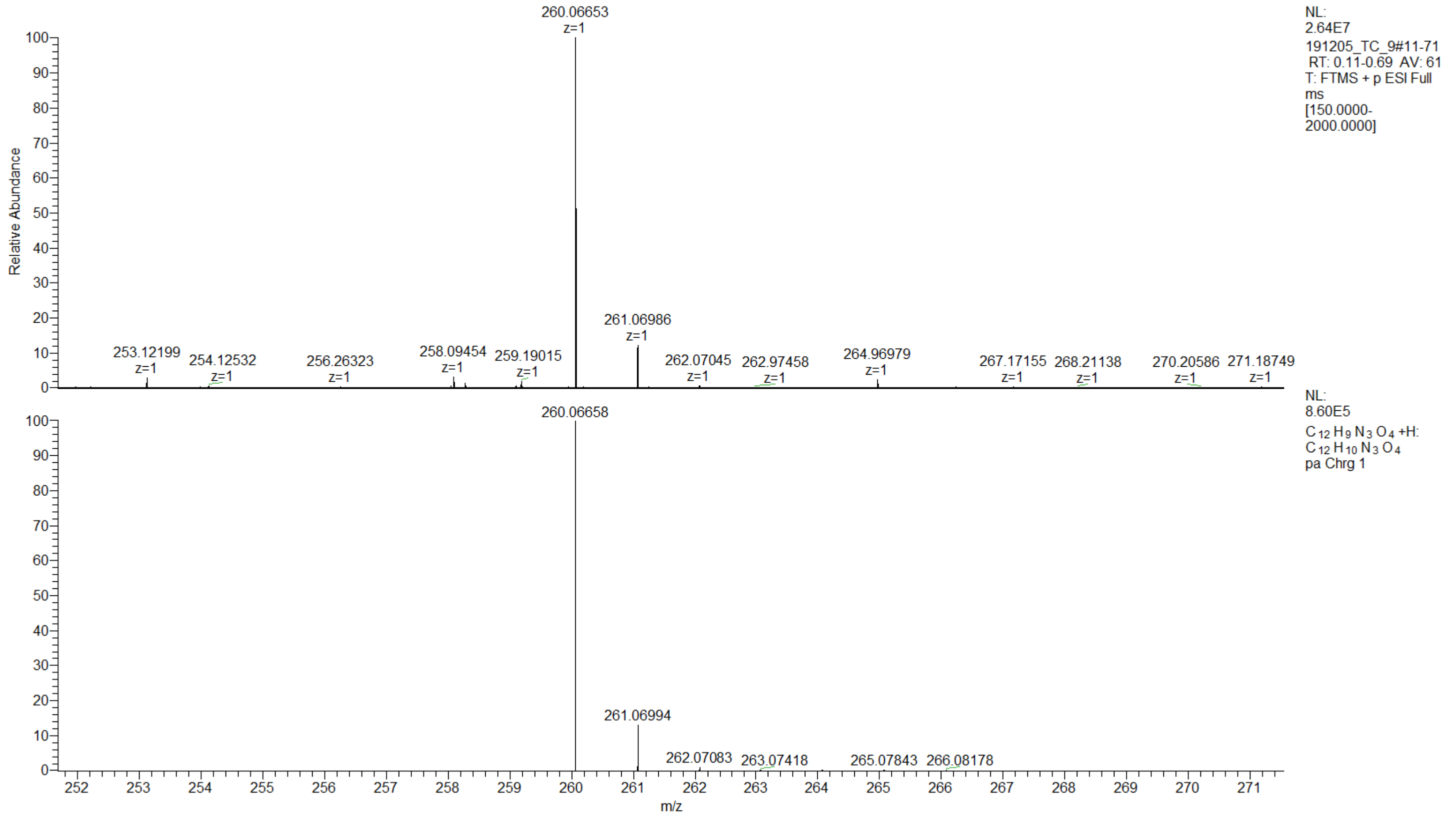

NL:
2.64E7
191205_TC_9#11-71
RT: 0.11-0.69 AV: 61
T: FTMS + p ESI Full ms [150.0000-2000.0000]
260.06653 z=1
261.06986 z=1
253.12199 z=1
254.12532 z=1
256.26323 z=1
258.09454 z=1
259.19015 z=1
262.07045 z=1
262.97458 z=1
264.96979 z=1
267.17155 z=1
268.21138 z=1
270.20586 z=1
271.18749 z=1
Relative Abundance
NL:
8.60E5
C 12 H 9 N 3 O 4 +H:
C 12 H 10 N 3 O 4
pa Chrg 1
260.06658
261.06994
262.07083
263.07418
265.07843
266.08178
m/z

Compound **TR-2**

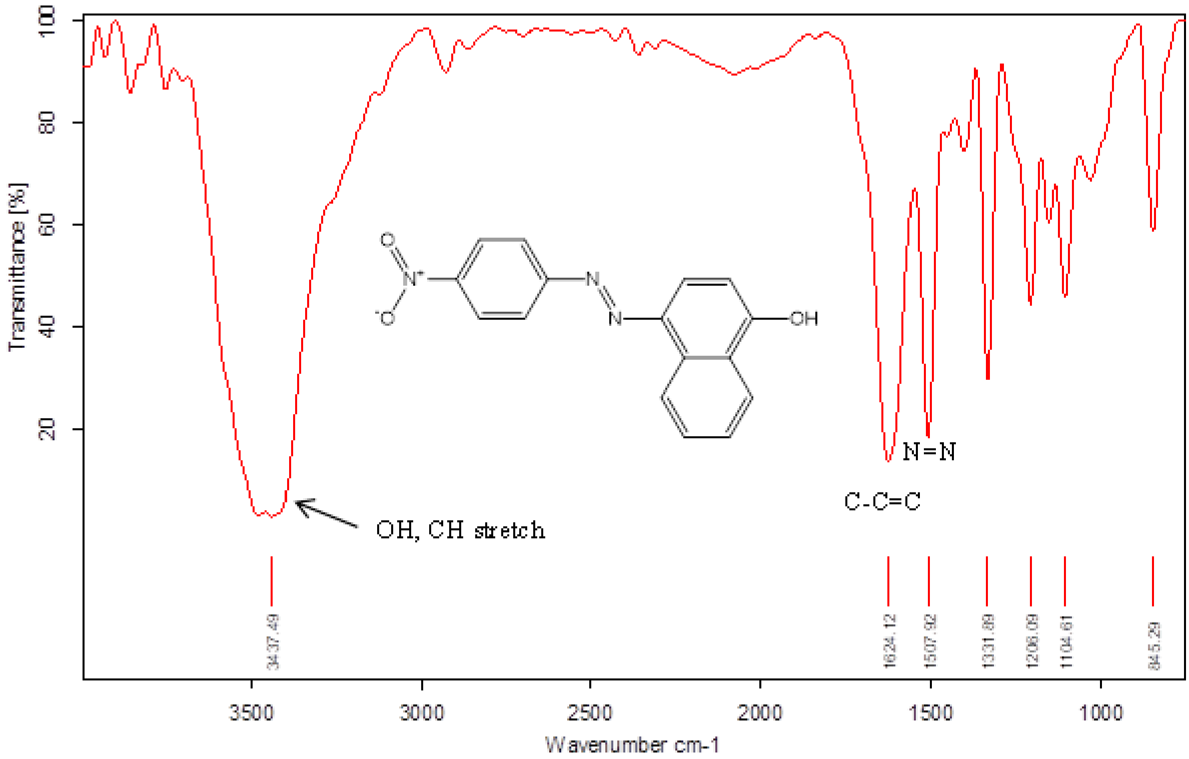

TC: 10	$C_{16}H_{11}N_3O_3$	293.2	IS	PS	IS	IS	IS	S	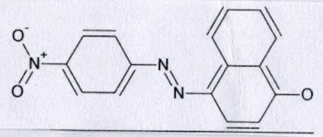

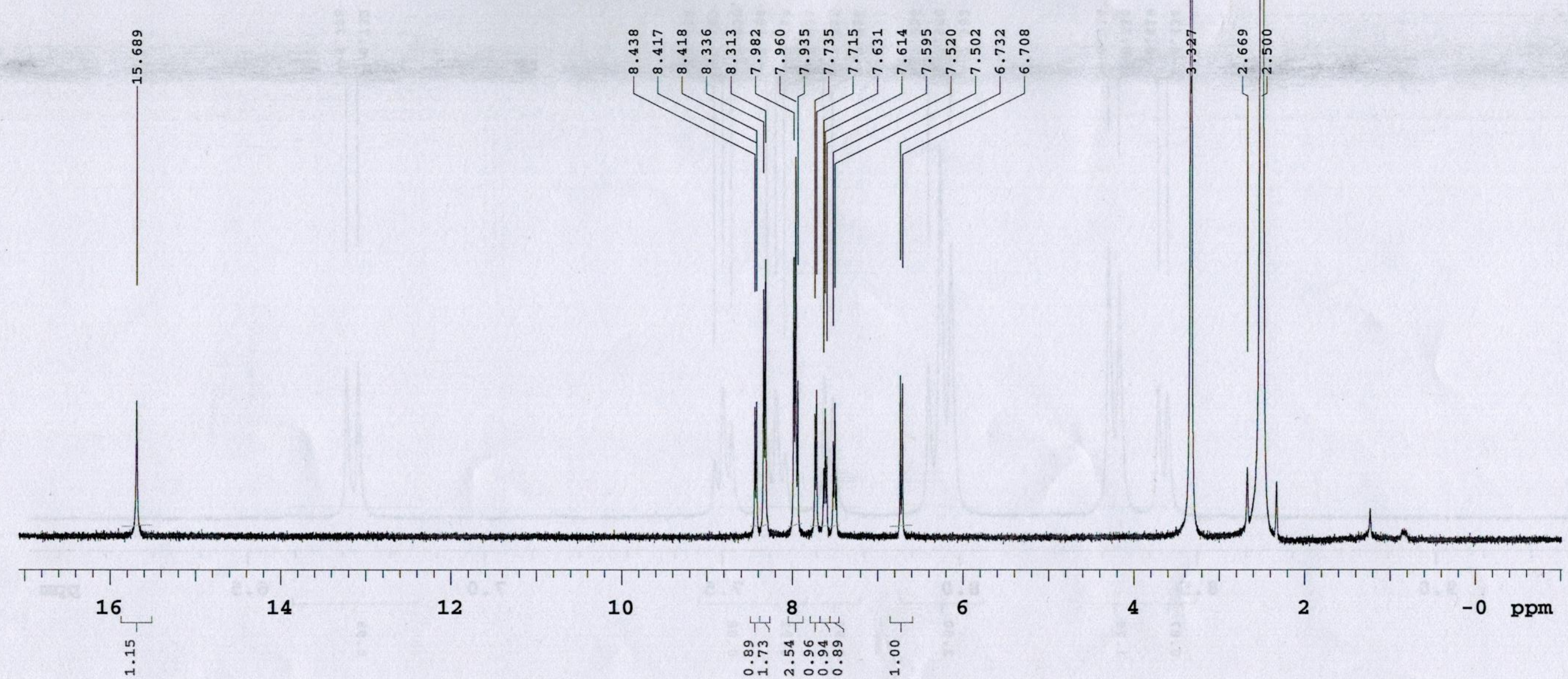

PULSE SEQUENCE
Relax. delay 0.500 sec
Pulse 48.6 degrees
Acq. time 4.797 sec
Width 7225.4 Hz
28 repetitions

OBSERVE H1, 399.5130588

DATA PROCESSING
FT size 65536
Total time 2 minutes

MMTC10a
in DMSO

Sample Name:
MMTC10a
Data Collected on:
400MR-vnmrs400
Archive directory:
[illegible]

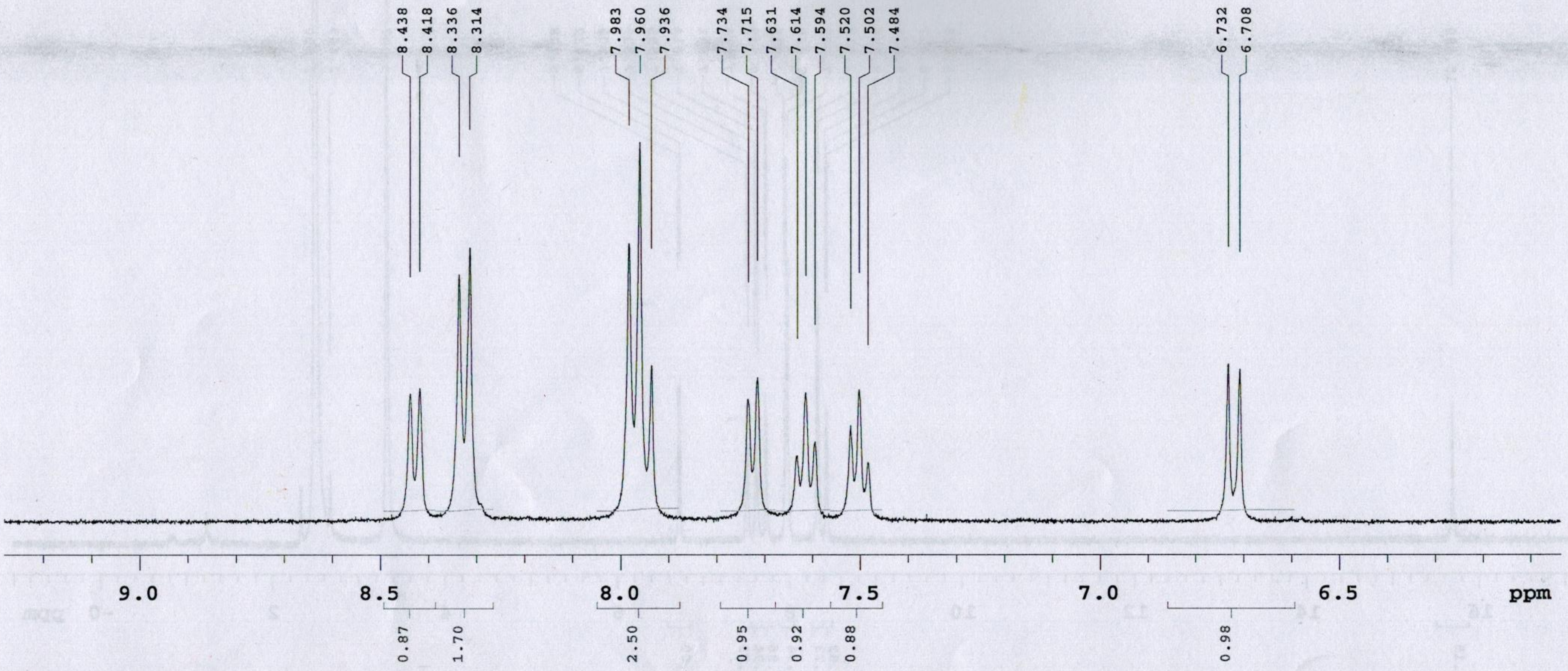

PULSE SEQUENCE
Relax. delay 0.500 sec
Pulse 48.6 degrees
Acq. time 4.797 sec
Width 7225.4 Hz
36 repetitions

OBSERVE H1, 399.5130588

DATA PROCESSING
FT size 65536
Total time 3 minutes

MMTC10a
in DMSO

Sample Name:
MMTC10a
Data Collected on:
400MR-vnmrs400
Archive directory: [illegible]

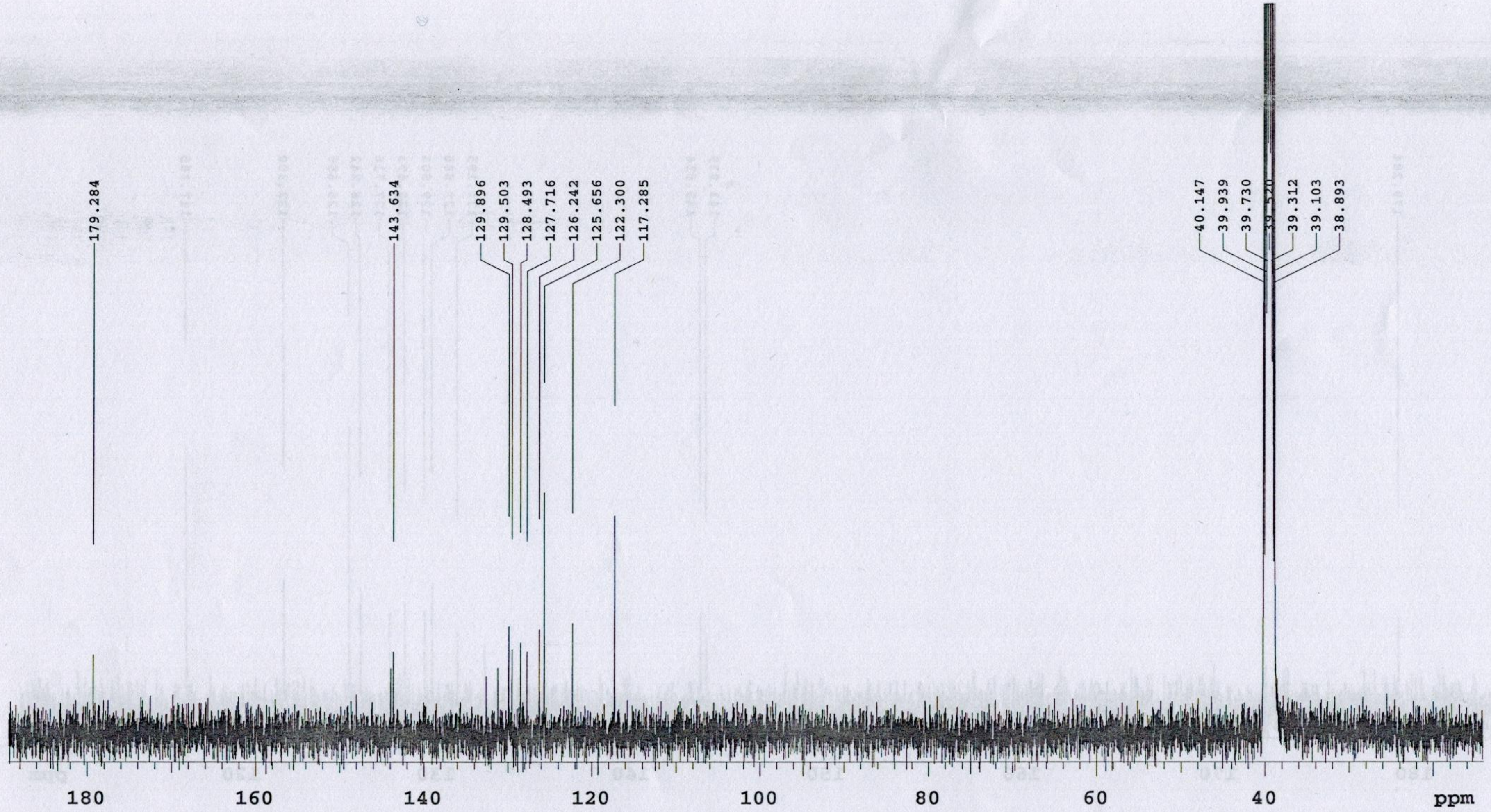

PULSE SEQUENCE	OBSERVE C13, 100.4576842	DATA PROCESSING	MMTC10a
Relax. delay 1.500 sec	DECOUPLE H1, 399.5150667	Line broadening 1.0 Hz	in DMSO
Pulse 38.5 degrees	Power 37 dB	FT size 262144	
Acq. time 2.674 sec	continuously on	Total time 11.6 hours	Sample Name:
Width 24509.8 Hz	WALTZ-16 modulated		MMTC10a
10000 repetitions			Data Collected on:
			400MR-vnmrs400
			Archive directory: [illegible]

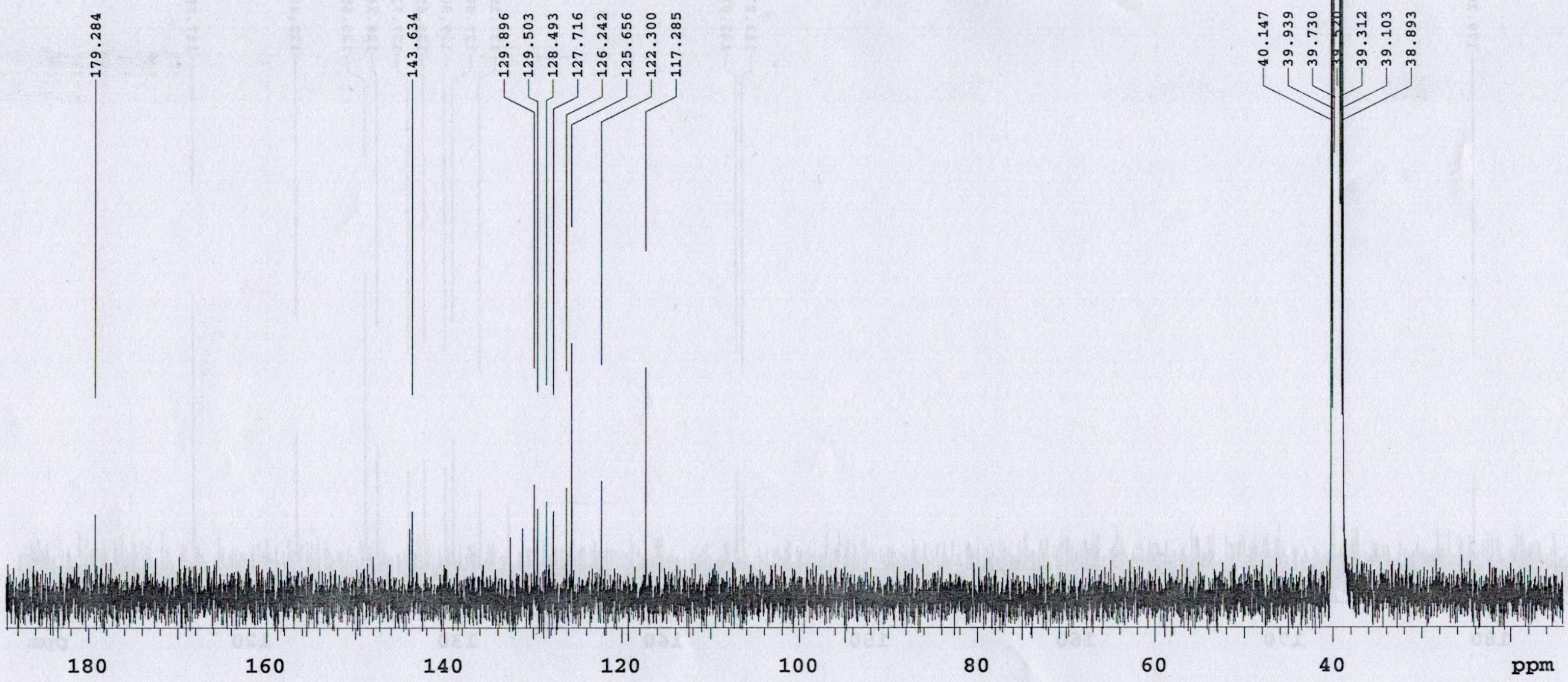

PULSE SEQUENCE
Relax. delay 1.500 sec
Pulse 38.5 degrees
Acq. time 2.674 sec
Width 24509.8 Hz
10000 repetitions

OBSERVE C13, 100.4576842
DECOUPLE H1, 399.5150667
Power 37 dB
continuously on
WALTZ-16 modulated

DATA PROCESSING
Line broadening 1.0 Hz
FT size 262144
Total time 11.6 hours

MMTC10a
in DMSO

Sample Name:
MMTC10a
Data Collected on:
400MR-vnmrs400
[illegible]

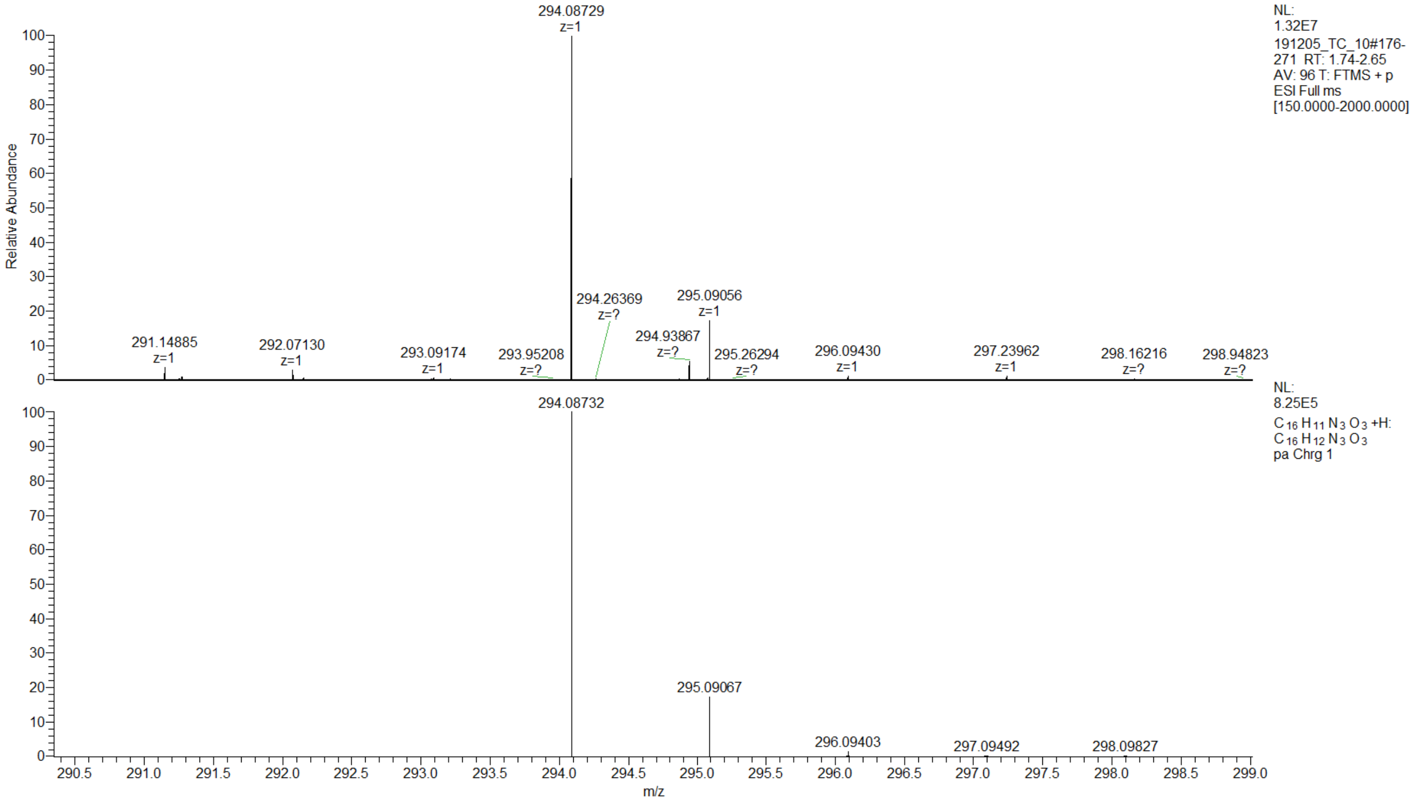

NL:
1.32E7
191205_TC_10#176-271 RT: 1.74-2.65
AV: 96 T: FTMS + p ESI Full ms [150.0000-2000.0000]
294.08729 z=1
294.26369 z=?
295.09056 z=1
294.93867 z=?
291.14885 z=1
292.07130 z=1
293.09174 z=1
293.95208 z=?
295.26294 z=?
296.09430 z=1
297.23962 z=1
298.16216 z=?
298.94823 z=?
NL:
8.25E5
C16 H11 N3 O3 +H: C16 H12 N3 O3
pa Chrg 1
294.08732
295.09067
296.09403
297.09492
298.09827
Relative Abundance
m/z

Compound **TR-3**

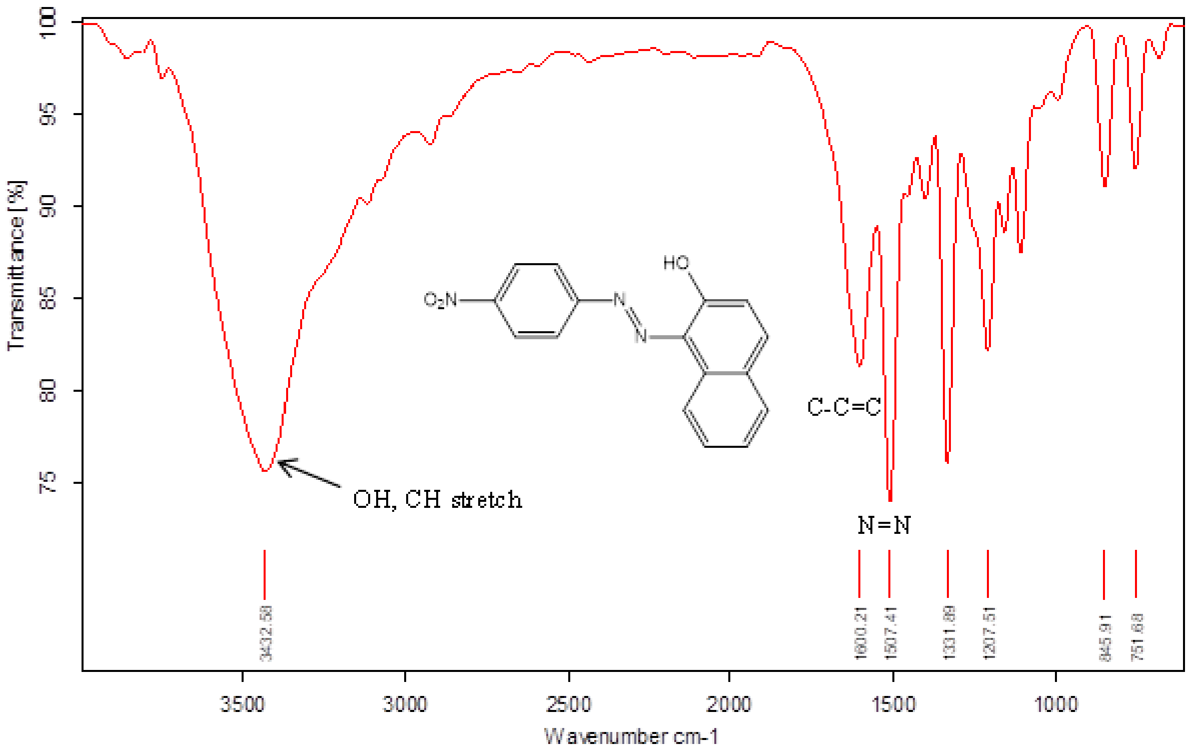

TC: 11	$C_{16}H_{11}N_3O_3$	293.2	IS	PS	IS	IS	IS	S	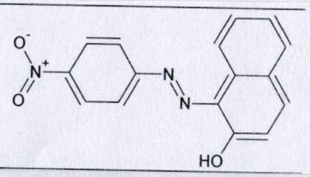

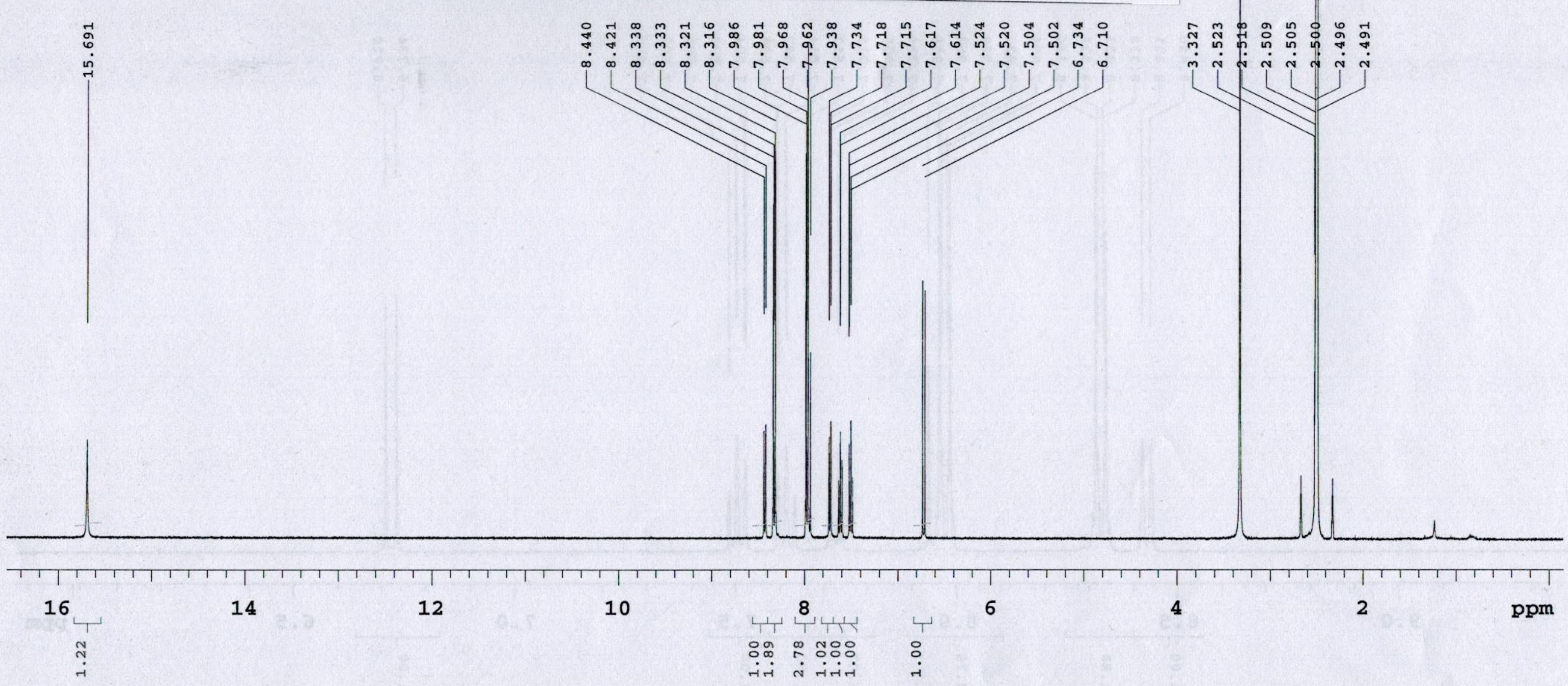

PULSE SEQUENCE
Relax. delay 0.500 sec
Pulse 48.6 degrees
Acq. time 4.797 sec
Width 7225.4 Hz
40 repetitions

OBSERVE H1, 399.5130588

DATA PROCESSING
FT size 65536
Total time 3 minutes

MMTC11a
in DMSO

Sample Name:
MMTC11a
Data Collected on:
400MR-vnmrs400
[illegible]

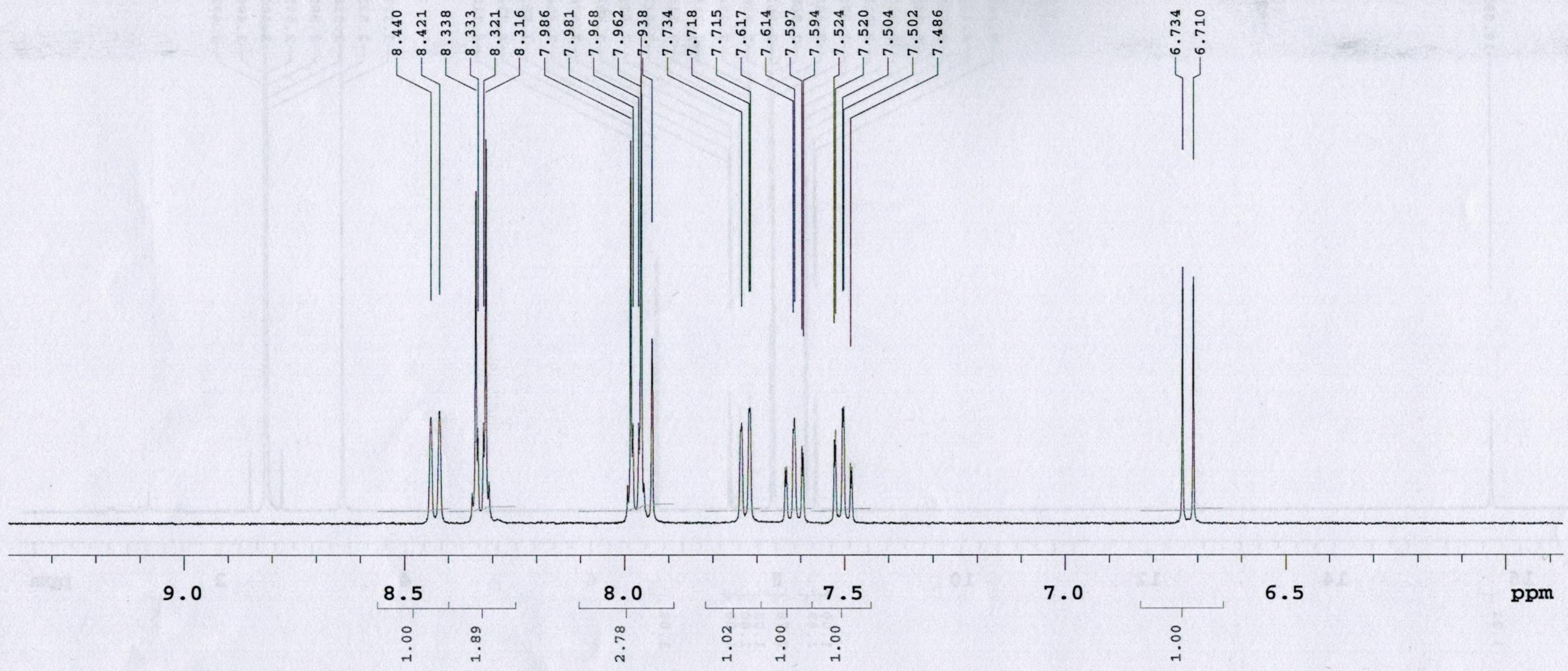

PULSE SEQUENCE
Relax. delay 0.500 sec
Pulse 48.6 degrees
Acq. time 4.797 sec
Width 7225.4 Hz
40 repetitions

OBSERVE H1, 399.5130588

DATA PROCESSING
FT size 65536
Total time 3 minutes

MMTC11a
in DMSO

Sample Name:
MMTC11a
Data Collected on:
400MR-vnmrs400
[illegible]

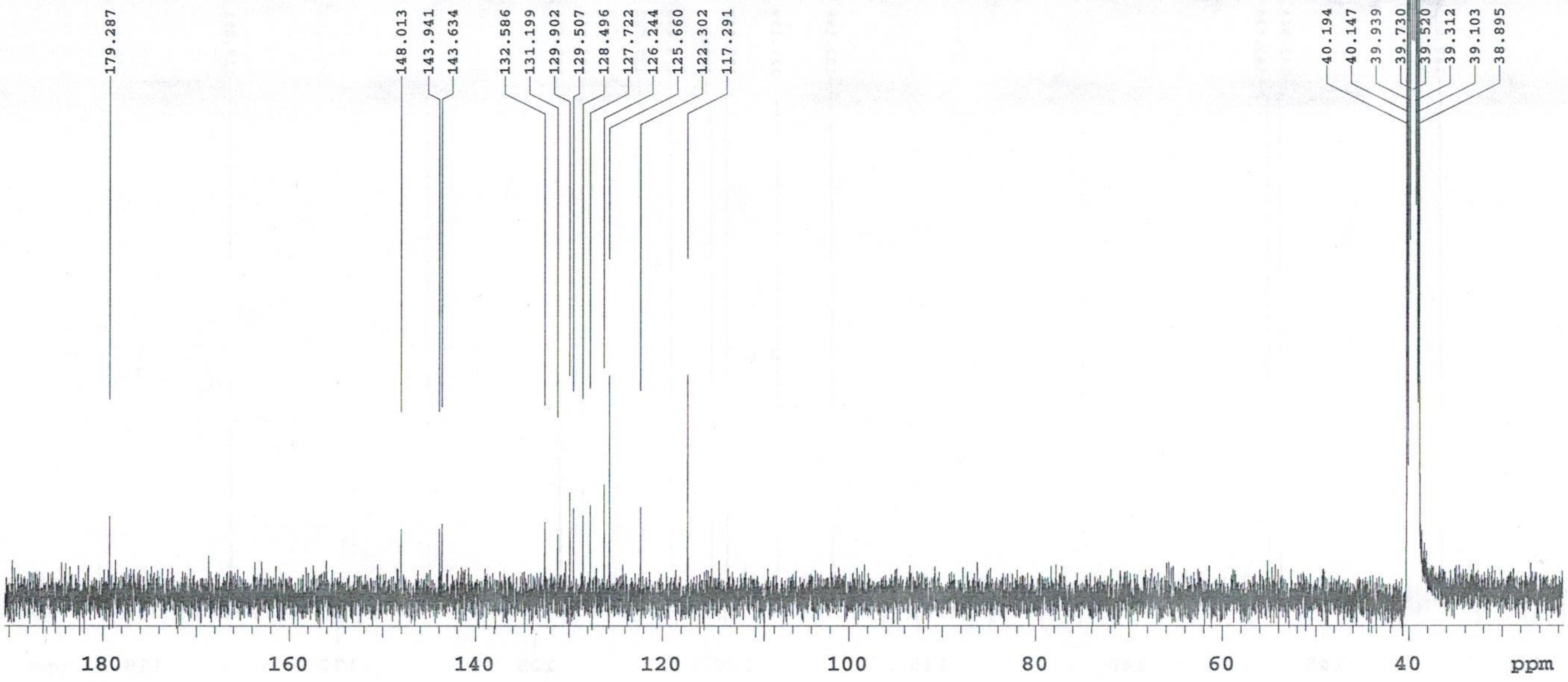

PULSE SEQUENCE
Relax. delay 1.500 sec
Pulse 38.5 degrees
Acq. time 2.674 sec
Width 24509.8 Hz
13936 repetitions

OBSERVE C13, 100.4576840
DECOUPLE H1, 399.5150667
Power 37 dB
continuously on
WALTZ-16 modulated

DATA PROCESSING
Line broadening 1.0 Hz
FT size 262144
Total time 16.2 hours

MMTC11a
in DMSO

Sample Name:
MMTC11a
Data Collected on:
400MR-vnmrs400
Archive directory: [illegible]

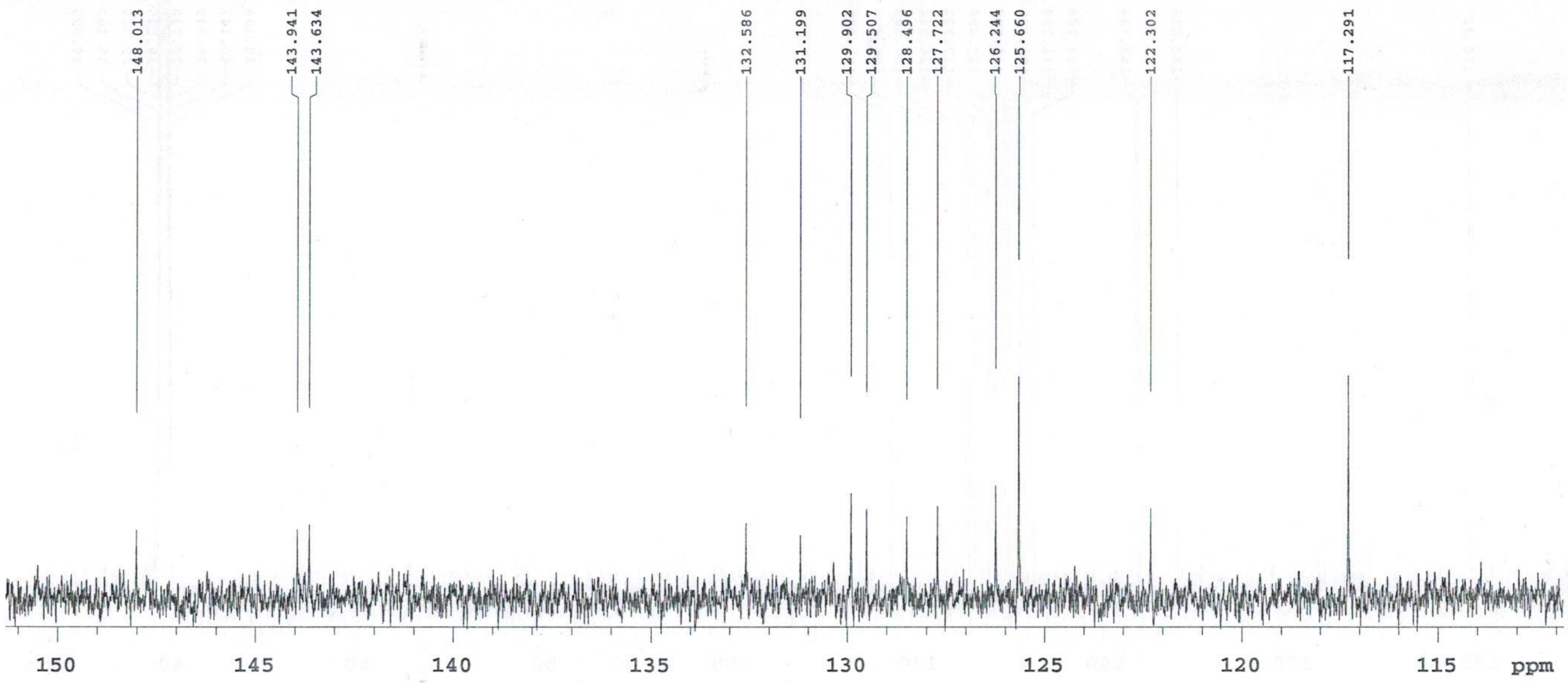

PULSE SEQUENCE
Relax. delay 1.500 sec
Pulse 38.5 degrees
Acq. time 2.674 sec
Width 24509.8 Hz
13936 repetitions

OBSERVE C13, 100.4576840
DECOUPLE H1, 399.5150667
Power 37 dB
continuously on
WALTZ-16 modulated

DATA PROCESSING
Line broadening 1.0 Hz
FT size 262144
Total time 16.2 hours

MMTC11a
in DMSO

Sample Name:
MMTC11a
Data Collected on:
400MR-vnmrs400
Archive directory:
[illegible]

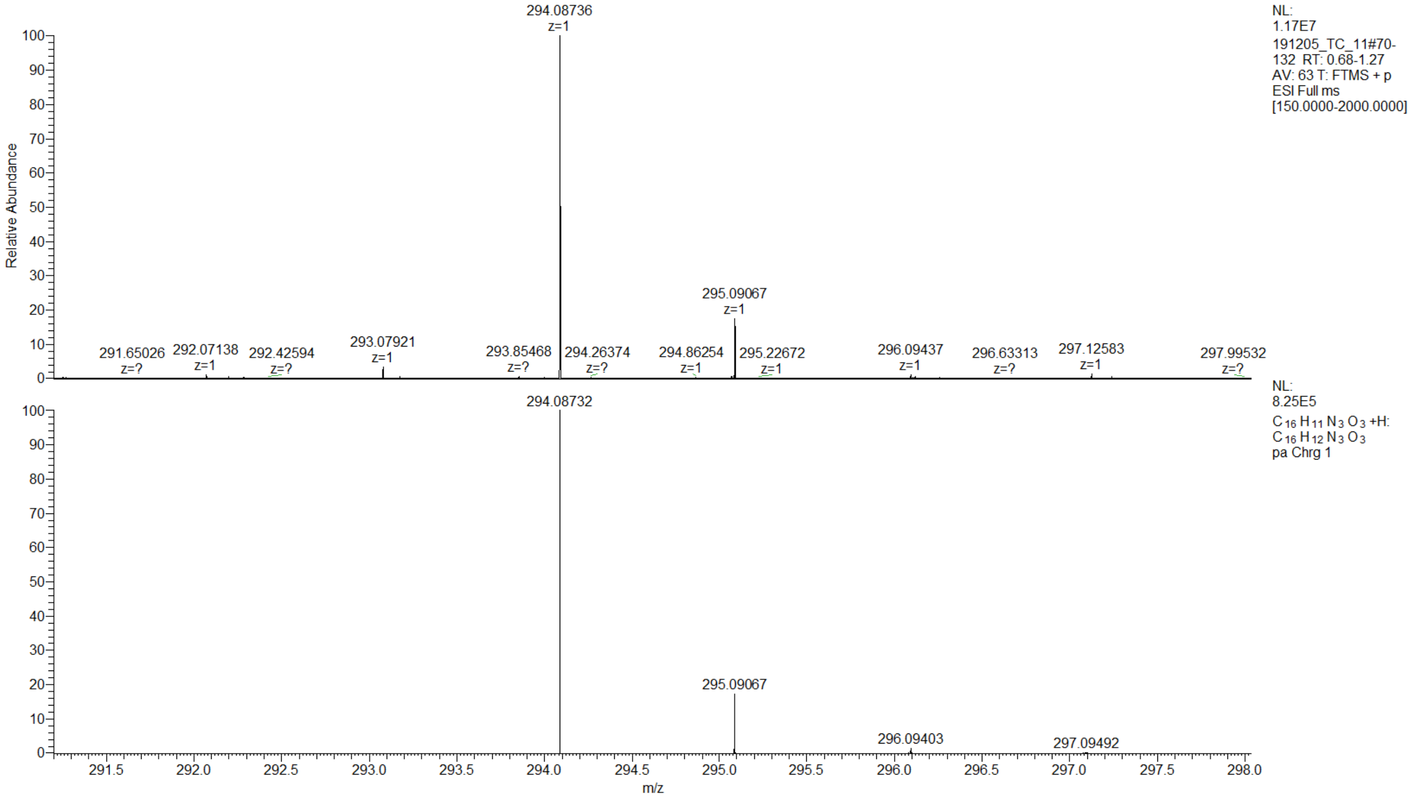

NL:
1.17E7
191205_TC_11#70-132 RT: 0.68-1.27
AV: 63 T: FTMS + p
ESI Full ms
[150.0000-2000.0000]
Relative Abundance
294.08736
z=1
295.09067
z=1
291.65026
z=?
292.07138
z=1
292.42594
z=?
293.07921
z=1
293.85468
z=?
294.26374
z=?
294.86254
z=1
295.22672
z=1
296.09437
z=1
296.63313
z=?
297.12583
z=1
297.99532
z=?
NL:
8.25E5
C 16 H 11 N 3 O 3 +H:
C 16 H 12 N 3 O 3
pa Chrg 1
294.08732
295.09067
296.09403
297.09492
m/z

Compound **TR-4**

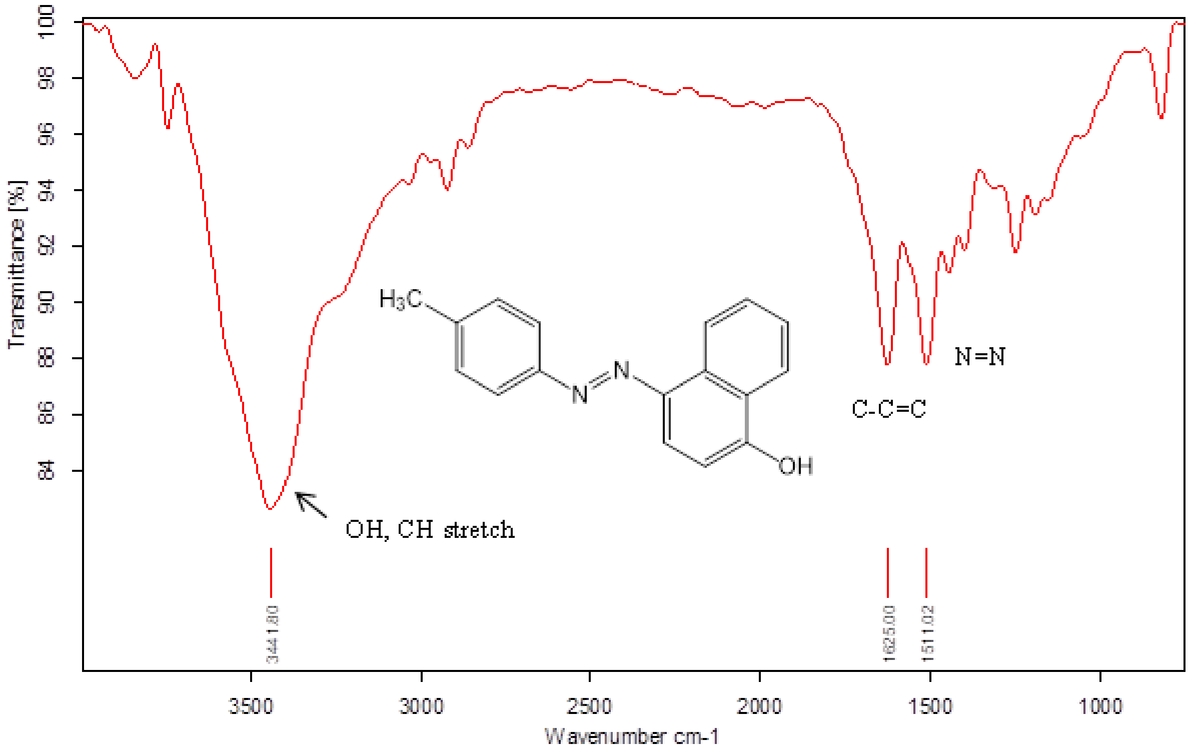

TC: 12	$C_{17}H_{14}N_2O$	262.3	IS	PS	S	S	S	S	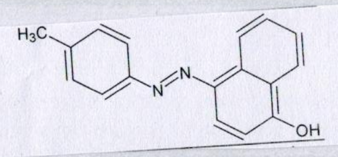

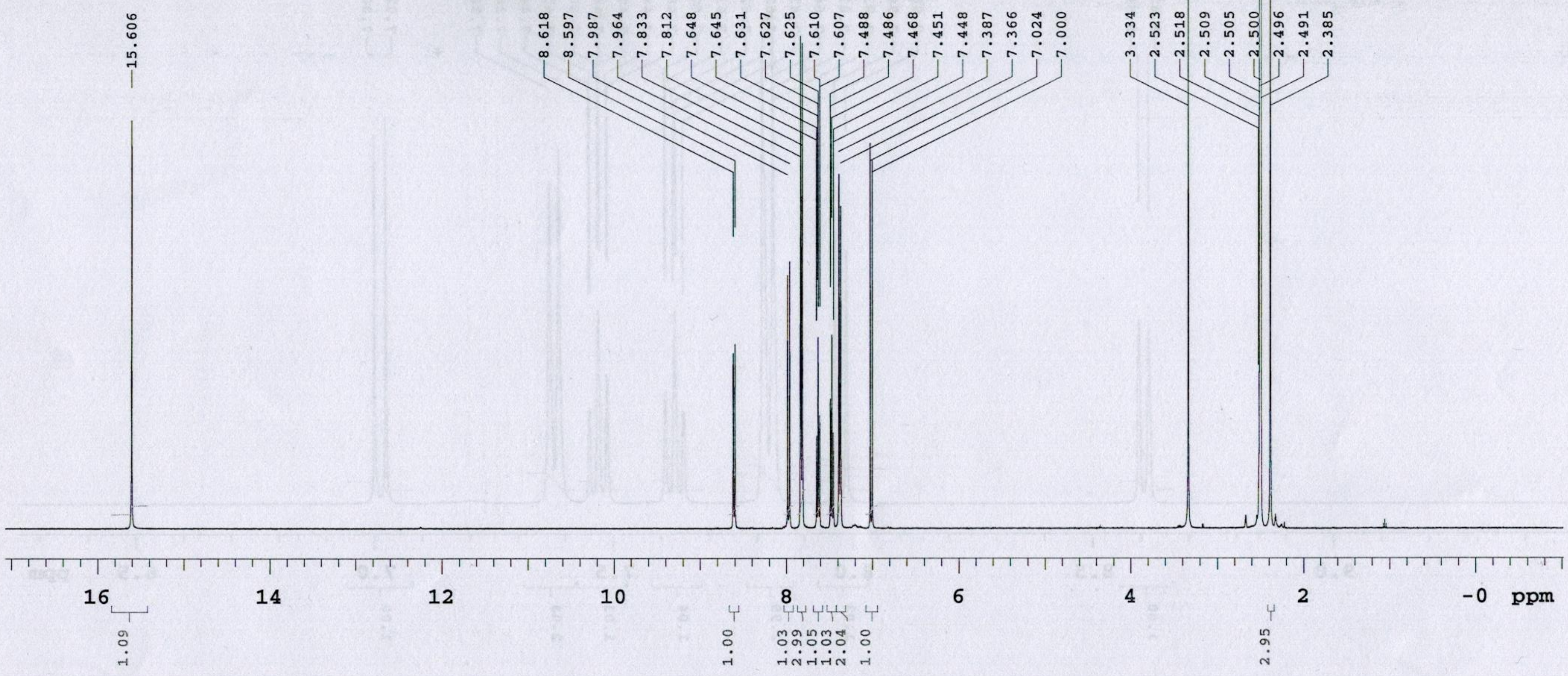

PULSE SEQUENCE
Relax. delay 0.500 sec
Pulse 48.6 degrees
Acq. time 4.797 sec
Width 7225.4 Hz
20 repetitions

OBSERVE H1, 399.5130588

DATA PROCESSING
FT size 65536
Total time 1 minutes

MMTC12a
in DMSO

Sample Name:
MMTC12a
Data Collected on:
400MR-vnmrs400
[illegible]

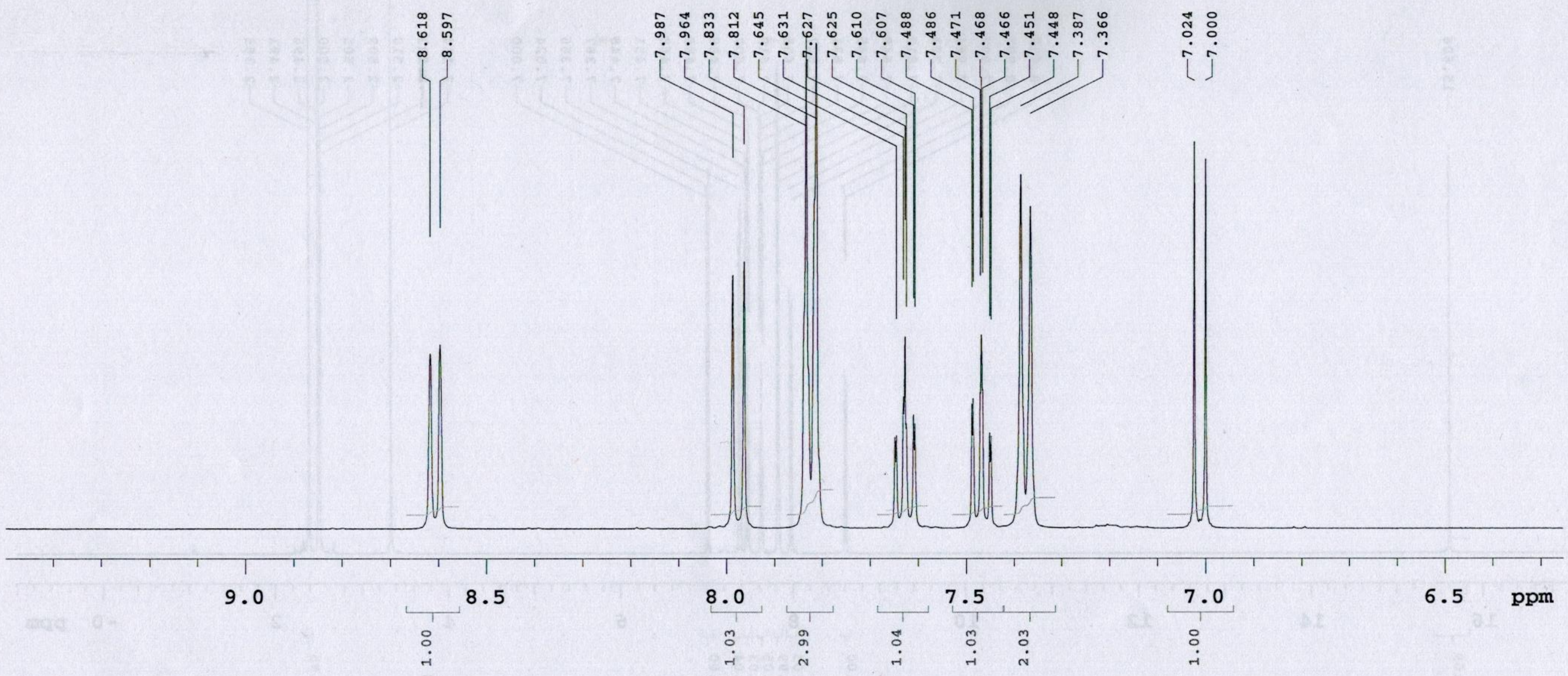

PULSE SEQUENCE
Relax. delay 0.500 sec
Pulse 48.6 degrees
Acq. time 4.797 sec
Width 7225.4 Hz
32 repetitions

OBSERVE H1, 399.5130588

DATA PROCESSING
FT size 65536
Total time 2 minutes

MMTC12a
in DMSO

Sample Name:
MMTC12a
Data Collected on:
400MR-vnmrs400
Archive directory: [illegible]

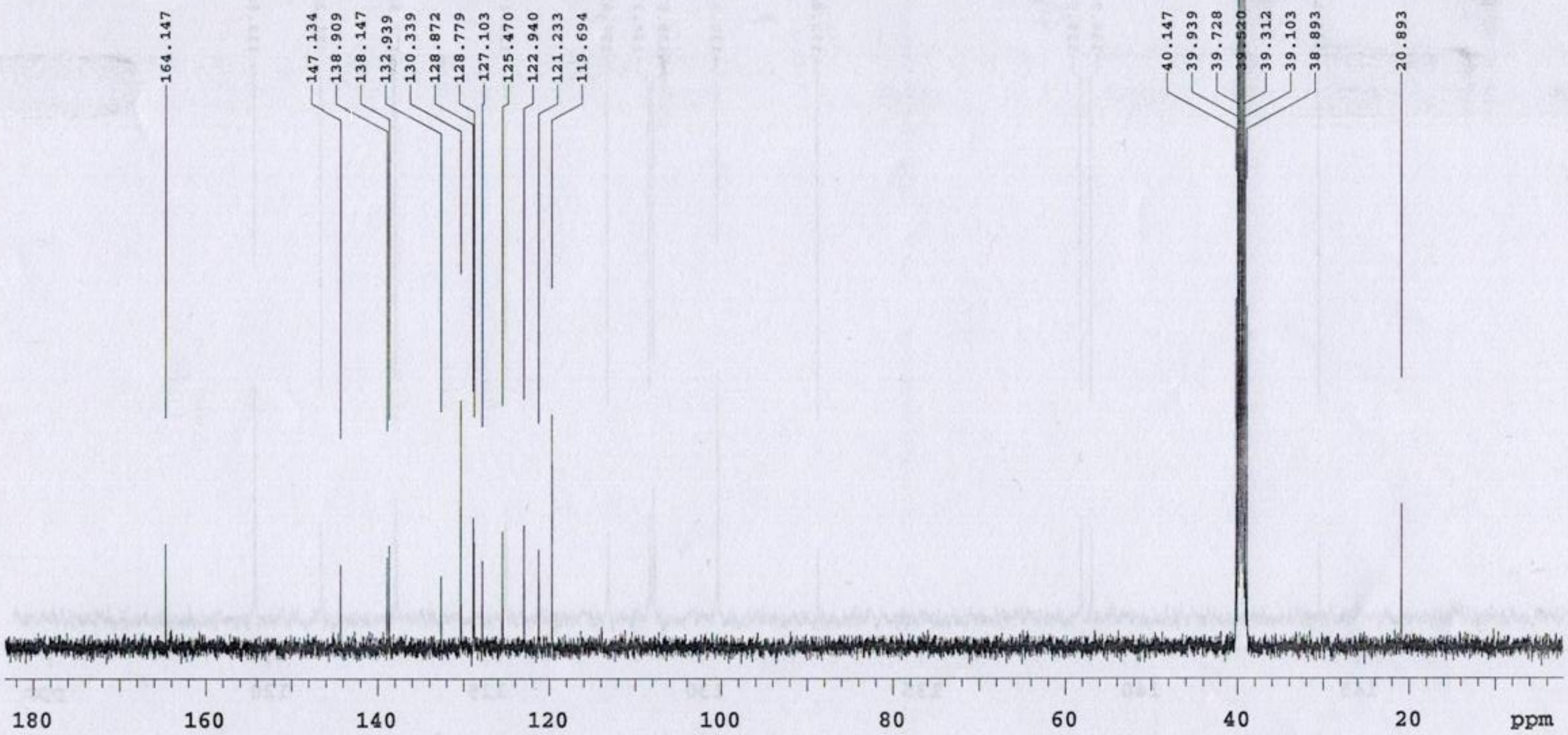

PULSE SEQUENCE
Relax. delay 1.500 sec
Pulse 38.5 degrees
Acq. time 2.674 sec
Width 24509.8 Hz
1200 repetitions

OBSERVE C13, 100.4576840
DECOUPLE H1, 399.5150667
Power 37 dB
continuously on
WALTZ-16 modulated

DATA PROCESSING
Line broadening 1.0 Hz
FT size 262144
Total time 83 minutes

MMTC 12
in DMSO

Sample Name:
MMTC 12
Data Collected on:
400MR-vnmrs400
[illegible] (429020

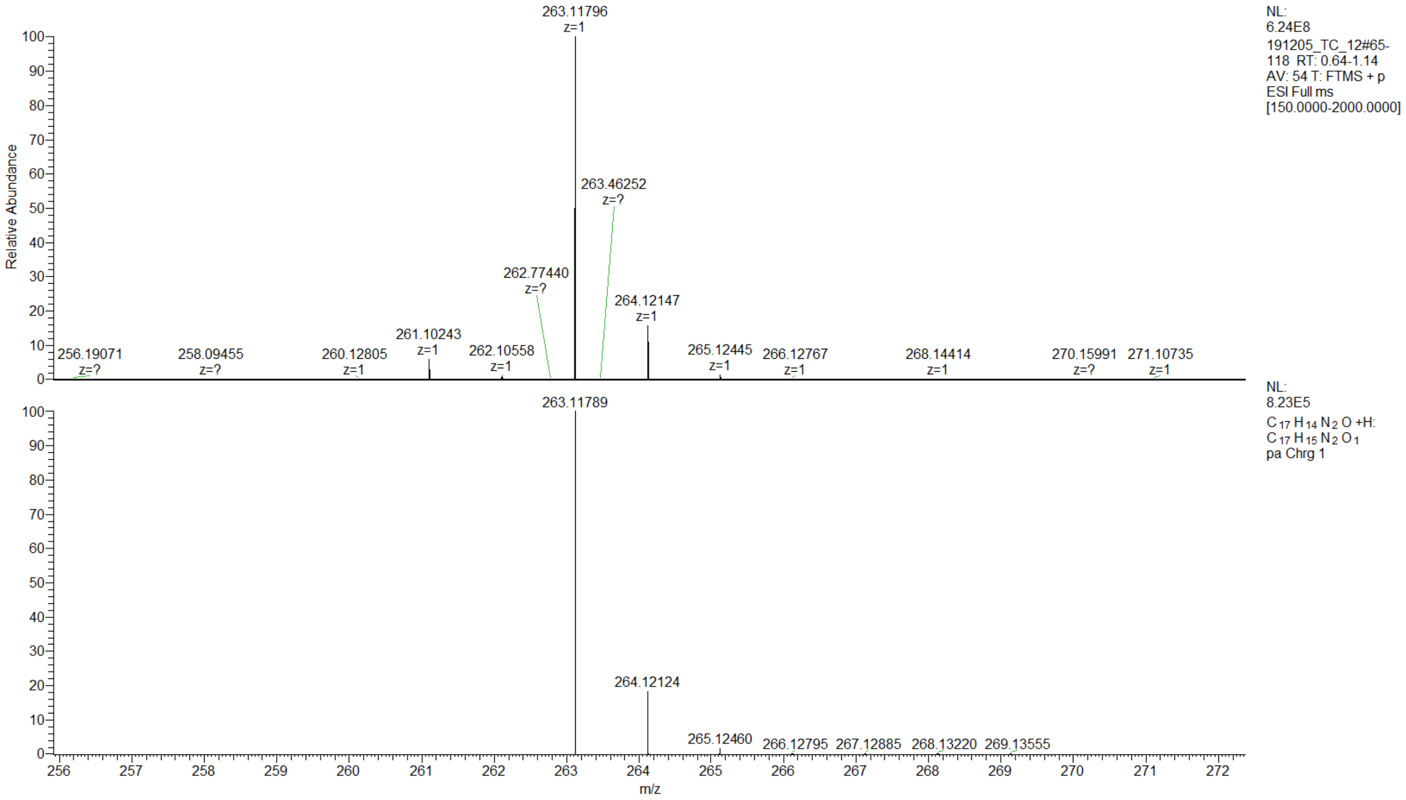
263.11796
z=1
NL:
6.24E8
191205_TC_12#65-118 RT: 0.64-1.14
AV: 54 T: FTMS + p
ESI Full ms
[150.0000-2000.0000]
Relative Abundance
263.46252
z=?
262.77440
z=?
264.12147
z=1
261.10243
z=1
256.19071
z=?
258.09455
z=?
260.12805
z=1
262.10558
z=1
265.12445
z=1
266.12767
z=1
268.14414
z=1
270.15991
z=?
271.10735
z=1
NL:
8.23E5
C 17 H 14 N 2 O +H:
C 17 H 15 N 2 O 1
pa Chrg 1
263.11789
264.12124
265.12460
266.12795
267.12885
268.13220
269.13555
m/z

Compound **TR-5**

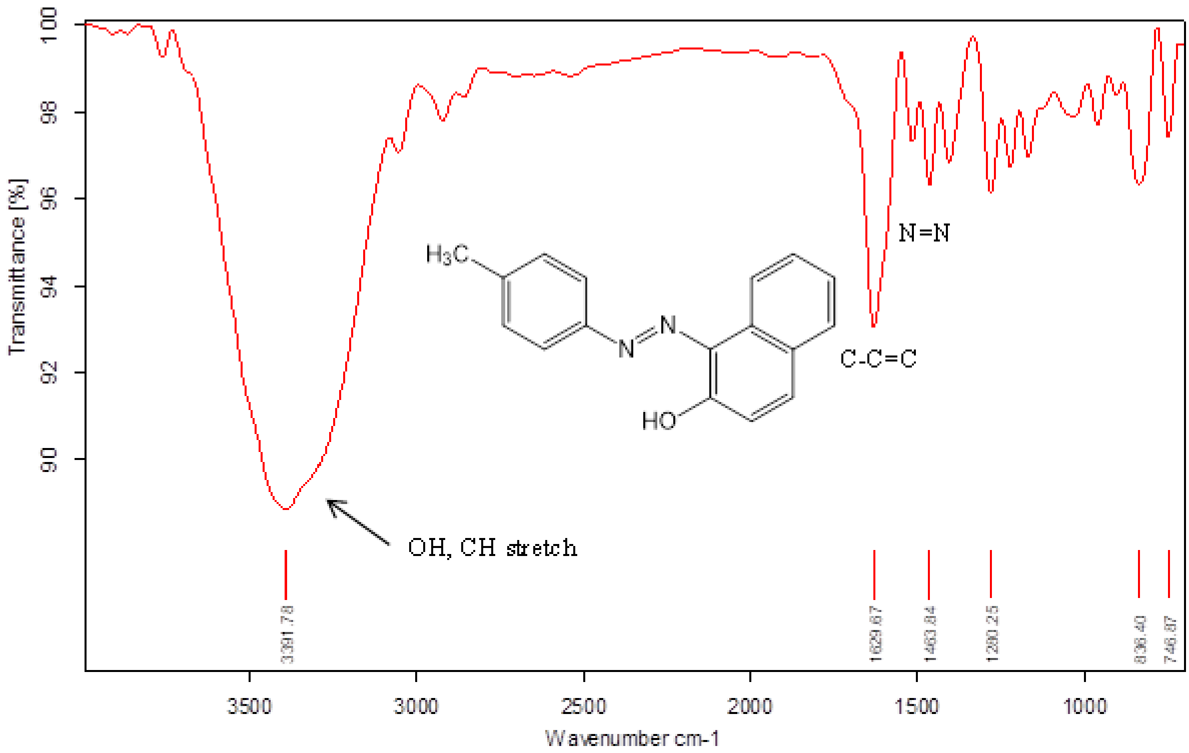

TC: 13	$C_{17}H_{14}N_2O$	262.3	IS	PS	S	S	S	S	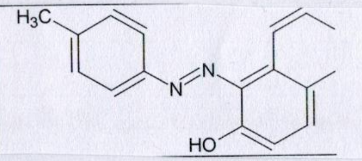

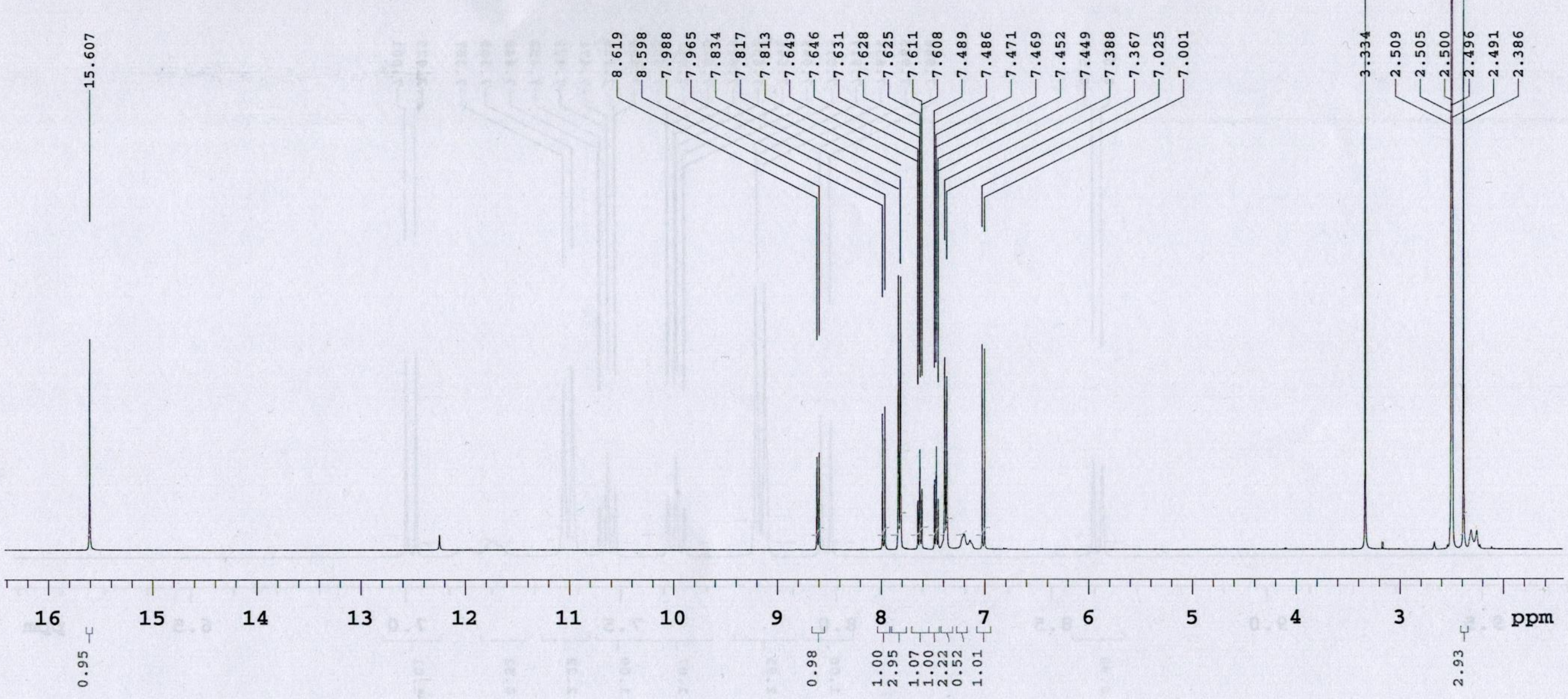

PULSE SEQUENCE
Relax. delay 0.500 sec
Pulse 48.6 degrees
Acq. time 4.797 sec
Width 7225.4 Hz
36 repetitions

OBSERVE H1, 399.5130588

DATA PROCESSING
FT size 65536
Total time 3 minutes

MMTC13a
in DMSO

Sample Name:
MMTC13a
Data Collected on:
400MR-vnmrs400
Archive directory:
[illegible]

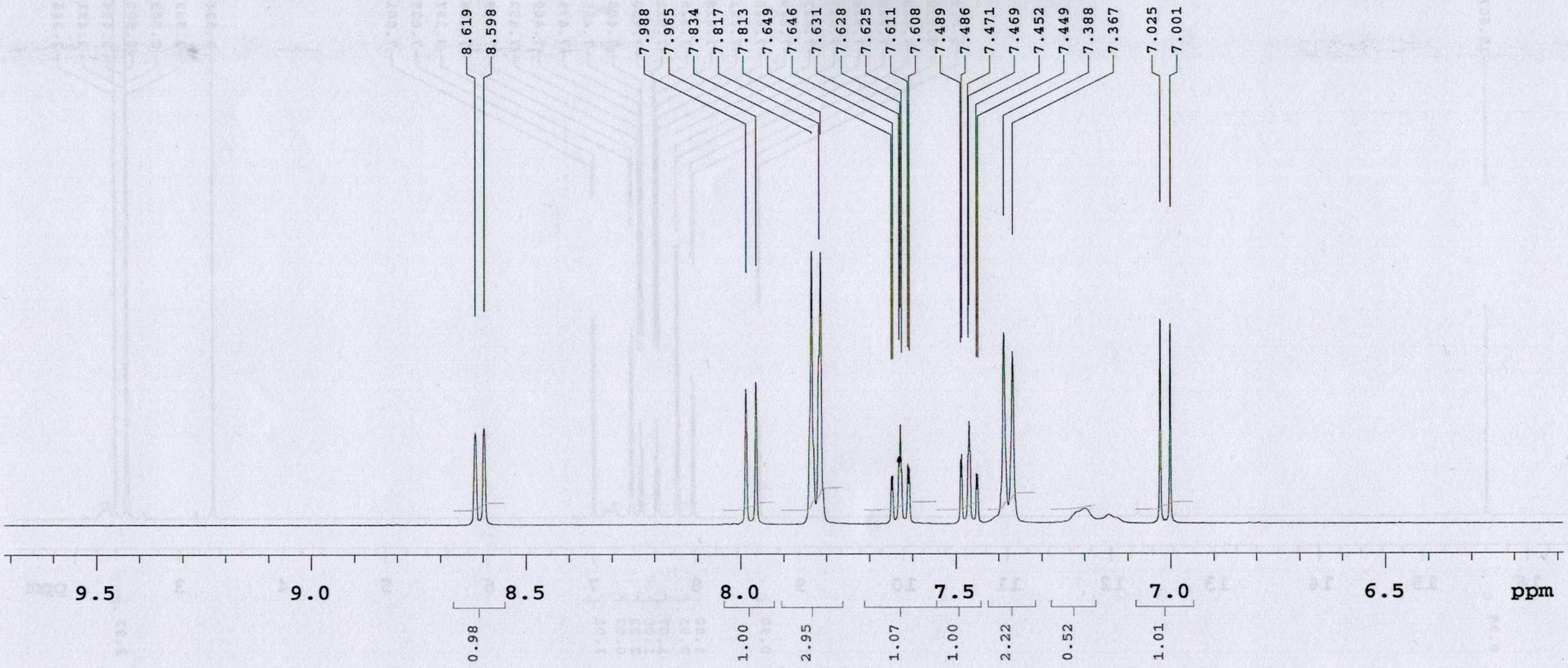

PULSE SEQUENCE
Relax. delay 0.500 sec
Pulse 48.6 degrees
Acq. time 4.797 sec
Width 7225.4 Hz
28 repetitions

OBSERVE H1, 399.5130588

DATA PROCESSING
FT size 65536
Total time 2 minutes

MMTC13a
in DMSO

Sample Name:
MMTC13a
Data Collected on:
400MR-vnmrs400
[illegible]

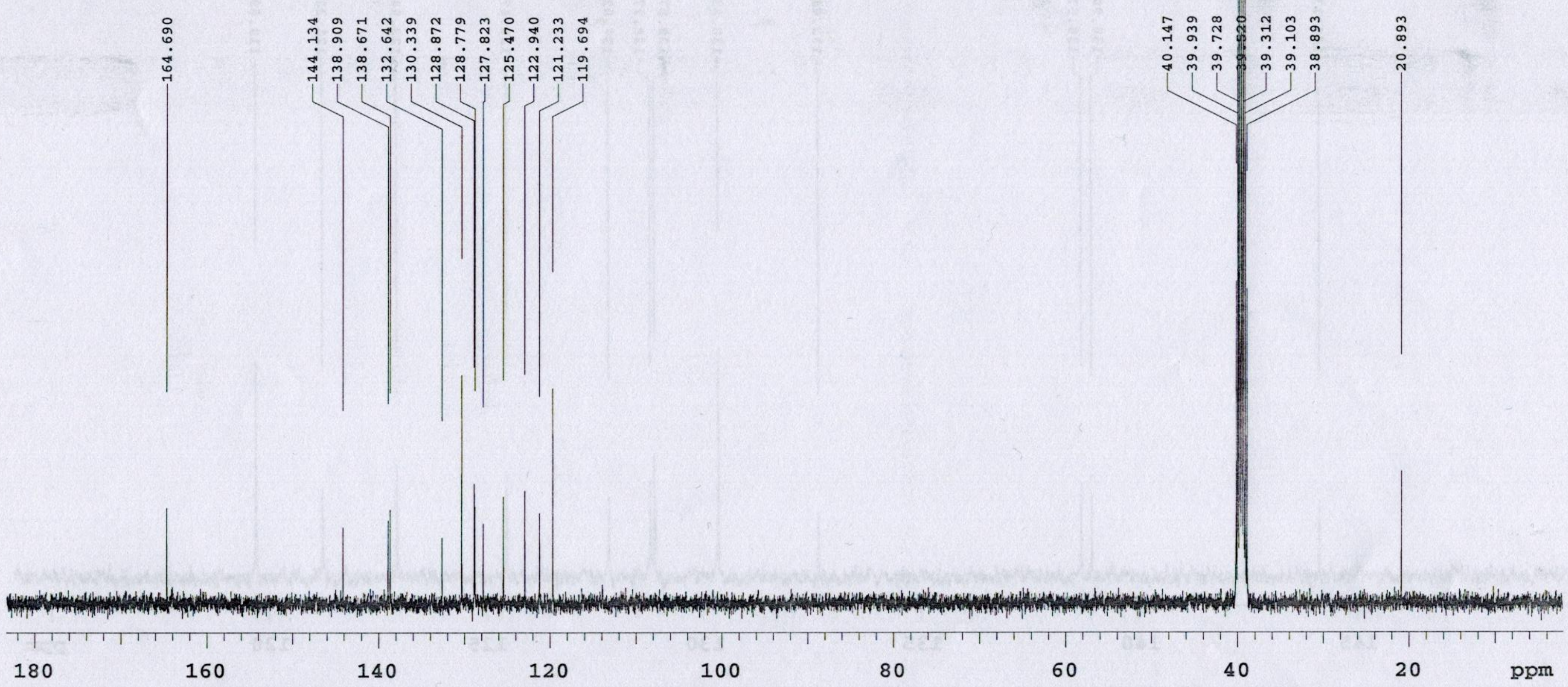

PULSE SEQUENCE
Relax. delay 1.500 sec
Pulse 38.5 degrees
Acq. time 2.674 sec
Width 24509.8 Hz
1200 repetitions

OBSERVE C13, 100.4576840
DECOUPLE H1, 399.5150667
Power 37 dB
continuously on
WALTZ-16 modulated

DATA PROCESSING
Line broadening 1.0 Hz
FT size 262144
Total time 83 minutes

MMTC13a
in DMSO

Sample Name:
MMTC13a
Data Collected on:
400MR-vnmrs400
[illegible] (429020)

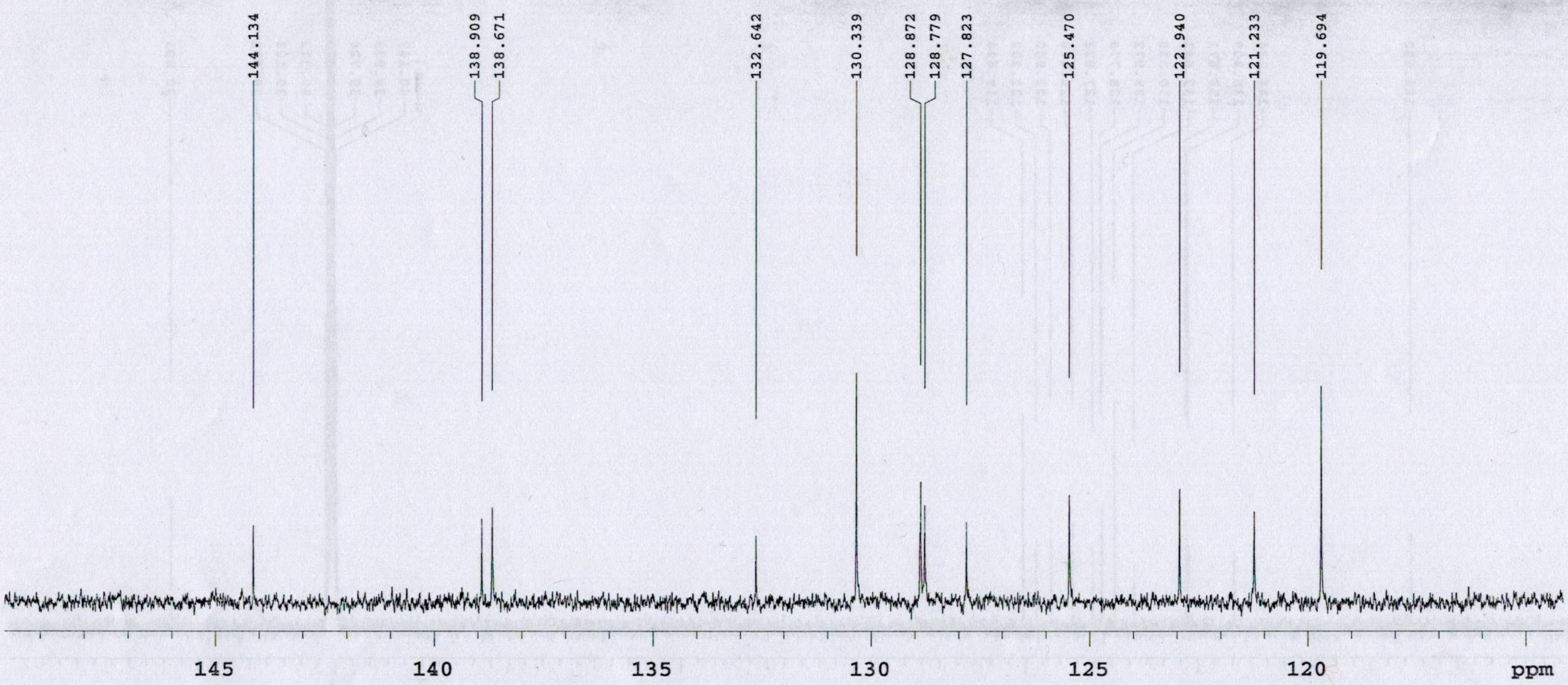

PULSE SEQUENCE
Relax. delay 1.500 sec
Pulse 38.5 degrees
Acq. time 2.674 sec
Width 24509.8 Hz
1216 repetitions

OBSERVE C13, 100.4576840
DECOUPLE H1, 399.5150667
Power 37 dB
continuously on
WALTZ-16 modulated

DATA PROCESSING
Line broadening 1.0 Hz
FT size 262144
Total time 84 minutes

MMTC13a
in DMSO

Sample Name:
MMTC13a
Data Collected on:
400MR-vnmrs400
Archive directory:
[illegible]

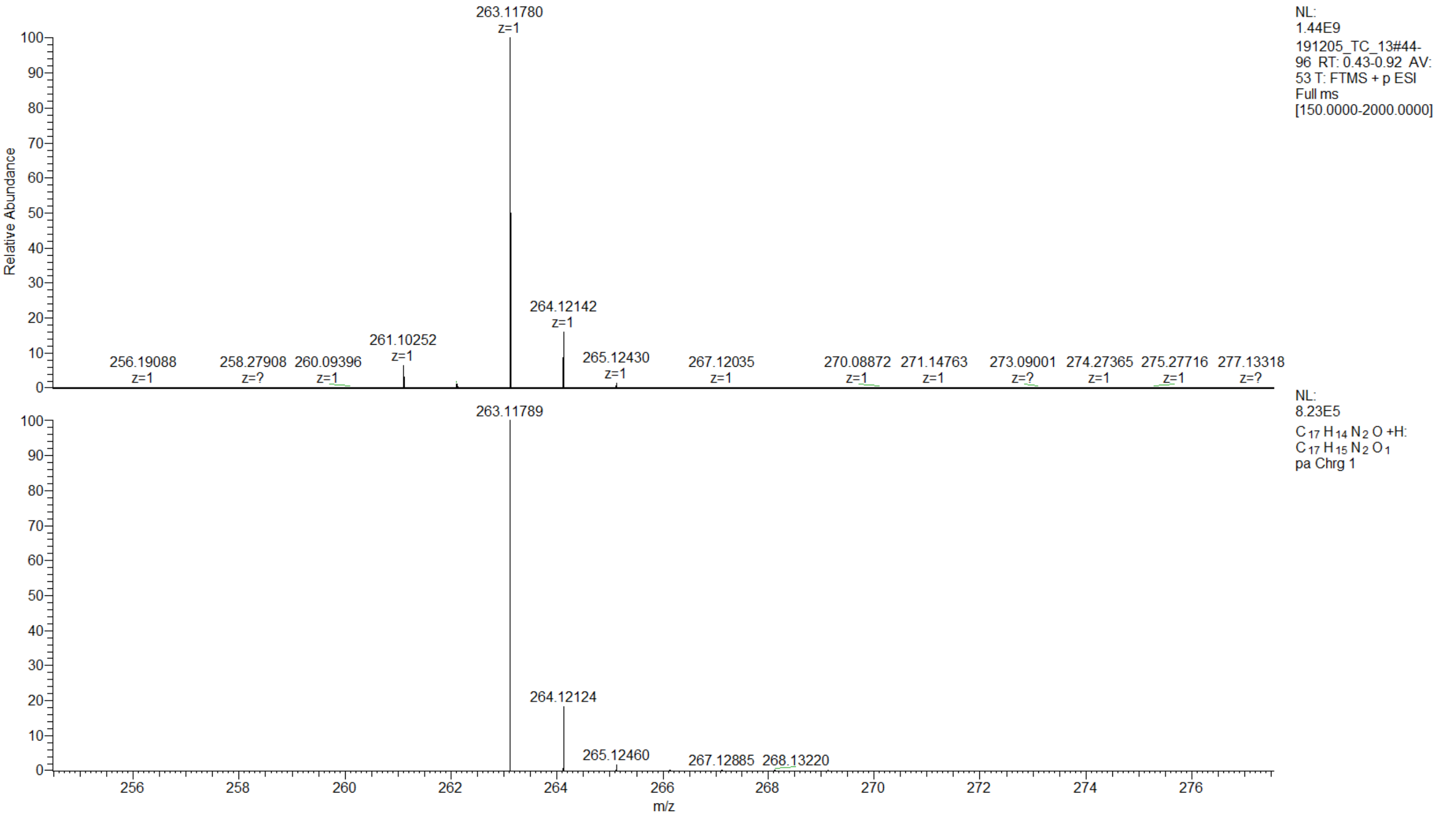
263.11780
z=1
NL:
1.44E9
191205_TC_13#44-96 RT: 0.43-0.92 AV: 53 T: FTMS + p ESI Full ms [150.0000-2000.0000]
Relative Abundance
264.12142
z=1
261.10252
z=1
265.12430
z=1
256.19088
z=1
258.27908
z=?
260.09396
z=1
267.12035
z=1
270.08872
z=1
271.14763
z=1
273.09001
z=?
274.27365
z=1
275.27716
z=1
277.13318
z=?
NL:
8.23E5
C 17 H 14 N 2 O +H:
C 17 H 15 N 2 O 1
pa Chrg 1
263.11789
264.12124
265.12460
267.12885
268.13220
m/z

Compound **TR-6**

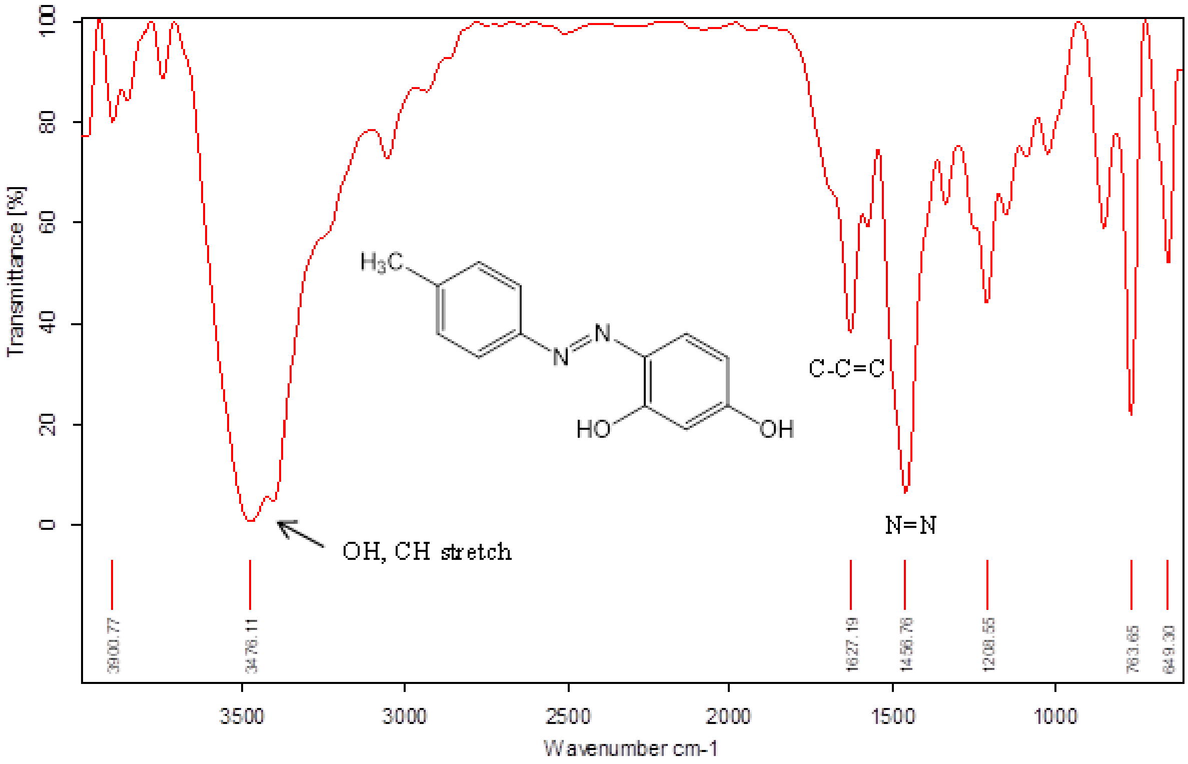

TC: 14	$C_{13}H_{12}N_2O_2$	228.2	IS	PS	S	S	S	S	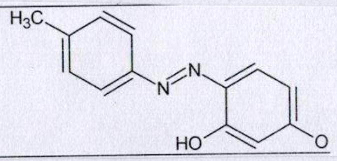

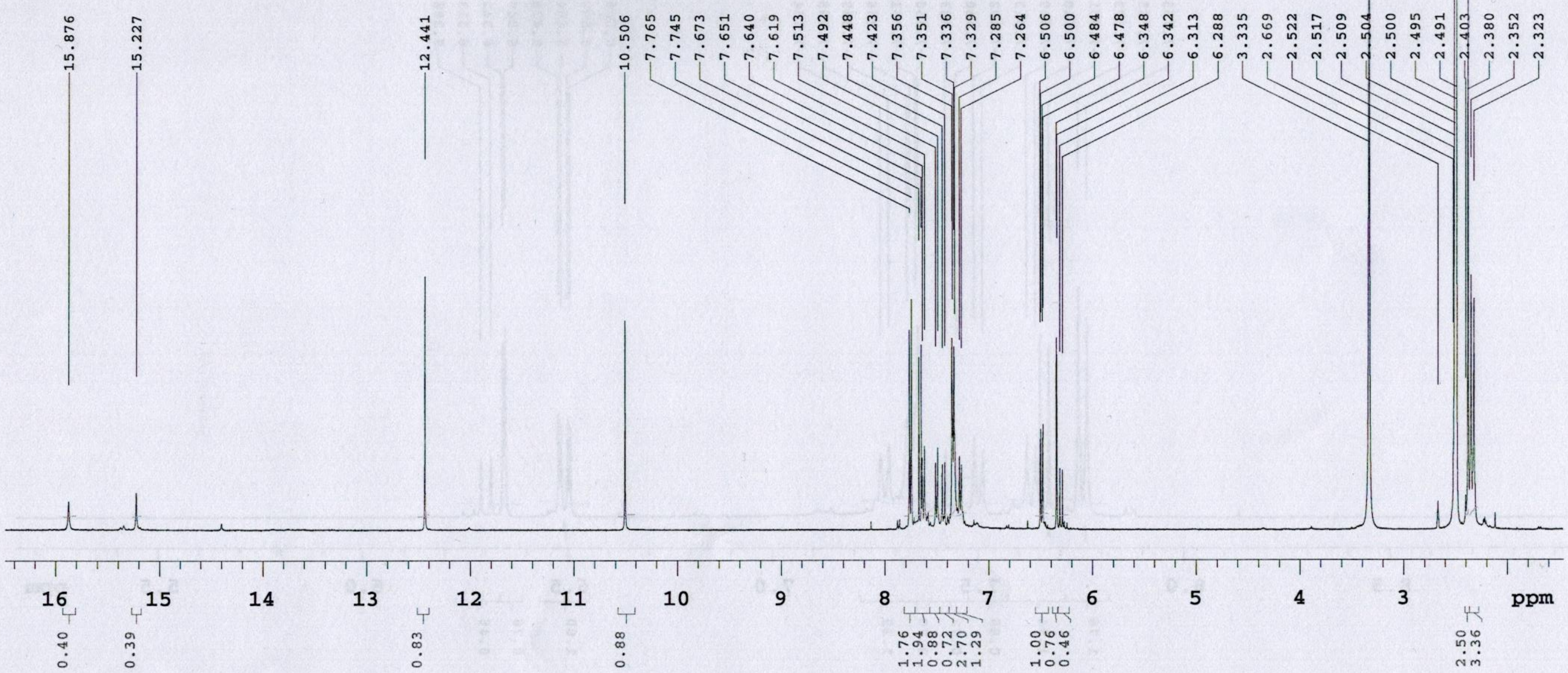

PULSE SEQUENCE
Relax. delay 0.500 sec
Pulse 48.6 degrees
Acq. time 4.797 sec
Width 7225.4 Hz
40 repetitions

OBSERVE H1, 399.5130591

DATA PROCESSING
FT size 65536
Total time 3 minutes

MMTC14a
in DMSO

Sample Name:
MMTC14a
Data Collected on:
400MR-vnmrs400
Archive directory: [illegible]

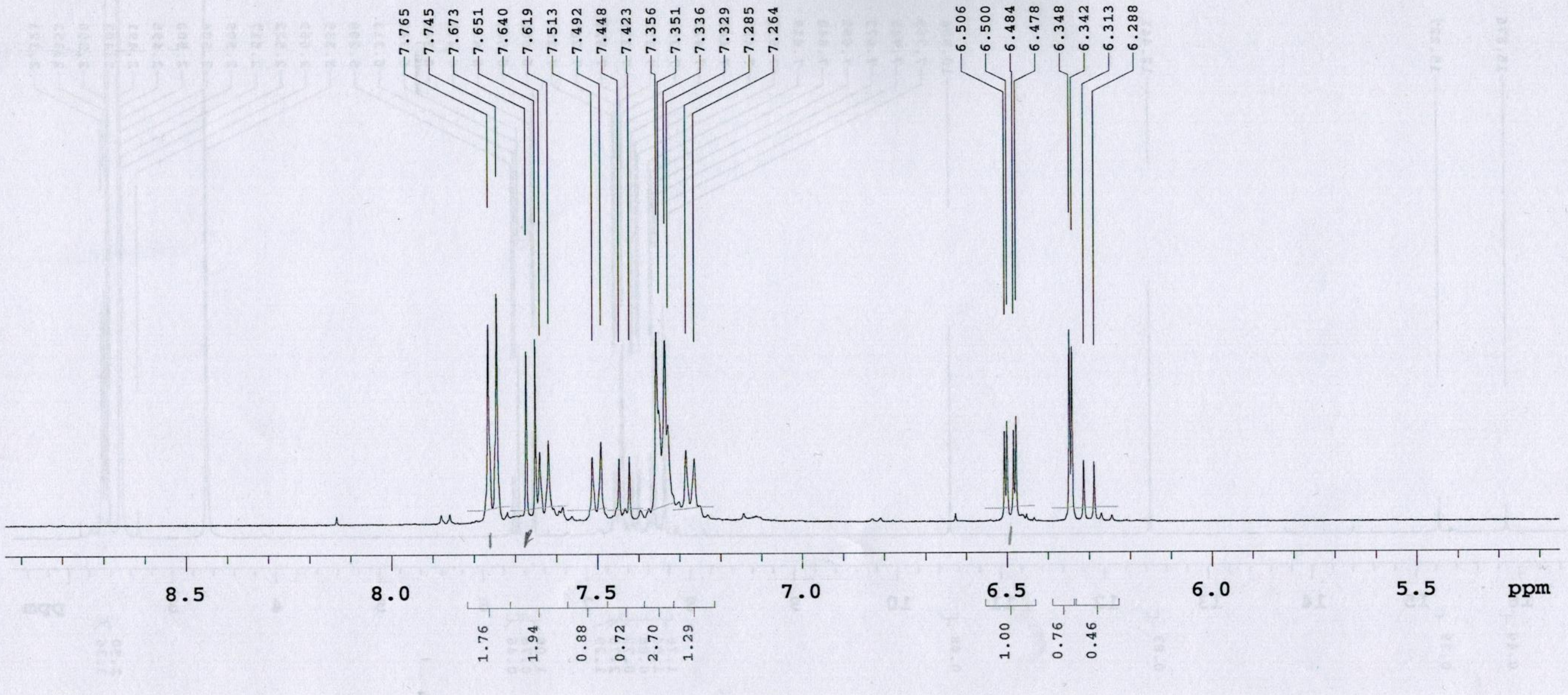

PULSE SEQUENCE
Relax. delay 0.500 sec
Pulse 48.6 degrees
Acq. time 4.797 sec
Width 7225.4 Hz
40 repetitions

OBSERVE H1, 399.5130591

DATA PROCESSING
FT size 65536
Total time 3 minutes

MMTC14a
in DMSO

Sample Name:
MMTC14a
Data Collected on:
400MR-vnmrs400
Archive directory: [illegible]

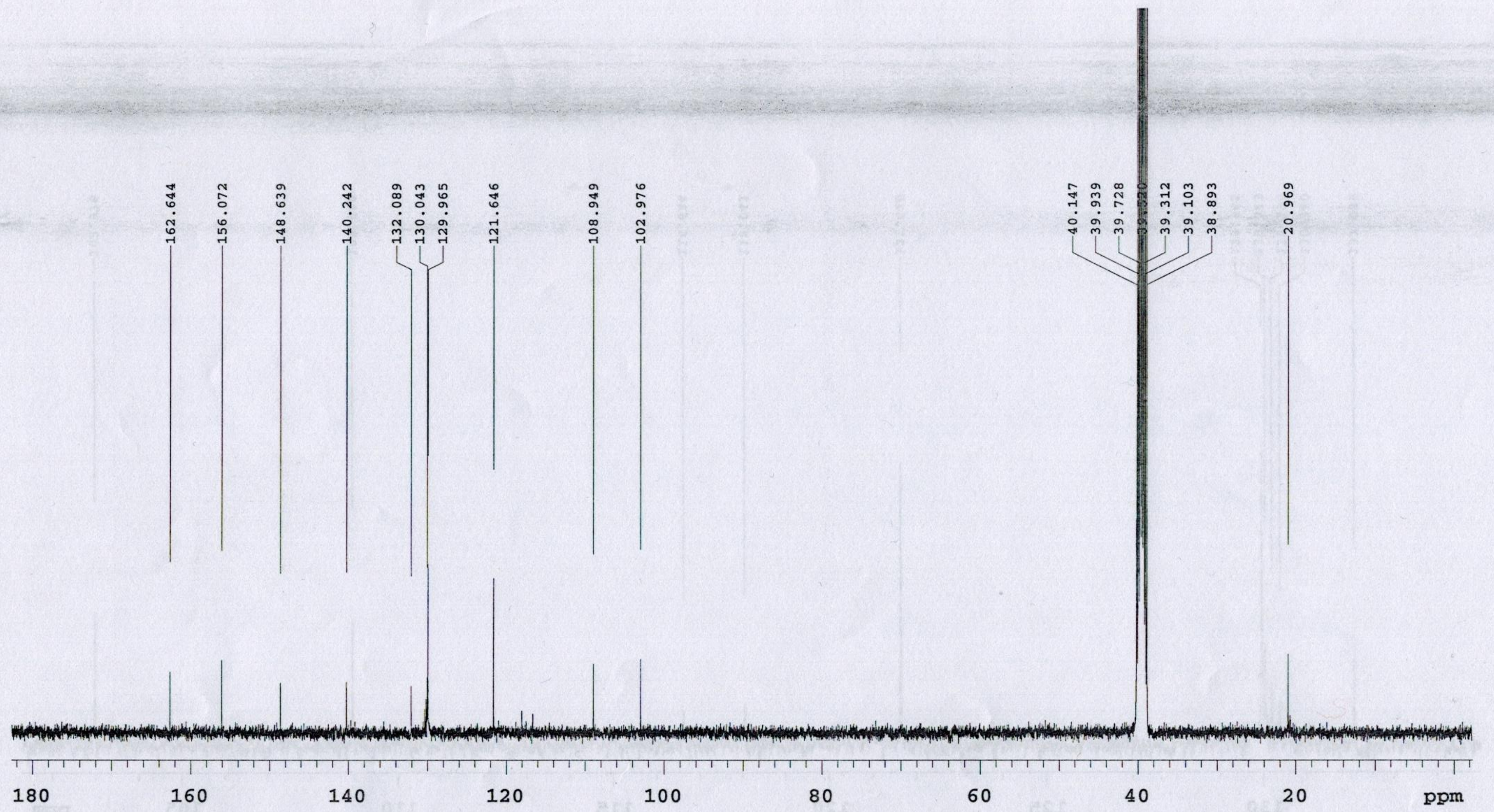

PULSE SEQUENCE
Relax. delay 1.500 sec
Pulse 38.5 degrees
Acq. time 2.674 sec
Width 24509.8 Hz
14000 repetitions

OBSERVE C13, 100.4576836
DECOUPLE H1, 399.5150667
Power 37 dB
continuously on
WALTZ-16 modulated

DATA PROCESSING
Line broadening 1.0 Hz
FT size 262144
Total time 16.2 hours

MMTC14a
in DMSO

Sample Name:
MMTC14a
Data Collected on:
400MR-vnmrs400
Archive directory: [illegible]

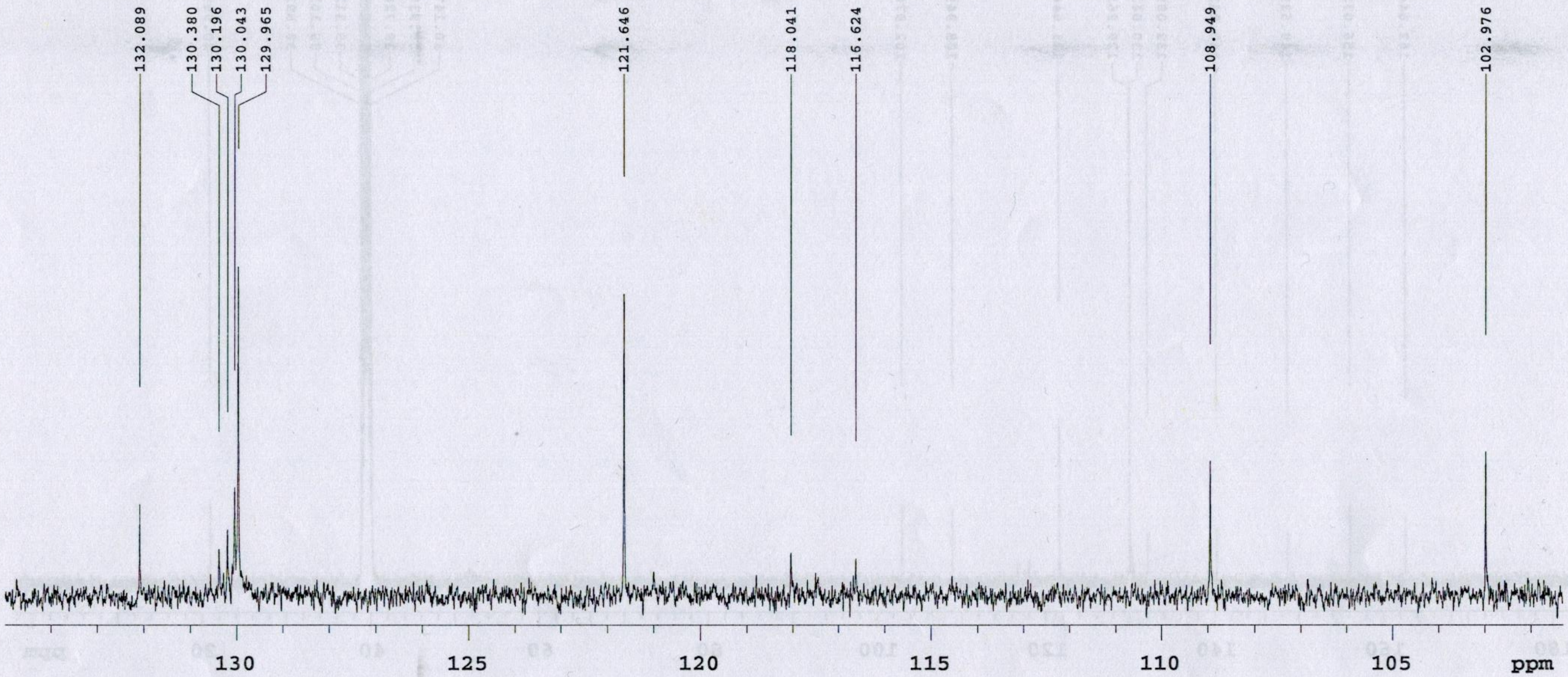

PULSE SEQUENCE
Relax. delay 1.500 sec
Pulse 38.5 degrees
Acq. time 2.674 sec
Width 24509.8 Hz
14000 repetitions

OBSERVE C13, 100.4576836
DECOUPLE H1, 399.5150667
Power 37 dB
continuously on
WALTZ-16 modulated

DATA PROCESSING
Line broadening 1.0 Hz
FT size 262144
Total time 16.2 hours

MMTC14a
in DMSO

Sample Name:
MMTC14a
Data Collected on:
400MR-vnmrs400
[illegible]

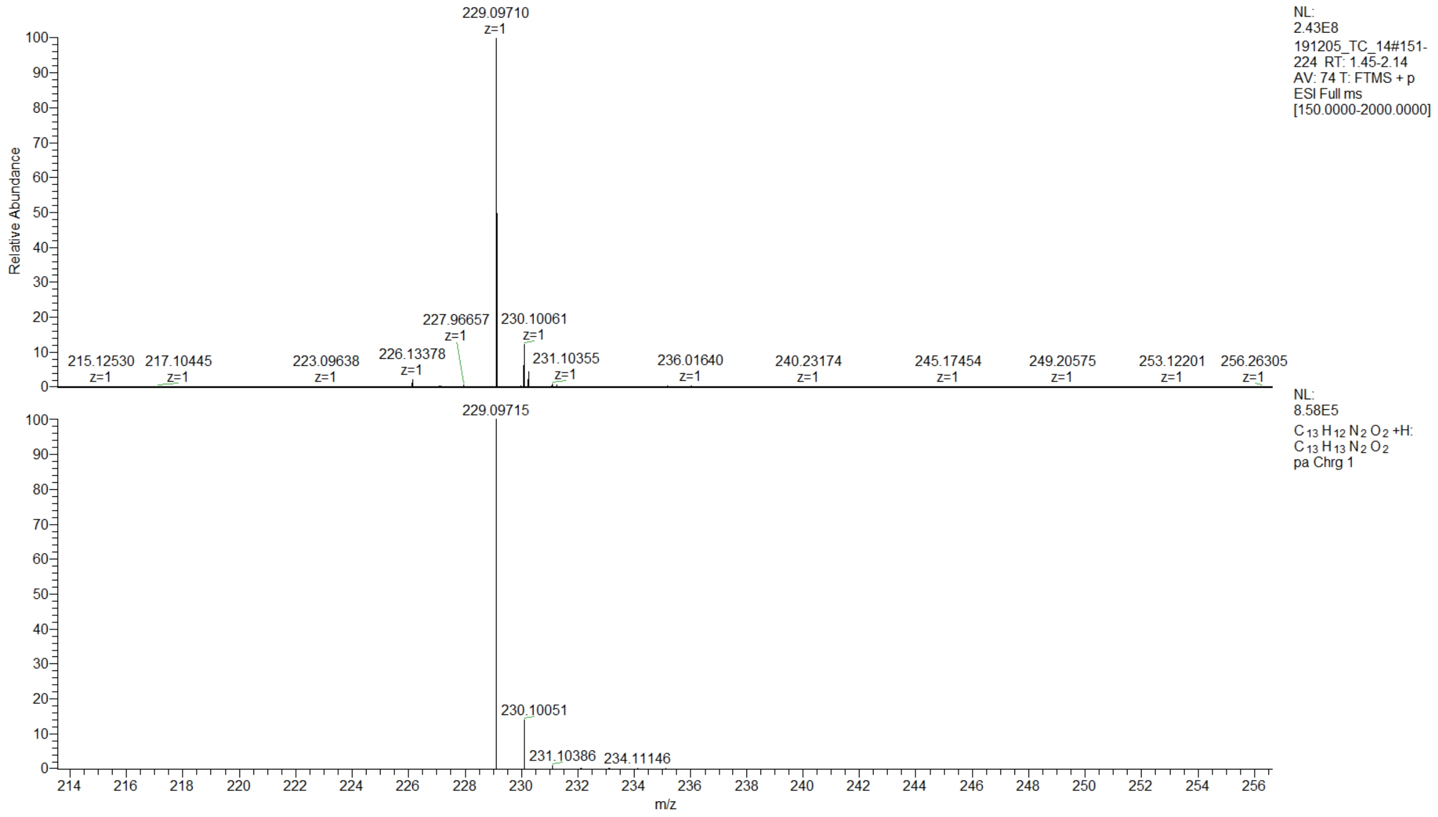
229.09710
z=1
NL:
2.43E8
191205_TC_14#151-224 RT: 1.45-2.14
AV: 74 T: FTMS + p ESI Full ms [150.0000-2000.0000]
Relative Abundance
215.12530
217.10445
223.09638
226.13378
227.96657
230.10061
231.10355
236.01640
240.23174
245.17454
249.20575
253.12201
256.26305
NL:
8.58E5
C 13 H 12 N 2 O 2 +H:
C 13 H 13 N 2 O 2
pa Chrg 1
229.09715
230.10051
231.10386
234.11146
m/z

Compound **TR-7**

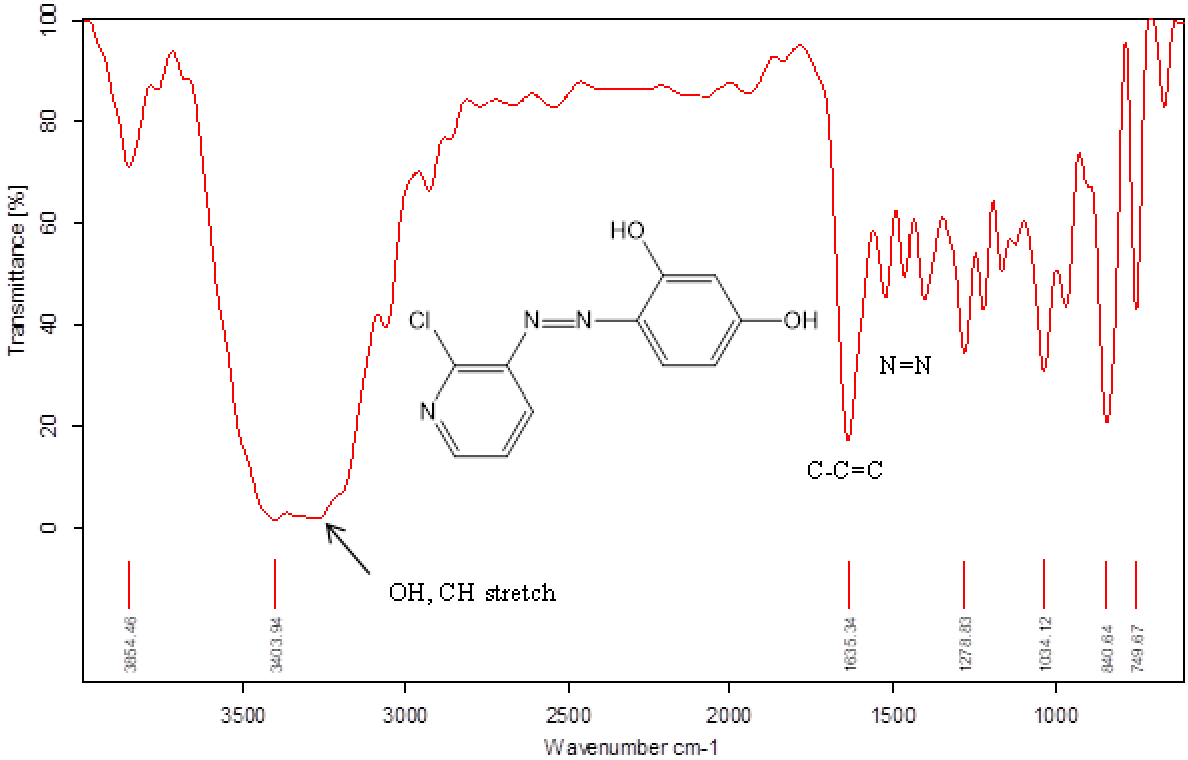

TC: 17	$C_{11}H_8ClN_3O_2$	249.65	IS	S	S	S	IS	S

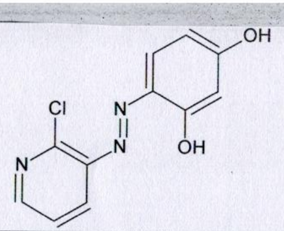

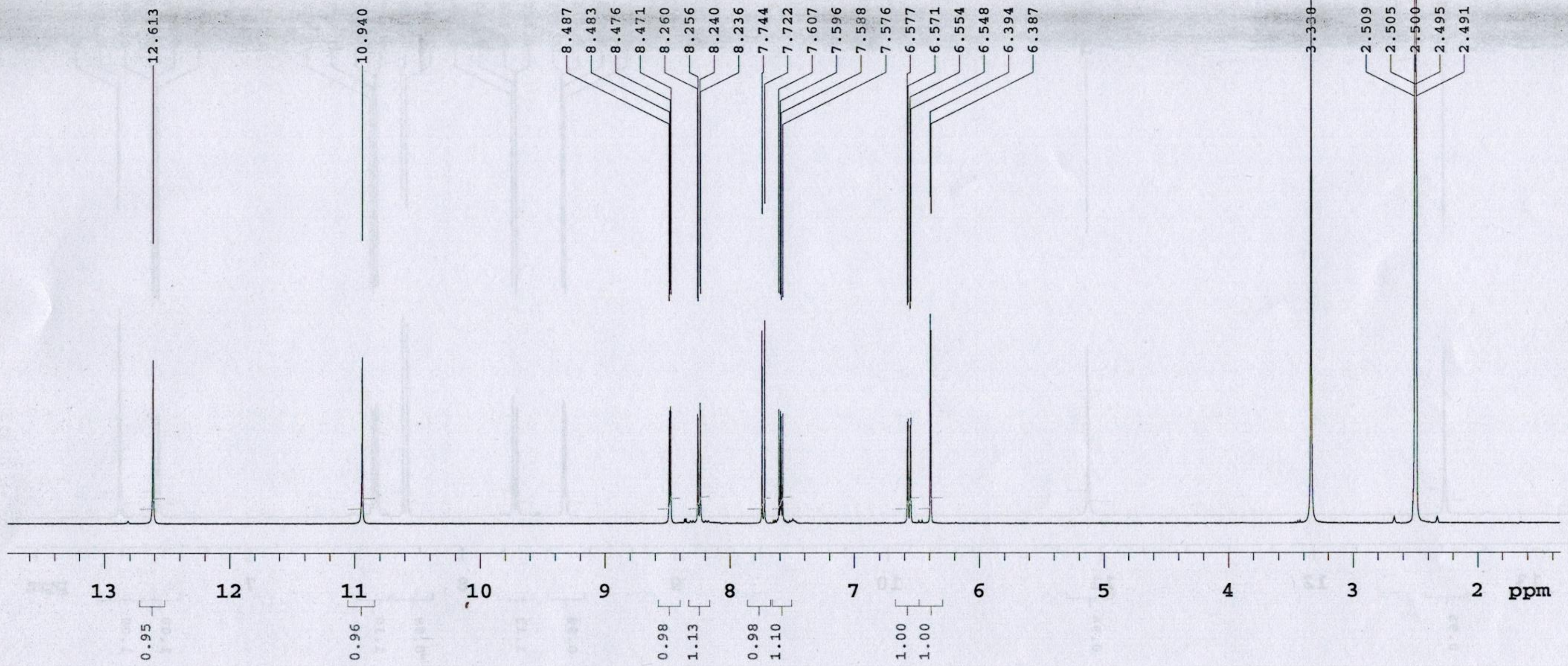

PULSE SEQUENCE
Relax. delay 0.500 sec
Pulse 48.6 degrees
Acq. time 4.797 sec
Width 6793.5 Hz
36 repetitions

OBSERVE H1, 399.5130588

DATA PROCESSING
FT size 65536
Total time 3 minutes

MMTC17a
in DMSO

Sample Name:
MMTC17a
Data Collected on:
400MR-vnmrs400
Archive directory: [illegible]

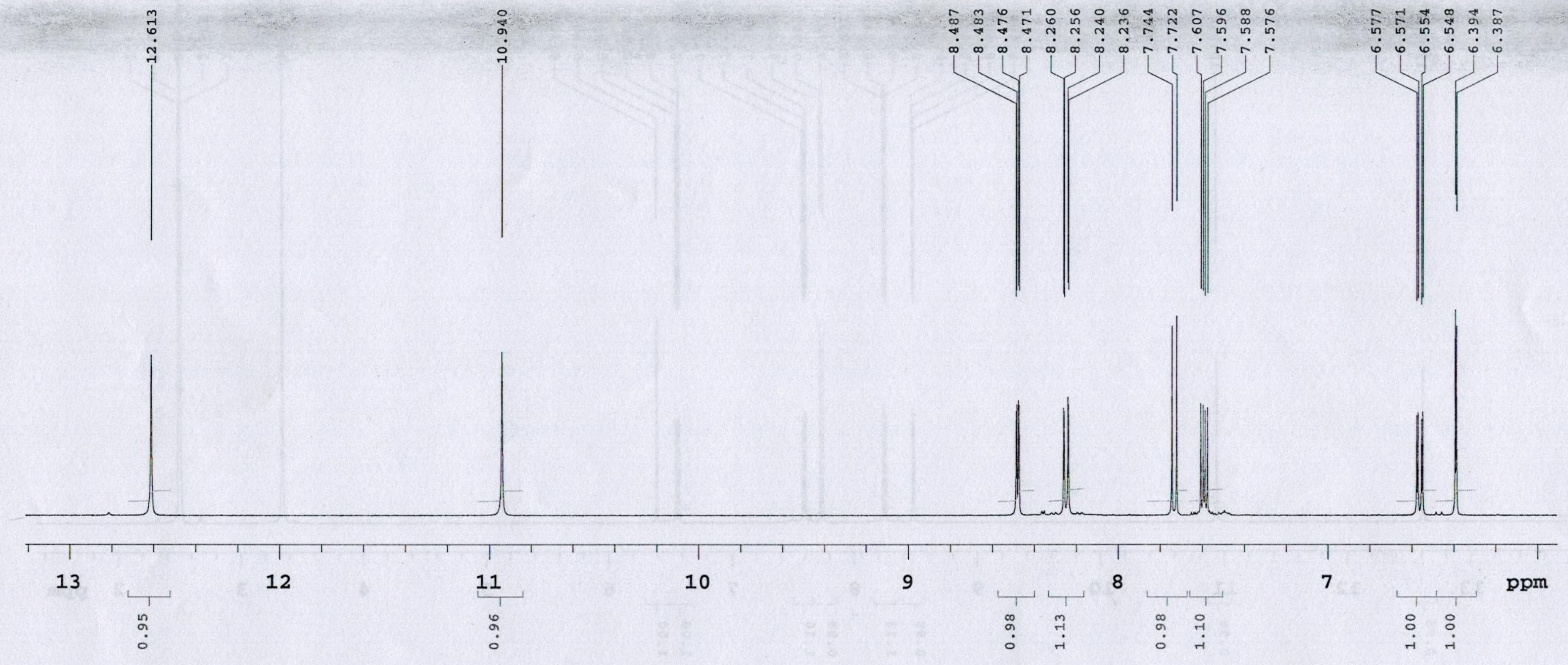

PULSE SEQUENCE
Relax. delay 0.500 sec
Pulse 48.6 degrees
Acq. time 4.797 sec
Width 6793.5 Hz
24 repetitions

OBSERVE H1, 399.5130588

DATA PROCESSING
FT size 65536
Total time 2 minutes

MMTC17a
in DMSO

Sample Name:
MMTC17a
Data Collected on:
400MR-vnmrs400
Archive directory: [illegible]

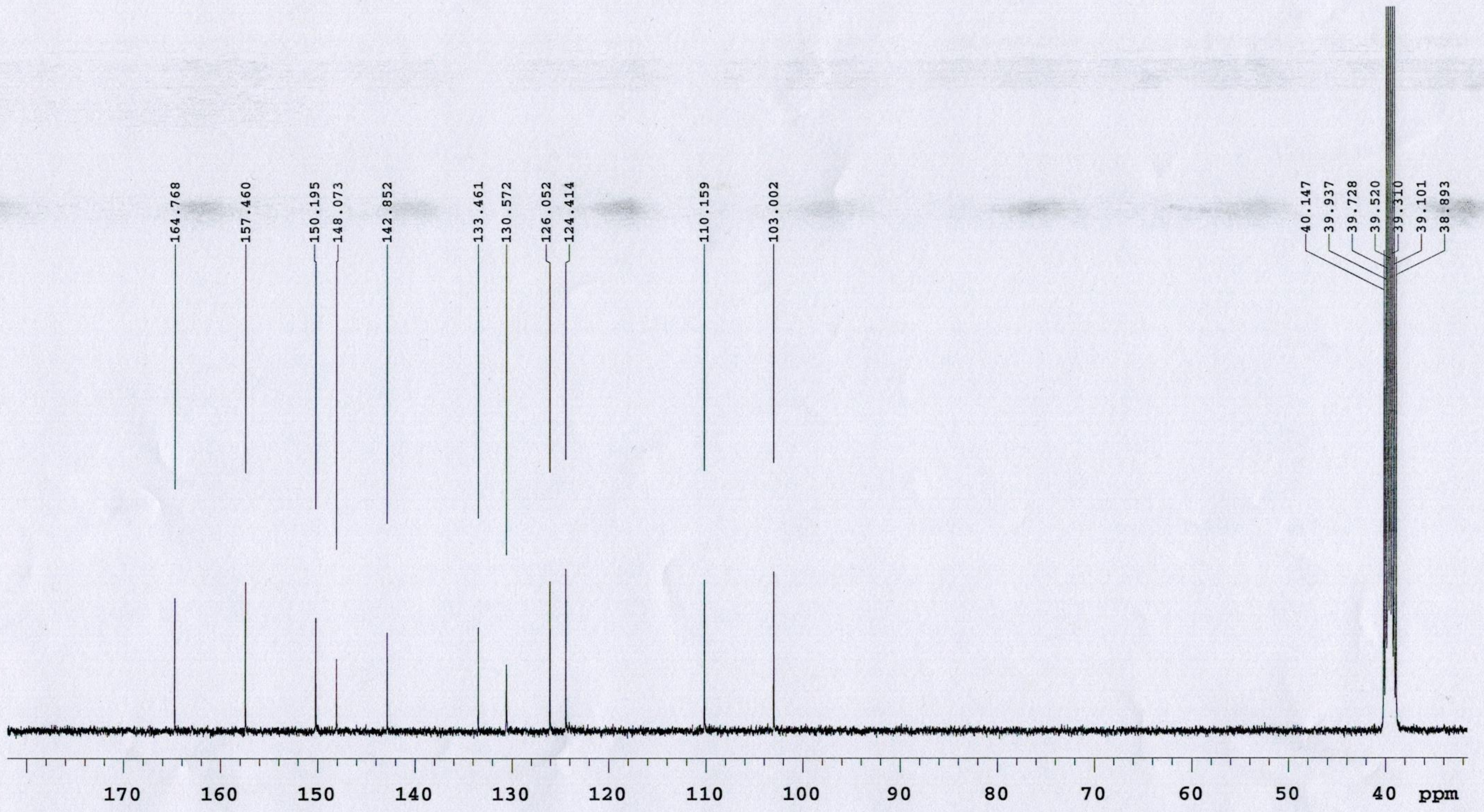

PULSE SEQUENCE
Relax. delay 1.500 sec
Pulse 38.5 degrees
Acq. time 2.674 sec
Width 24509.8 Hz
14000 repetitions

OBSERVE C13, 100.4576840
DECOUPLE H1, 399.5150667
Power 37 dB
continuously on
WALTZ-16 modulated

DATA PROCESSING
Line broadening 1.0 Hz
FT size 262144
Total time 16.2 hours

MMTC17a
in DMSO

Sample Name:
MMTC17a
Data Collected on:
400MR-vnmrs400
Archive directory: [illegible]

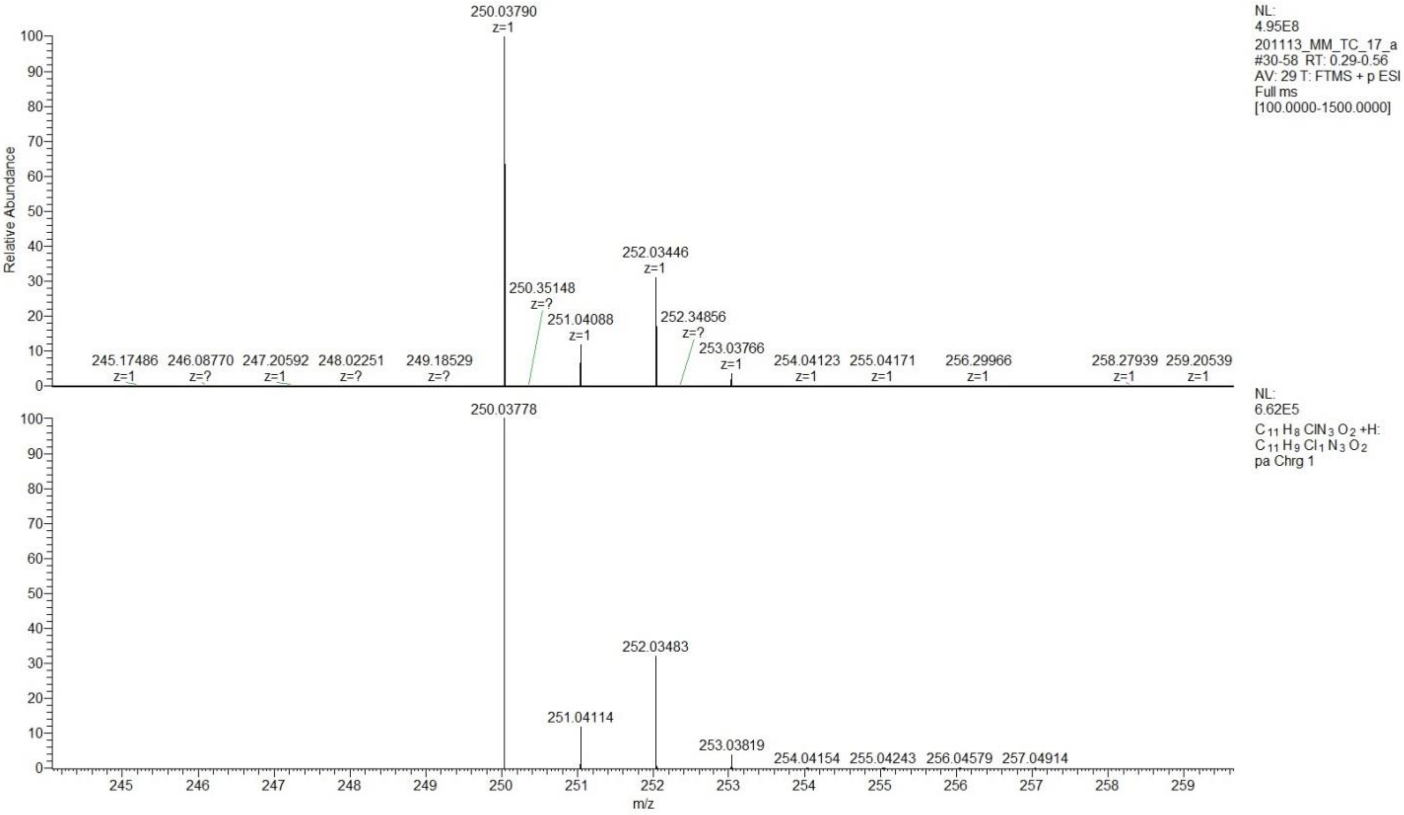

NL:
4.95E8
201113_MM_TC_17_a
#30-58 RT: 0.29-0.56
AV: 29 T: FTMS + p ESI
Full ms
[100.0000-1500.0000]
250.03790
z=1
250.35148
z=?
251.04088
z=1
252.03446
z=1
252.34856
z=?
253.03766
z=1
245.17486
246.08770
247.20592
248.02251
249.18529
254.04123
255.04171
256.29966
258.27939
259.20539
Relative Abundance
NL:
6.62E5
C 11 H 8 ClN 3 O 2 +H:
C 11 H 9 Cl 1 N 3 O 2
pa Chrg 1
250.03778
251.04114
252.03483
253.03819
254.04154
255.04243
256.04579
257.04914
m/z

Compound **TR-8**

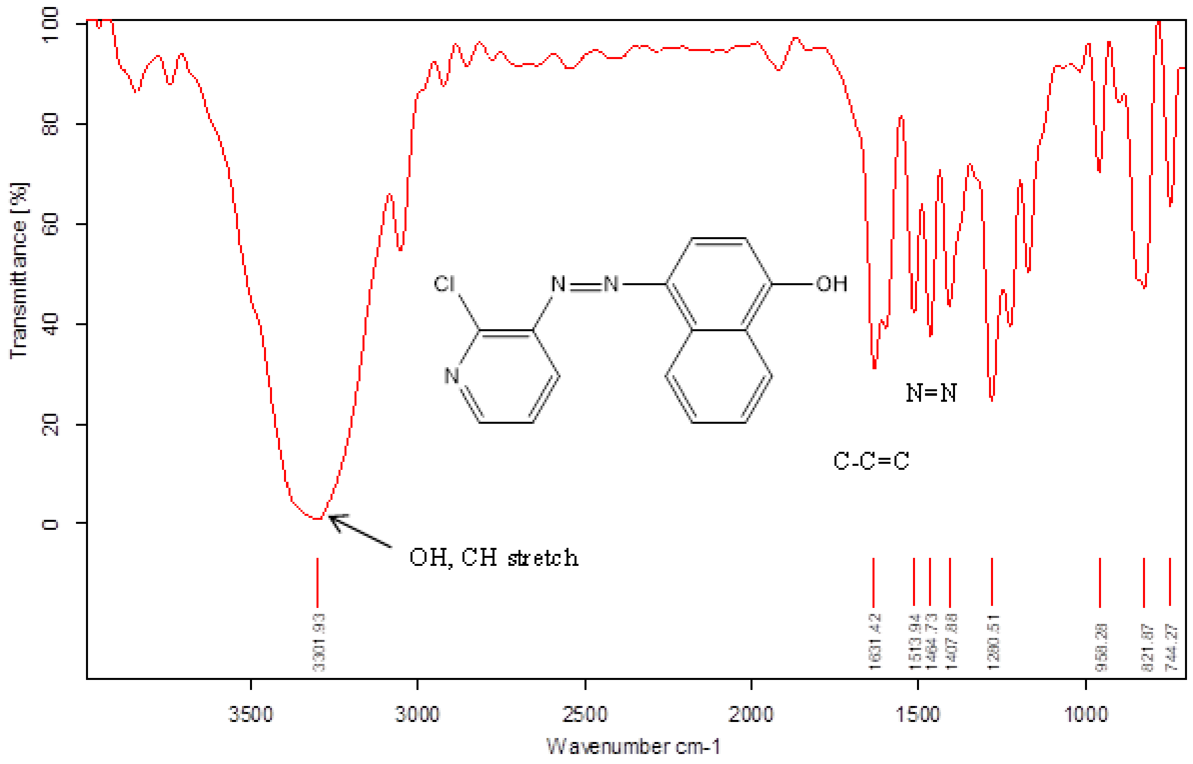

TC: 18	$C_{15}H_{10}ClN_3O$	283.71	IS	S	S	S	S	S	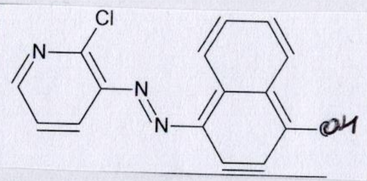

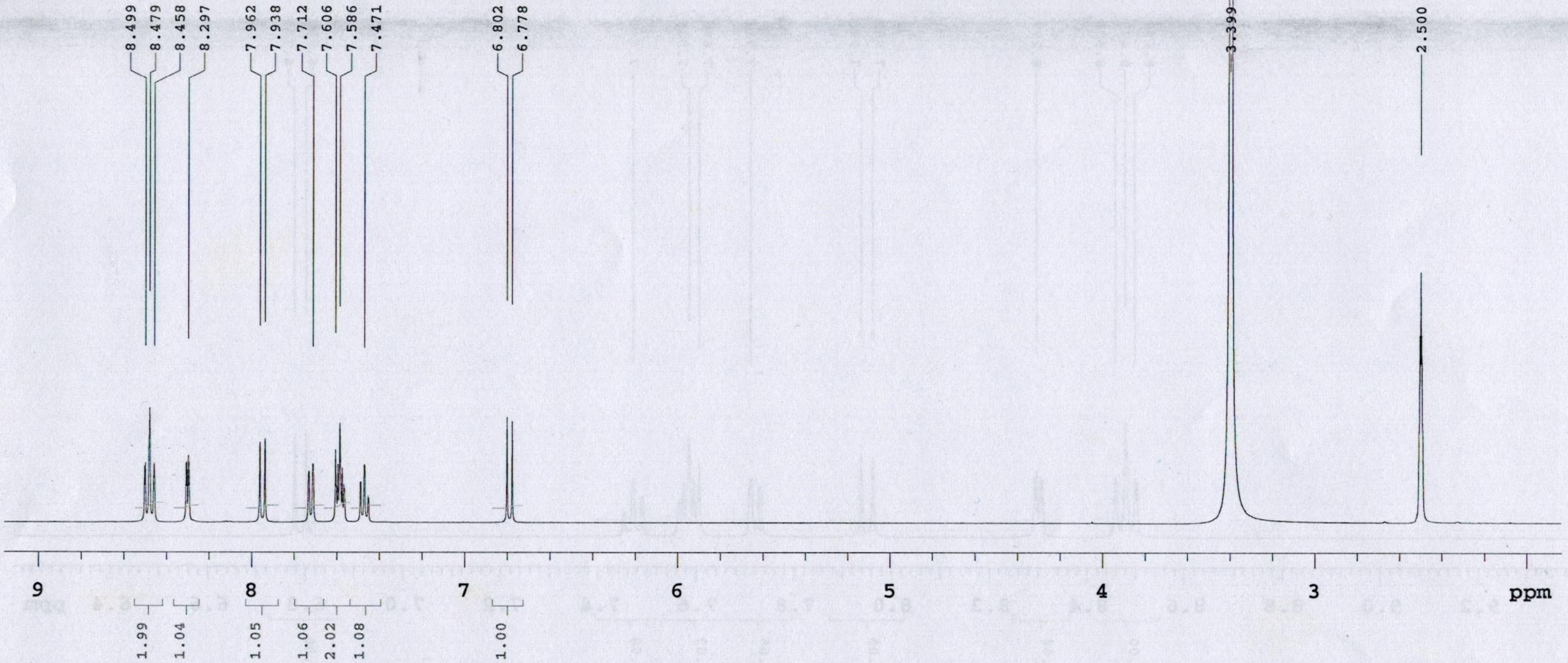

PULSE SEQUENCE
Relax. delay 0.500 sec
Pulse 48.6 degrees
Acq. time 4.797 sec
Width 6793.5 Hz
40 repetitions

OBSERVE H1, 399.5130590

DATA PROCESSING
FT size 65536
Total time 3 minutes

MMTC18a
in DMSO

Sample Name:
MMTC18a
Data Collected on:
400MR-vnmrs400
Archive directory: [illegible]

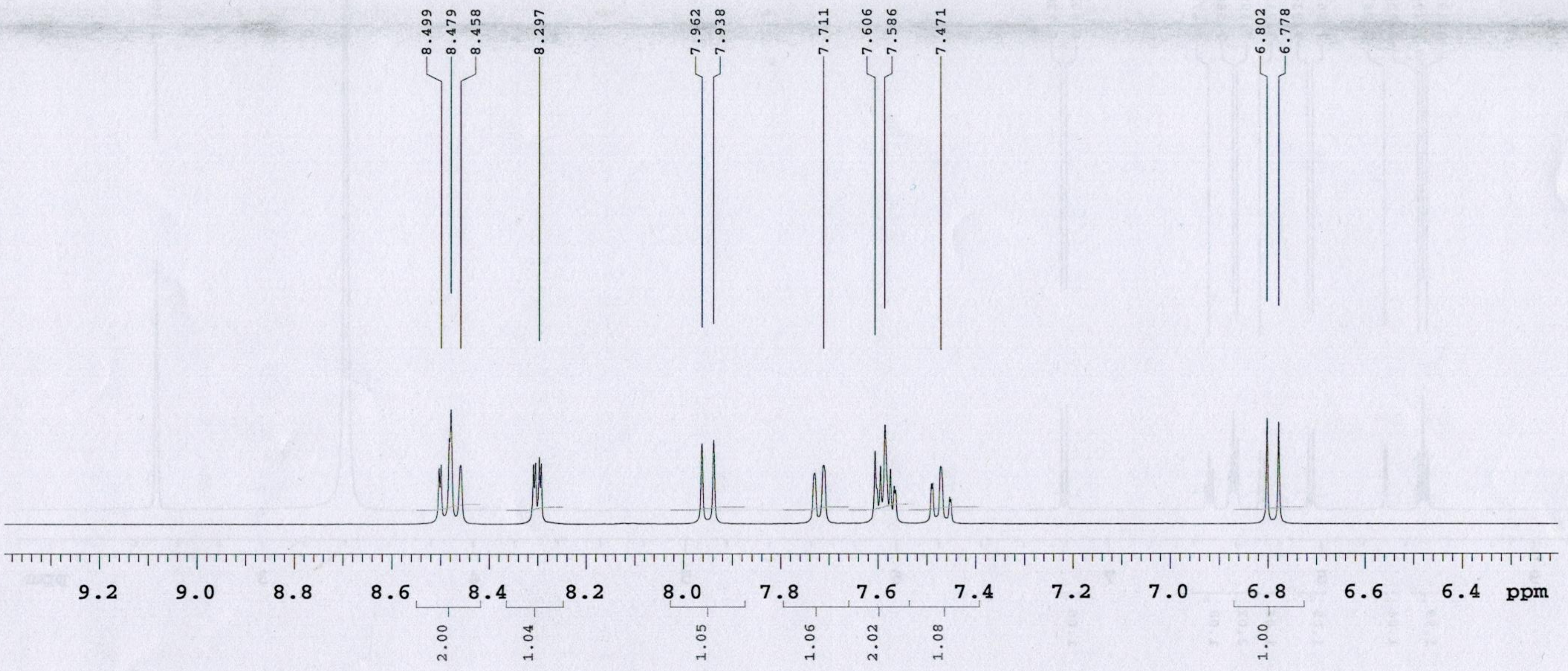

PULSE SEQUENCE
Relax. delay 0.500 sec
Pulse 48.6 degrees
Acq. time 4.797 sec
Width 6793.5 Hz
32 repetitions

OBSERVE H1, 399.5130590

DATA PROCESSING
FT size 65536
Total time 2 minutes

MMTC18a
in DMSO

Sample Name:
MMTC18a
Data Collected on:
400MR-vnmrs400
Archive directory: [illegible]

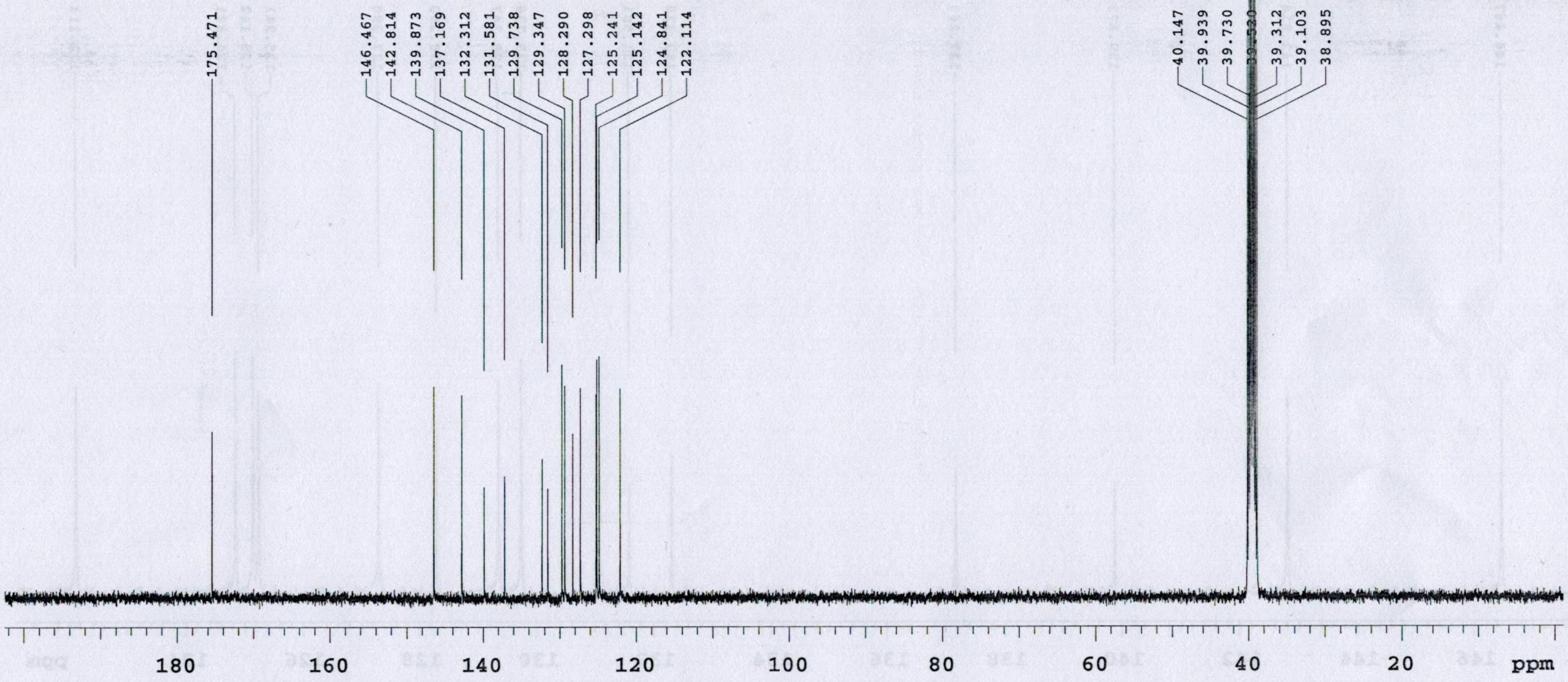

PULSE SEQUENCE
Relax. delay 1.500 sec
Pulse 38.5 degrees
Acq. time 2.674 sec
Width 24509.8 Hz
10000 repetitions

OBSERVE C13, 100.4576778
DECOUPLE H1, 399.5150667
Power 37 dB
continuously on
WALTZ-16 modulated

DATA PROCESSING
Line broadening 1.0 Hz
FT size 262144
Total time 11.6 hours

MMTC18a
in DMSO

Sample Name:
MMTC18a
Data Collected on:
400MR-vnmrs400
Archive directory: [illegible]

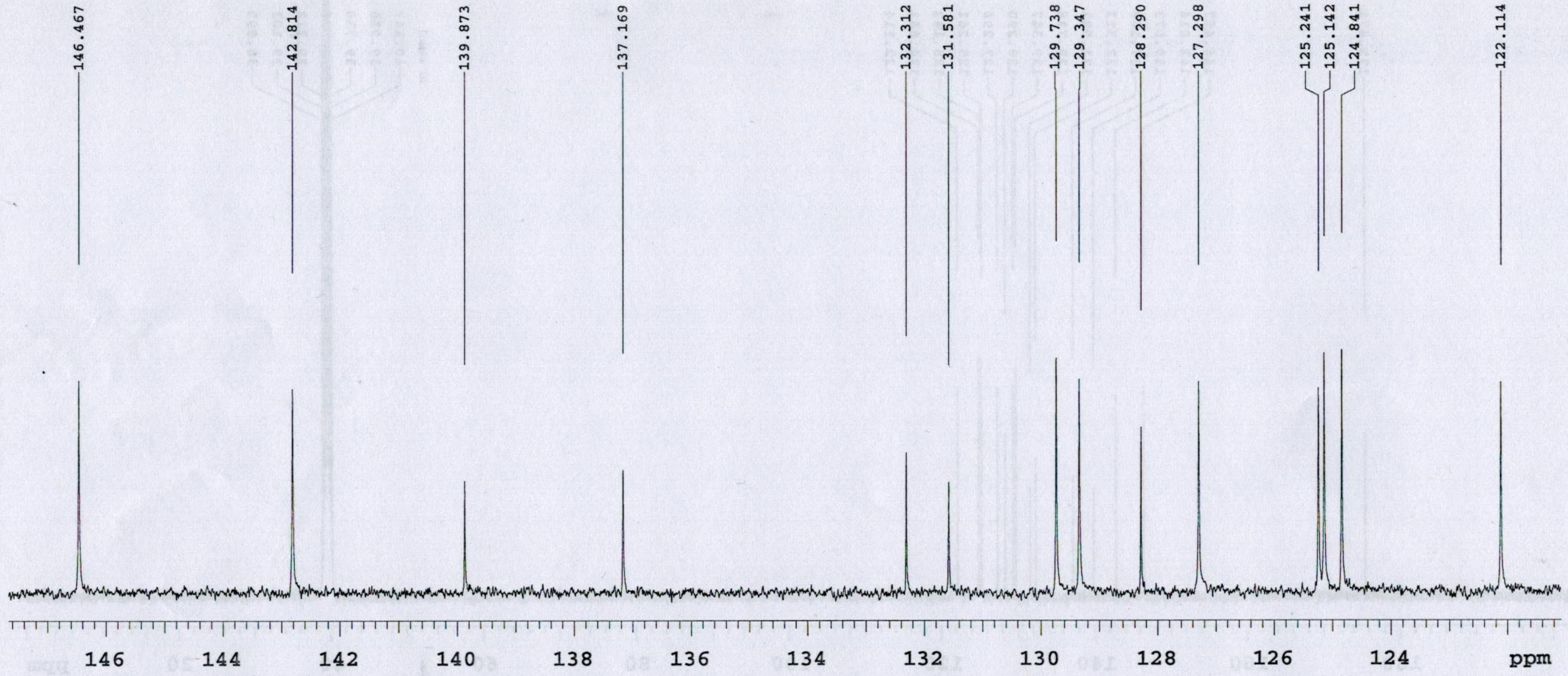

PULSE SEQUENCE
Relax. delay 1.500 sec
Pulse 38.5 degrees
Acq. time 2.674 sec
Width 24509.8 Hz
10000 repetitions

OBSERVE C13, 100.4576778
DECOUPLE H1, 399.5150667
Power 37 dB
continuously on
WALTZ-16 modulated

DATA PROCESSING
Line broadening 1.0 Hz
FT size 262144
Total time 11.6 hours

MMTC18a
in DMSO

Sample Name:
MMTC18a
Data Collected on:
400MR-vnmrs400
Archive directory:
[illegible]

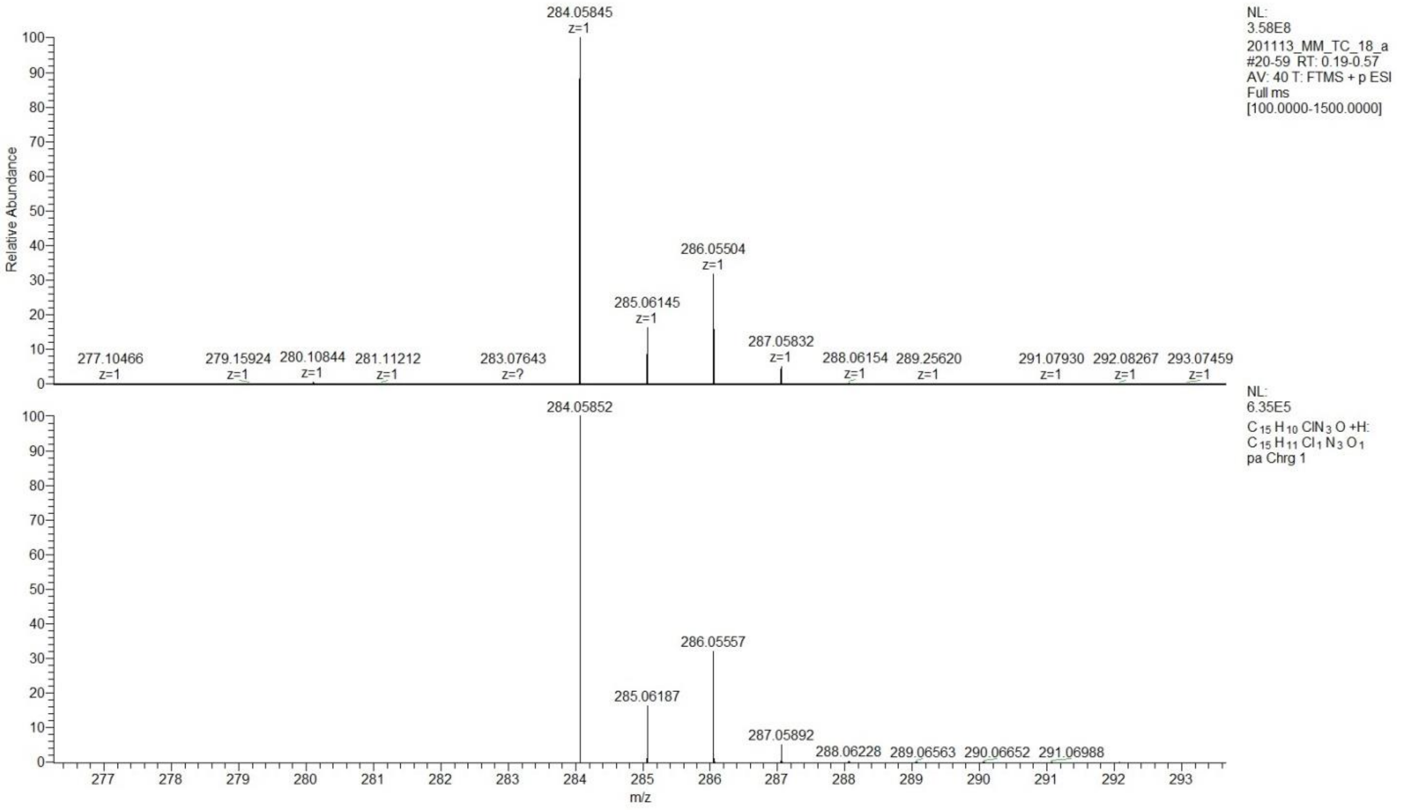
NL:
3.58E8
201113_MM_TC_18_a
#20-59 RT: 0.19-0.57
AV: 40 T: FTMS + p ESI
Full ms
[100.0000-1500.0000]
Relative Abundance
284.05845
z=1
286.05504
z=1
285.06145
z=1
287.05832
z=1
277.10466
z=1
279.15924
z=1
280.10844
z=1
281.11212
z=1
283.07643
z=?
288.06154
z=1
289.25620
z=1
291.07930
z=1
292.08267
z=1
293.07459
z=1
NL:
6.35E5
C 15 H 10 ClN 3 O +H:
C 15 H 11 Cl 1 N 3 O 1
pa Chrg 1
284.05852
286.05557
285.06187
287.05892
288.06228
289.06563
290.06652
291.06988
m/z

Compound **TR-9**

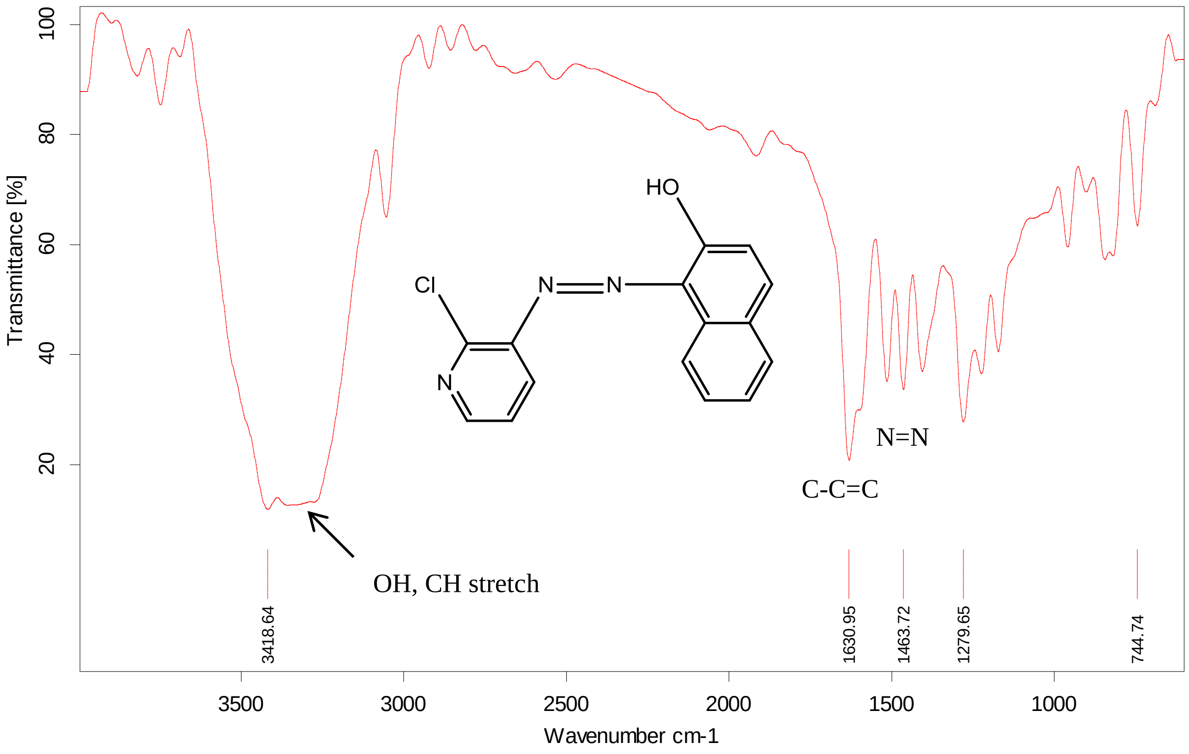

TC: 19	$C_{15}H_{10}ClN_3O$	283.71	IS	S	S	S	S	S	
									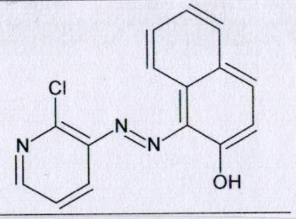

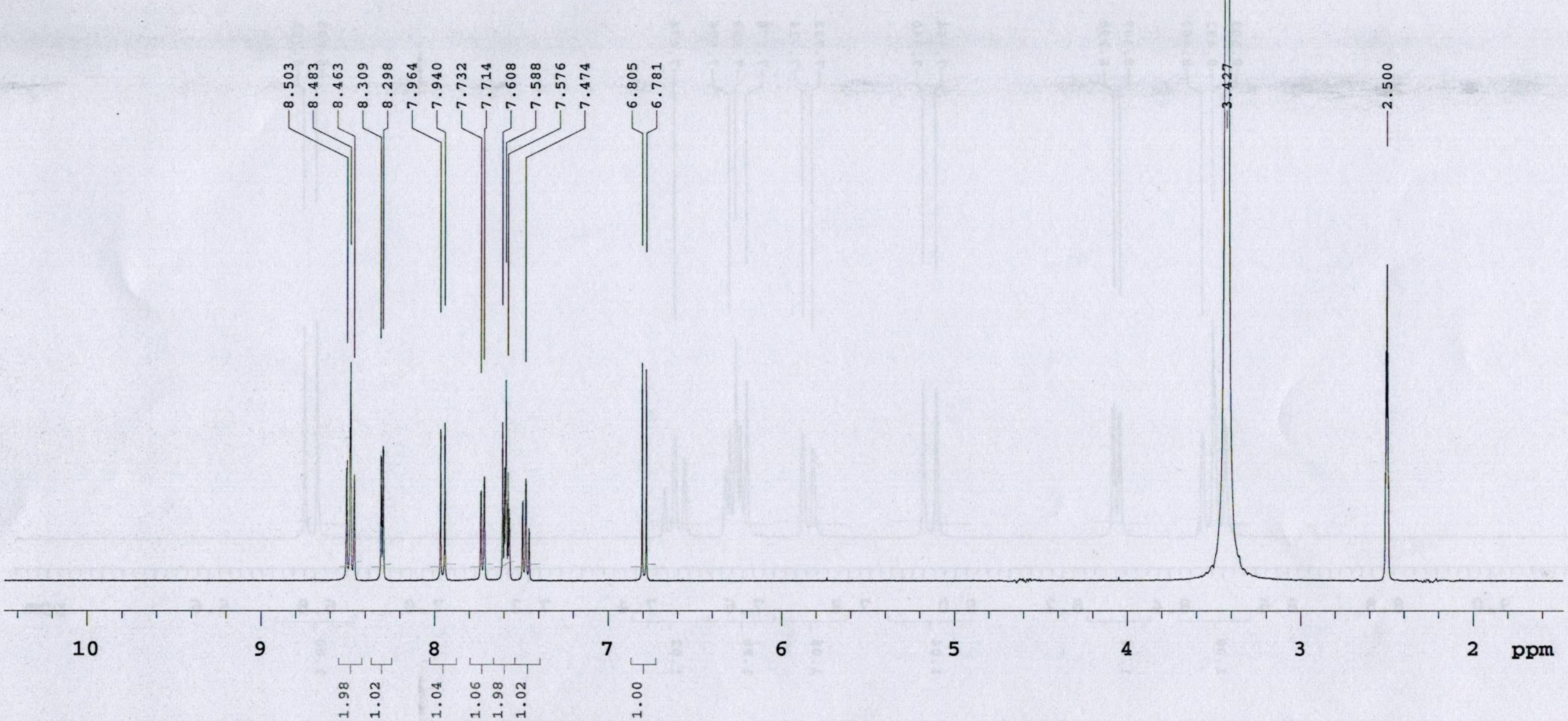

PULSE SEQUENCE
Relax. delay 0.500 sec
Pulse 48.6 degrees
Acq. time 4.797 sec
Width 6793.5 Hz
32 repetitions

OBSERVE H1, 399.5130590

DATA PROCESSING
FT size 65536
Total time 2 minutes

MMTC19a
in DMSO

Sample Name:
MMTC19a
Data Collected on:
400MR-vnmrs400
Archive directory: [illegible]

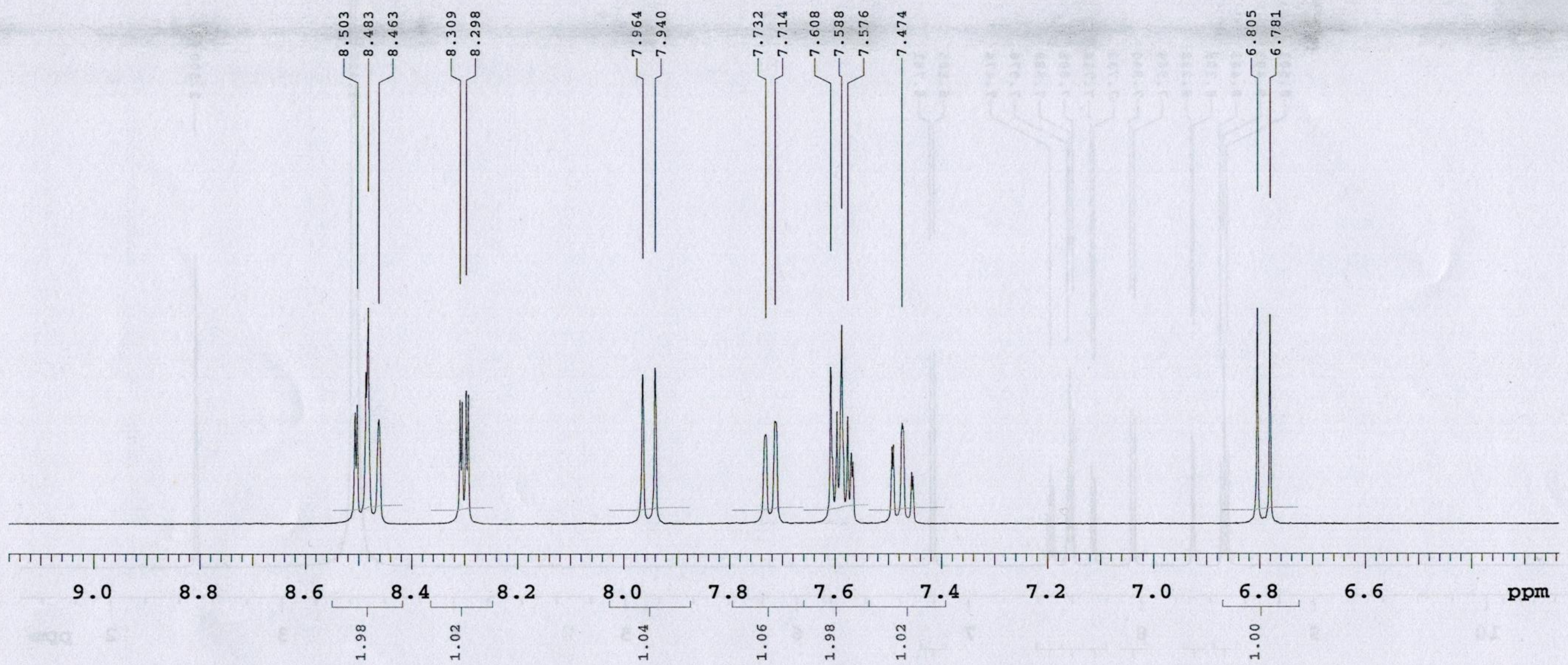

PULSE SEQUENCE
Relax. delay 0.500 sec
Pulse 48.6 degrees
Acq. time 4.797 sec
Width 6793.5 Hz
16 repetitions

OBSERVE H1, 399.5130590

DATA PROCESSING
FT size 65536
Total time 1 minute

MMTC19a
in DMSO

Sample Name:
MMTC19a
Data Collected on:
400MR-vnmrs400
Archive directory: [illegible]

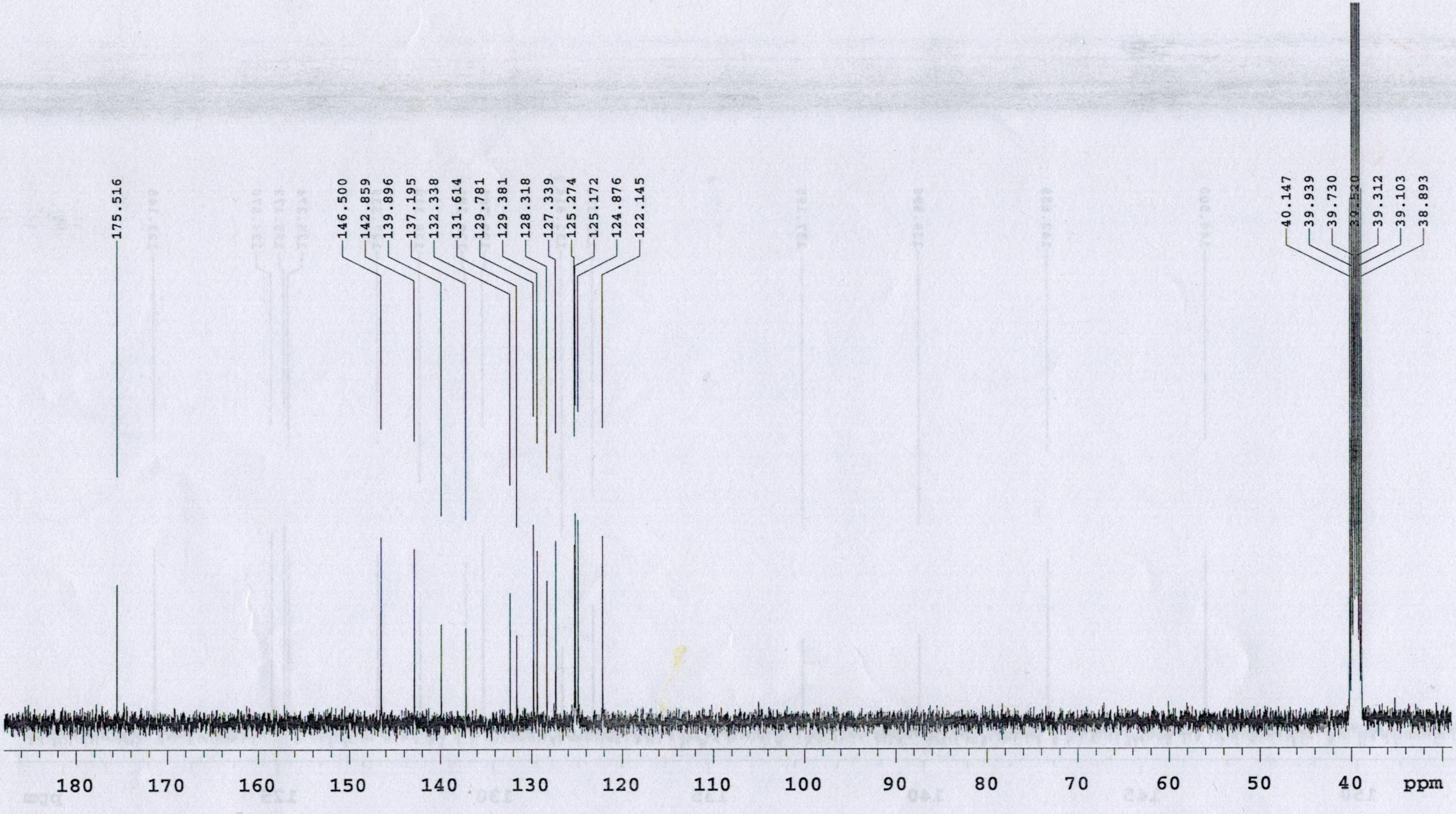

PULSE SEQUENCE
Relax. delay 1.500 sec
Pulse 38.5 degrees
Acq. time 2.674 sec
Width 24509.8 Hz
2304 repetitions

OBSERVE C13, 100.4576748
DECOUPLE H1, 399.5150667
Power 37 dB
continuously on
WALTZ-16 modulated

DATA PROCESSING
Line broadening 1.0 Hz
FT size 262144
Total time 2.7 hours

MMTC19a
in DMSO

Sample Name:
MMTC19a
Data Collected on:
400MR-vnmrs400
[illegible]

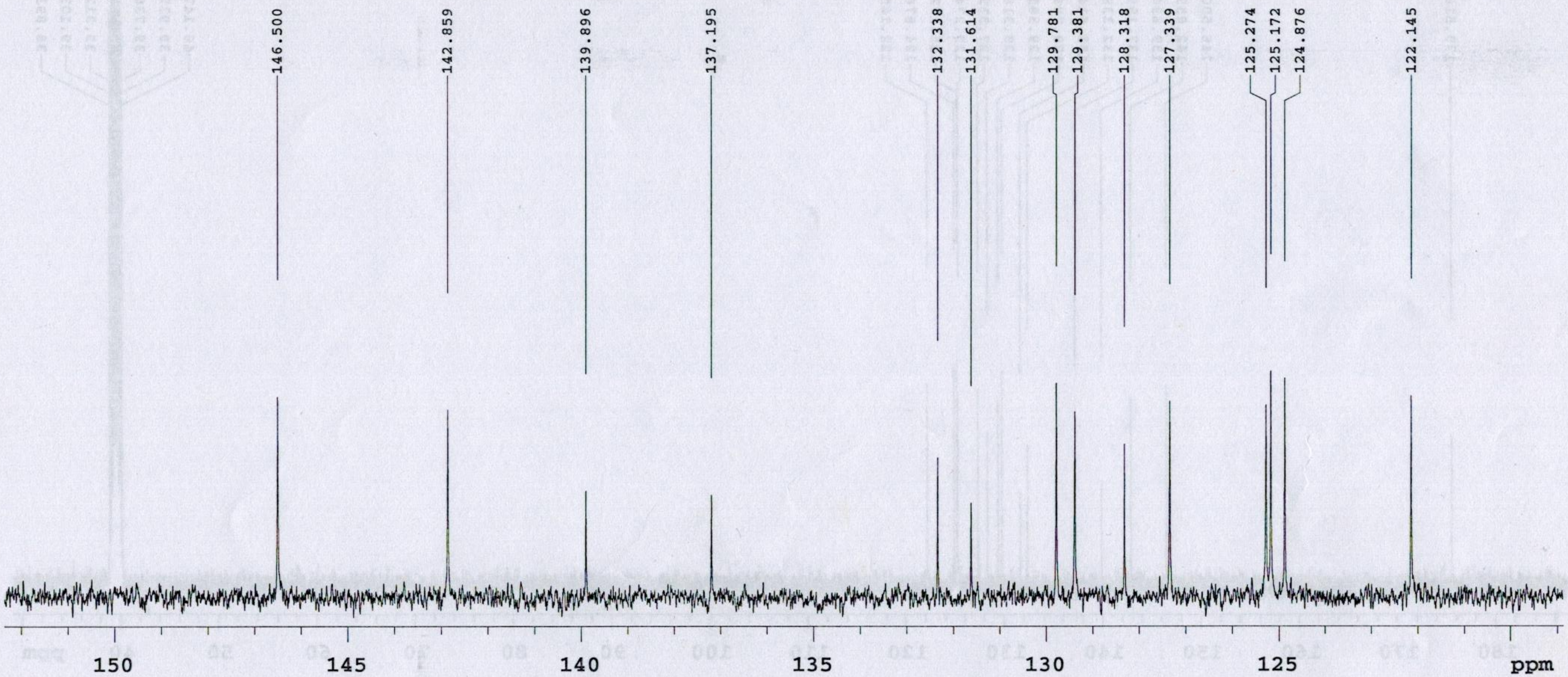

PULSE SEQUENCE
Relax. delay 1.500 sec
Pulse 38.5 degrees
Acq. time 2.674 sec
Width 24509.8 Hz
2320 repetitions

OBSERVE C13, 100.4576748
DECOUPLE H1, 399.5150667
Power 37 dB
continuously on
WALTZ-16 modulated

DATA PROCESSING
Line broadening 1.0 Hz
FT size 262144
Total time 2.7 hours

MMTC19a
in DMSO

Sample Name:
MMTC19a
Data Collected on:
400MR-vnmrs400
Archive directory:
[illegible]

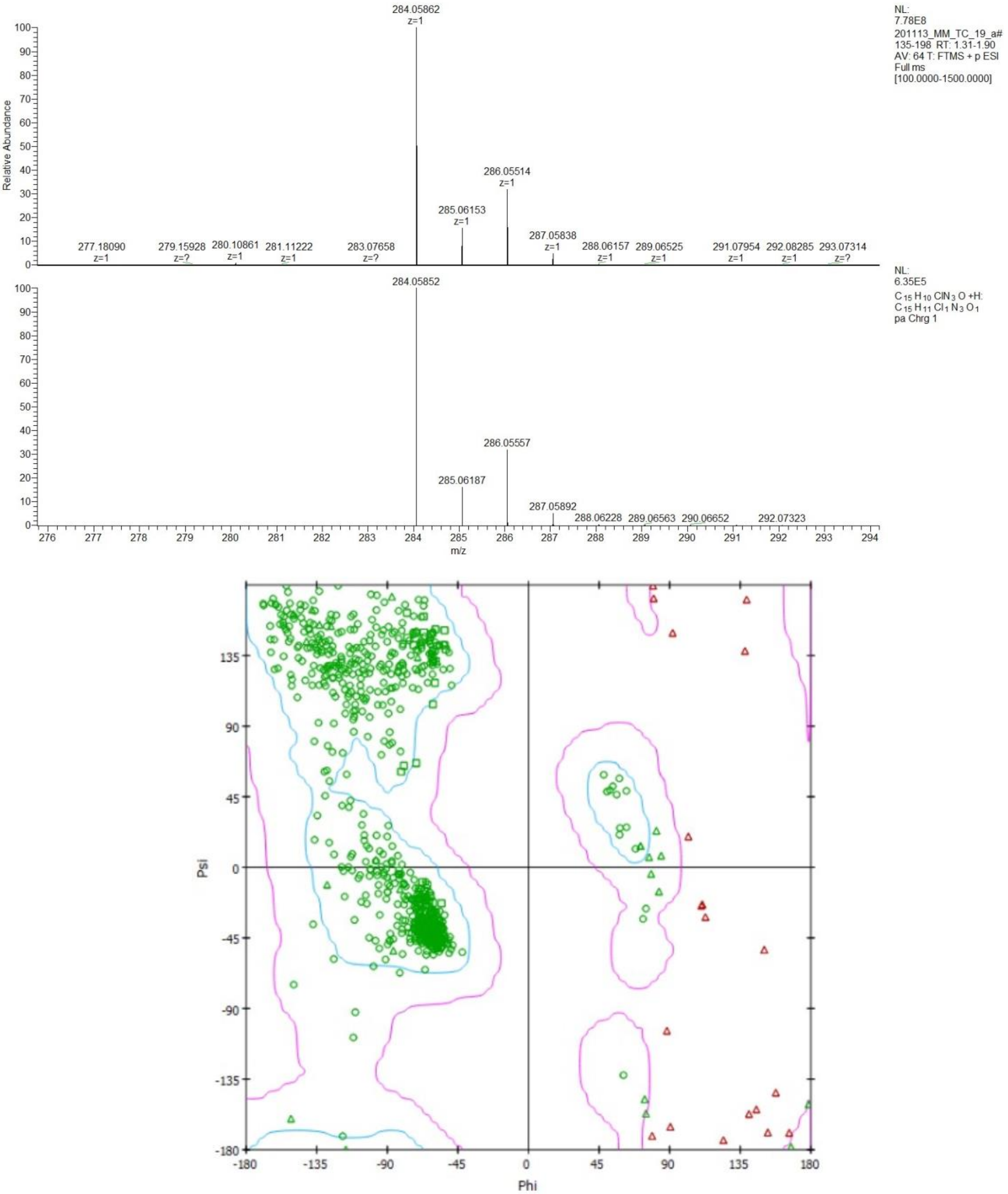

NL: 7.78E8
201113_MM_TC_19_a# 135-198 RT: 1.31-1.90 AV: 64 T: FTMS + p ESI Full ms [100.0000-1500.0000]
284.05862 z=1
286.05514 z=1
285.06153 z=1
287.05838 z=1
277.18090 z=1
279.15928 z=?
280.10861 z=1
281.11222 z=1
283.07658 z=?
288.06157 z=1
289.06525 z=1
291.07954 z=1
292.08285 z=1
293.07314 z=?
Relative Abundance
NL: 6.35E5
C 15 H 10 ClN 3 O +H: C 15 H 11 Cl 1 N 3 O 1 pa Chrg 1
284.05852
286.05557
285.06187
287.05892
288.06228
289.06563
290.06652
292.07323
m/z
Psi
Phi

Figure S1
